# Homocysteine Exacerbates Pulmonary Fibrosis via Orchestrating Syntaxin 17 Homocysteinylation of Alveolar Type II Cells

**DOI:** 10.1002/advs.202507803

**Published:** 2025-09-24

**Authors:** Jiefeng Huang, Ke Fang, Wuyan Lu, Rongrong Wu, Yuanyuan Chen, Linxiao Li, Zihao Hu, Zixin Cai, Yu Jiang, Xinli Fan, Jinyi Deng, Guangpeng Liu, Zhuang Luo, Lei Cui, Shuaijun Li

**Affiliations:** ^1^ Department of Plastic Surgery Shanghai Tenth People's Hospital Tongji University School of Medicine Shanghai 200072 China; ^2^ School of Medicine Tongji University Shanghai 200331 China; ^3^ Department of Radiology The First People's Hospital of Yunnan Province (Affiliated Hospital of Kunming University of Science and Technology) Kunming 650032 China; ^4^ Department of Respiratory and Critical Care Medicine The First Affiliated Hospital of Kunming Medical University Kunming 650032 China

**Keywords:** autophagy, folate, homocysteine, homocysteinylation, idiopathic pulmonary fibrosis

## Abstract

Idiopathic pulmonary fibrosis (IPF) is a lethal interstitial lung disease, marked by progressive extracellular matrix deposition, for which there are no effective treatments to halt disease progression. Although hyperhomocysteinemia is implicated in multiple pathological processes, its role in IPF remains largely unexplored. Through multiomics profiling of IPF patients, significantly elevated homocysteine (Hcy) concentrations in plasma and bronchoalveolar lavage fluid are identified compared to healthy controls. Single‐cell RNA sequencing and spatial transcriptomics reveal alveolar type 2 epithelial cells as the primary site of Hcy metabolism, with downregulation of Hcy‐catabolizing enzyme methionine synthase reductase (*MTRR*) during fibrotic progression. Genetic perturbation studies in murine models demonstrate that *MTRR* knockdown exacerbates bleomycin‐induced mortality and fibrosis, whereas *MTRR* overexpression exerts protective effects. Furthermore, Hcy supplementation initiates and accelerates pulmonary fibrosis development, while folate administration reduces pulmonary Hcy levels and alleviates fibrosis. Mechanistically, it is revealed that pathogenic hyperhomocysteinemia induces homocysteinylation–ubiquitination cascades that modify Syntaxin 17 (STX17) posttranslationally, leading to its proteasomal degradation and consequent impairment of autophagic flux. Notably, pharmacological folate administration reverses STX17 depletion, restoring autophagic flux and mitigating pulmonary fibrosis in mouse models. These findings collectively establish a Hcy–STX17–proteostasis axis wherein excess homocysteinylation creates a self‐reinforcing loop of autophagy dysfunction and fibrogenesis.

## Graphical abstract

The study identifies elevated homocysteine (Hcy) levels in idiopathic pulmonary fibrosis patients' samples, with alveolar type 2 cells as the primary site of Hcy metabolism. Methionine synthase reductase downregulation in these cells exacerbates fibrosis, while folate administration reduces Hcy levels and alleviates fibrosis. Mechanistically, Hcy drives Syntaxin 17 (STX17) degradation via homocysteinylation–ubiquitination, disrupting autophagy. Folate restores STX17 levels and mitigates fibrosis.

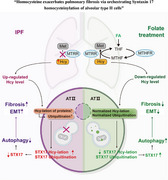

## 1 Introduction

Idiopathic pulmonary fibrosis (IPF) is a chronic interstitial lung disease characterized by rapid progression, poor prognosis, and short median survival, posing a serious threat to patients health. Clinical manifestations include tussiculation, dyspnea, and the decrease of pulmonary function.^[^ ^1^ ^]^ Current treatments, such as pirfenidone and nintedanib, only slow the progression of IPF but do not offer a complete cure.^[^ ^2^ ^]^ Therefore, identifying novel risk factors and preventive strategies is crucial for improving patient outcomes.

Alveolar type 2 (AT2) cells play a central role in the pathogenesis of lung fibrosis.^[^ ^3^, ^4^ ^]^ As progenitor cells responsible for regenerating alveolar type 1 (AT1) cells, AT2 cells are essential for maintaining alveolar integrity and function. However, under pathological conditions such as IPF, AT2 cells display abnormal phenotypes characterized by proliferation, apoptosis, senescence, and epithelial–mesenchymal transition (EMT).^[^ ^5^ ^]^ These maladaptive cells may become arrested in a transitional state or transdifferentiate into a basaloid cell state, leading to hyperplasia and metaplasia. Additionally, AT2 cells contribute to lung fibrosis by recruiting macrophages and regulating chemokines, thereby acting as driving factors in the disease's progression.^[^ ^6^, ^7^ ^]^ These researches collectively indicate that targeting the differentiation and function of AT2 cells can yield therapeutic benefits in treating fibrotic lung diseases.

AT2 cells are metabolically active, producing lipoprotein‐containing alveolar surfactant to maintain alveolar surface tension.^[^ ^8^ ^]^ However, metabolic imbalances, such as impaired mitochondrial fatty acid oxidation in AT2 cells, can lead to surfactant dysfunction and promote lung fibrosis.^[^ ^9^ ^]^ Homocysteine (Hcy) is a sulfur‐containing amino acid that does not form proteins and is synthesized from methionine as a crucial intermediate in the one‐carbon metabolic pathway.^[^ ^10^ ^]^ Hyperhomocysteinemia (HHcy), a metabolic disorder characterized by elevated levels of Hcy, is primarily caused by decreases in the 5‐methyltetrahydrofolate‐homocysteine methyltransferase reductase (*MTRR*) and Cystathionine Beta‐Synthase (*CBS*) genes, or from insufficient dietary intake of folate (Fol) or vitamin B12.^[^ ^11^, ^12^ ^]^ Research by Tripathi et al. demonstrated a correlation between serum Hcy levels and hepatic inflammation and fibrosis in nonalcoholic steatohepatitis, with knockout of the *CBS* gene leading to Hcy accumulation and exacerbating NASH progression.^[^ ^13^ ^]^ In addition, Fol is undoubtedly an essential factor in decreasing plasma Hcy level, and could be applied in the treatment with diseases correlated with HHcy.^[^ ^14^, ^15^ ^]^ However, the impact of altered Hcy metabolism in AT2 cells on pulmonary fibrosis, as well as the protective role of Fol in IPF, remains largely unknown, and the underlying mechanisms are yet to be elucidated.

Autophagy plays a vital role in the function and survival of AT2 cells.^[^ ^16^, ^17^ ^]^ Dysregulation of autophagy leads to increased apoptosis and senescence in AT2 cells, thereby impairing alveolar repair.^[^ ^18^ ^]^ This impairment in the repair process subsequently promotes the activation of fibroblasts, contributing to the progression of fibrosis. Conversely, enhancing autophagy through mechanisms such as the overexpression of *PLAC8* can improve AT2 cell survival and mitigate fibrosis.^[^ ^19^ ^]^ Previous work demonstrated that Hcy induced the homocysteinylation (Hcy‐lation) and ubiquitination of Syntaxin 17 (STX17), a key autophagosome/lysosome fusion protein, in NASH, leading to an autophagy blockade and promoting fibrosis.^[^ ^13^ ^]^ However, whether Hcy‐mediated modification of STX17 occurs in IPF, and its potential role in disease pathogenesis, remains unknown and warrants further investigation.

Here, we identified that Hcy catabolic genes were predominantly expressed in AT2 cells. Notably, during IPF progression, these genes especially *MTRR*, showed decreased expression specifically in AT2 cells, while maintaining stable expression levels in other cell types, thereby establishing AT2 cells as key regulators of Hcy metabolism. Furthermore, Hcy supplementation was found to accelerate pulmonary fibrosis. Importantly, Fol treatment was shown to reduce Hcy levels, inhibit the homocysteinylation of STX17, restore autophagic flux, and ultimately reverse lung fibrosis. These findings highlight the potential of Fol supplementation as an effective therapeutic strategy for IPF.

## 2 Results

### 2.1 Elevated Hcy Levels Is a Potential Risk Factor for IPF

**Figure 1:**
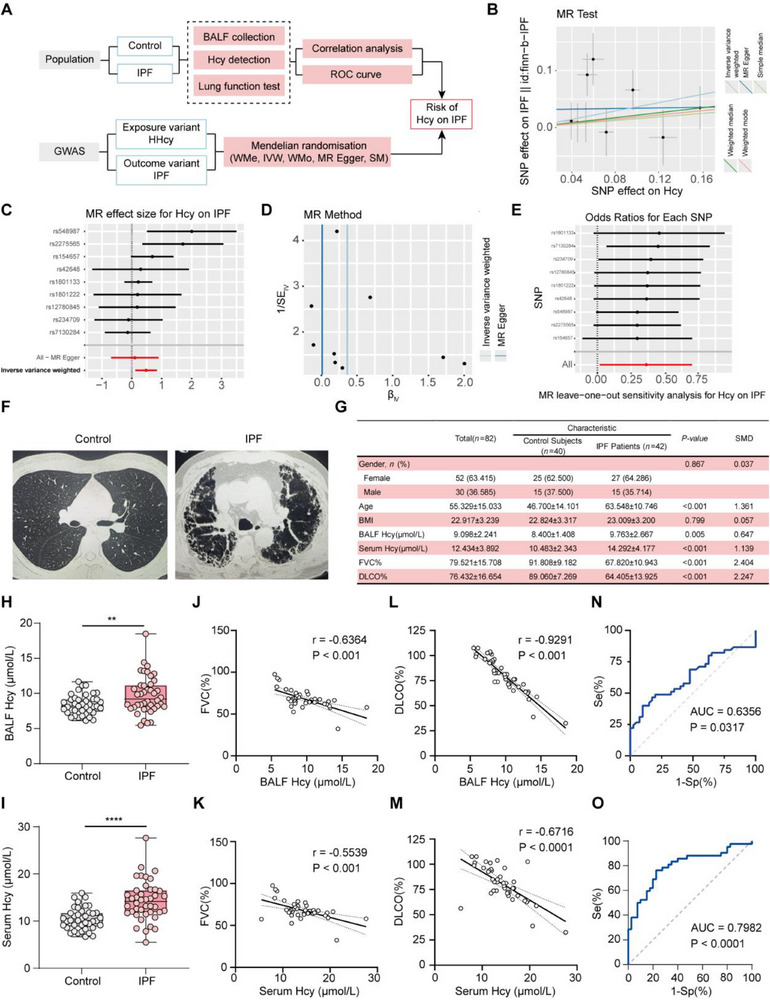
MR and case control study reveals potential risk of Hcy on IPF. A) Analysis flowchart showing the steps of the experiment. B) Scatter plot showing positive relationship between Hcy and IPF. C) Forest plot showing positive MR effect size for Hcy on IPF. D) Heterogeneity of SNPs demonstrated by funnel plot. E) “Leave‐one‐out” sensitivity analysis results demonstrating by odds ratio. F) Computed tomography (CT) images showing IPF patients’ imaging characteristics. G) Clinical and pathological characteristics of cases with or without diagnosis of IPF. Statistical significance tests: gender, Pearson χ2 test; age, BMI and FVC (%), Two independent‐sample *t*‐test; Hcy and DLCO%, Welch's *t*‐test. H,I) BALF Hcy level and serum Hcy level in healthy control (*n* = 40) and IPF patients (*n* = 42). J,K) Correlation analysis of BALF Hcy level or serum Hcy level and FVC (%). L,M) Correlation analysis of BALF Hcy level or serum Hcy level and DLCO (%). N,O) Receiver operating characteristic (ROC) curve revealed prognostic value of Hcy level for IPF (cutoff value = 10.14 µmol L^−1^) and ROC curve revealing robust diagnostic value of serum Hcy (cutoff value = 11.85 µmol L^−1^). Data are presented as mean ± standard deviation (SD) or number (%). MR: Mendelian randomization; BMI: body mass index; BALF: bronchoalveolar lavage fluid; FVC: forced vital capacity; DLCO: diffusing capacity of the lung for carbon monoxide.

To investigate the role of Hcy in IPF pathogenesis, we performed a comprehensive Mendelian randomization (MR) analysis and measured Hcy levels in both plasma and bronchoalveolar lavage fluid (BALF) from IPF patients (**Figure** **1**A). Consistent associations were observed across multiple single nucleotide polymorphisms (SNPs), indicating a positive correlation with IPF risk (Figure 1B,C). A funnel plot illustrated the heterogeneity among the included SNPs (Figure 1D). Furthermore, the odds ratio for the effect of Hcy on IPF was significantly greater than 1, reinforcing the positive association between Hcy levels and IPF risk (Figure 1E). Our MR analysis indicates a potential causal relationship between elevated Hcy levels and increased risk of IPF, although further clinical studies are warranted to confirm this link.

Subsequently, we analyzed Hcy levels in the plasma and BALF of IPF patients compared to healthy individuals. According to our inclusion criteria, BALF samples were collected from 40 IPF patients and 42 healthy subjects (Figure 1F,G). As anticipated, IPF patients exhibited significantly lower forced vital capacity (FVC) and diffusing capacity of the lung for carbon monoxide (DLCO) compared to relatively healthy individuals (*p* < 0.05) (Figure 1G). Notably, Hcy levels in plasma and BALF were substantially elevated in IPF patients (Figure 1H,I). These elevated Hcy levels were significantly inversely correlated with lung function, as measured by FVC and DLCO (Figure 1J–M). Receiver operating characteristic (ROC) curve analyses were performed to evaluate the diagnostic potential of Hcy levels in both BALF and plasma samples. BALF Hcy showed moderate diagnostic accuracy (AUC = 0.6356, *p* = 0.031), with an optimal cutoff value of 10.14 µmol L^−1^ for identifying increased IPF risk (Figure 1N). Plasma Hcy showed similar trend in IPF and better diagnostic performance (AUC = 0.7982, *p* < 0.0001), with an optimal threshold of 11.85 µmol L^−1^ (Figure 1O). Together, these results suggest that elevated Hcy levels could be a potential risk factor for IPF.

### 2.2 Hcy Metabolism Is Impaired in Pathogenesis of IPF

**Figure 2:**
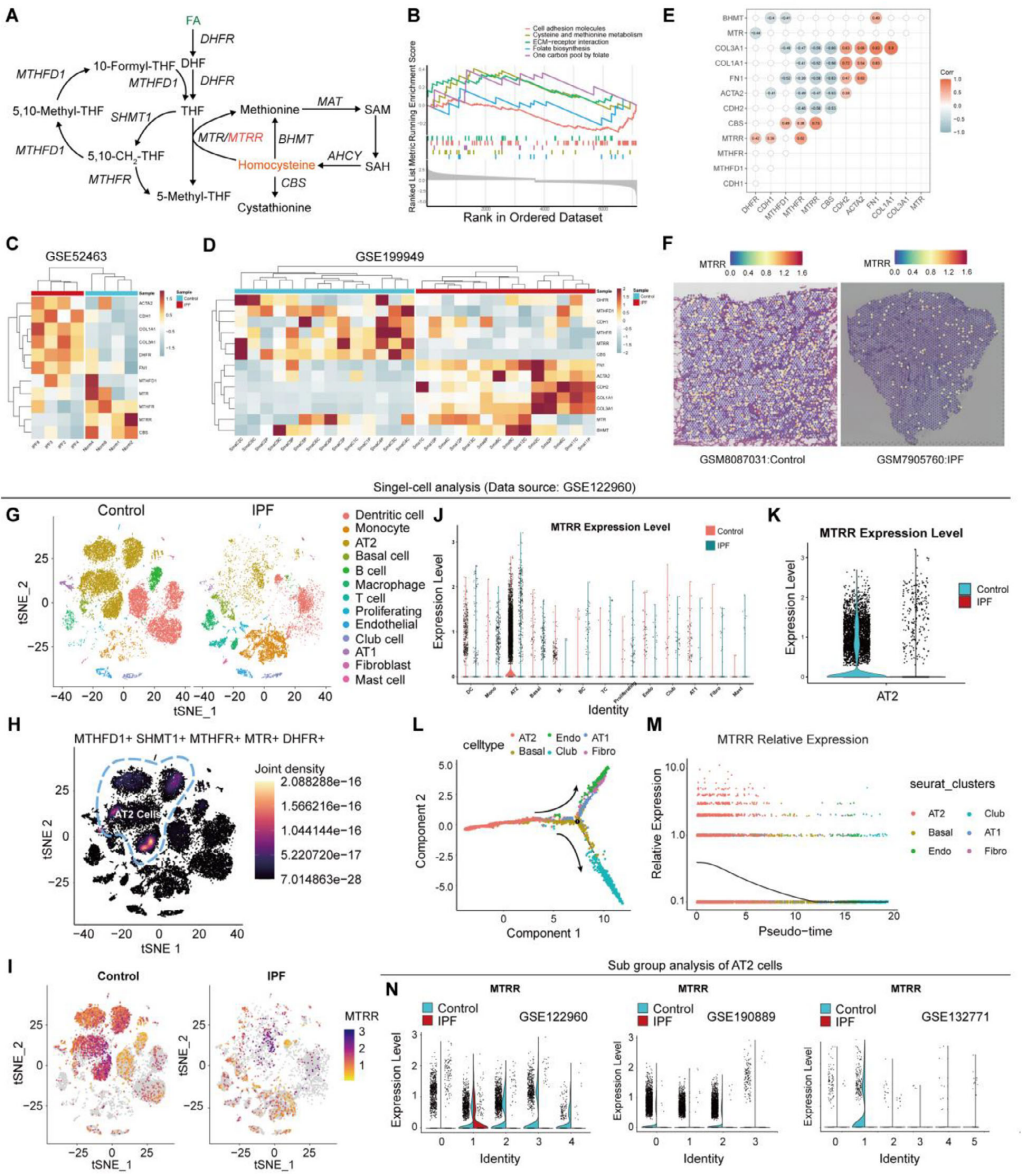
Bulk‐seq and scRNA‐seq analysis of human IPF samples showing weakened Hcy metabolism in IPF. A) Schematic diagram of Hcy metabolism. B) GSEA showed the enrichment of fibrosis and Hcy metabolism terms in IPF group. C,D) Heatmap of enzymes (*MTR*, *MTRR*, *MTHFR*, *MTHFD1*, *CBS*, *BHMT*) involved in Hcy metabolism and fibrosis markers (*COL1A1*, *COL3A1*, *ACTA2*, *FN1*, *CDH2*). Data source: GSE52463 (C), GSE199949 (D). E) Correlation analysis between the expression of fibrosis and Hcy metabolism related genes in IPF environment. F) Spatial feature plots showing the distinct expression patterns of *MTRR* in control and IPF samples. G) T‐distributed stochastic neighbor embedding (t‐SNE) plot showing 13 distinct clusters resulting from scRNA‐seq of cells derived from lung tissues harvested from IPF and non‐IPF group. H) Joint density plot of Hcy metabolism genes created by scCustomize R package. I) Comparison of *MTRR* expression in all cells between control and IPF group demonstrated by t‐SNE plot. J) Violin plot showing the expression of *MTRR* among different cell types. K) Violin plot with dots showing the expression of *MTRR* in AT2 cells in control and IPF group. L) Pseudotime trajectory analysis of lung cells (AT1: alveolar type 1 cell; AT2: alveolar type 2 cell; Endo: endothelial cell; Basal: basal cell; Club: club cell; Fibro: fibroblast). M) *MTRR* relative expression with the progression of pseudotime in lung cells. N) Violin plot showing *MTRR* expression in different sub‐type cells of AT2 in control and IPF group (data source: GSE122960, GSE190889, GSE132771).

To investigate the potential role of Hcy in the pathogenesis of IPF, we analyzed the expression patterns of Hcy metabolism genes in lung tissues from IPF patients and healthy subjects using the Gene Expression Omnibus (GEO) database (**Figure** **2**A; Figure S1A–C, Supporting Information). Gene Ontology (GO) and Kyoto Encyclopedia of Genes and Genomes (KEGG) enrichment analyses revealed upregulation of EMT‐associated pathways in IPF, including focal adhesion, protein serine/threonine kinase activity, and transforming growth factor beta response. Conversely, Hcy‐related metabolic pathways, specifically cysteine and methionine metabolism and one‐carbon pool by folate, were downregulated (Figure S1D–H, Supporting Information). Gene Set Enrichment Analysis (GSEA) further confirmed the downregulation of these metabolic pathways in IPF (Figure 2B). Heatmap and volcano plot analyses of three datasets demonstrated significant reduction in Hcy catabolism genes, including genes of *MTRR*, 5‐methyltetrahydrofolate homocysteine methyltransferase (*MTR*), methylenetetrahydrofolate reductase (*MTHFR*), methionine adenosyltransferase 1A (*MAT1A*), and *CBS* in IPF tissues (Figure 2C,D; Figure S1A–C, Supporting Information). This reduction coincided with increased expression of fibrosis‐associated genes, such as *TGFB*, *COL1A1*, *COL3A1*, *ACTA2*, and *FN1*. Protein–protein interaction (PPI) network analysis revealed potential interactions between fibrotic proteins and Hcy catabolism enzymes, while correlation analysis demonstrated an inverse relationship between Hcy metabolism genes and fibrotic genes, implying that Hcy metabolism is suppressed during IPF progression (Figure 2E; Figure S1I, Supporting Information). Spatial transcriptomics further confirmed inhibited expression of the core Hcy metabolic gene *MTRR* in IPF tissues (Figure 2F).

Given the cellular complexity of lung tissue and its alterations in fibrosis, we integrated single‐cell RNA sequencing (scRNA‐seq) data from the GEO database to complement our bulk transcriptomic findings (Figure S2A,B, Supporting Information). We identified 12 major cell types, including dendritic cells, alveolar epithelial type II cells, monocytes, basal cells, macrophages, B cells, T cells, proliferating cells, endothelial cells, club cells, alveolar epithelial type I cells, and fibroblasts (Figure 2G). While cellular cluster compositions were broadly similar between healthy and IPF samples, significant differences were observed in relative cell numbers, particularly a decrease in AT2 cells and an increase in monocytes in IPF tissue (Figure S2C, Supporting Information). Notably, Hcy metabolic genes, including *MTRR*, *MTR*, *MTHFR*, and *MAT1A*, were predominantly expressed in AT2 cells (Figure 2H). During IPF progression, AT2 cells showed reduced expression of these genes, particularly *MTRR*, while expression levels in other cell types remained unchanged (Figure 2I–K; Figure S2D–F, Supporting Information). This finding highlights AT2 cells as primary responders in Hcy metabolism regulation. Subsequent transcriptional analysis revealed heterogeneity within the AT2 cell population, leading to the identification of five distinct subsets, each defined by unique marker gene (Figure S3E,F, Supporting Information). GO enrichment analysis revealed distinct gene expression patterns and pathway enrichment among these subsets, indicating diverse functional roles (Figure S3G, Supporting Information). Pseudotime analysis demonstrated progressive decline in *MTRR* expression accompanied by reduced AT2 cell numbers (Figure 2L,M; Figure S3A–D, Supporting Information). Consistently, all AT2 subpopulations in IPF samples showed reduced *MTRR* expression compared to controls, a trend validated across multiple scRNA‐seq cohorts (Figure 2N). Immunofluorescence costaining for surfactant protein (SP)‐C and *MTRR* confirmed AT2‐specific *MTRR* expression in healthy lungs and its marked downregulation in IPF (Figure S3H,I, Supporting Information).

**Figure 3:**
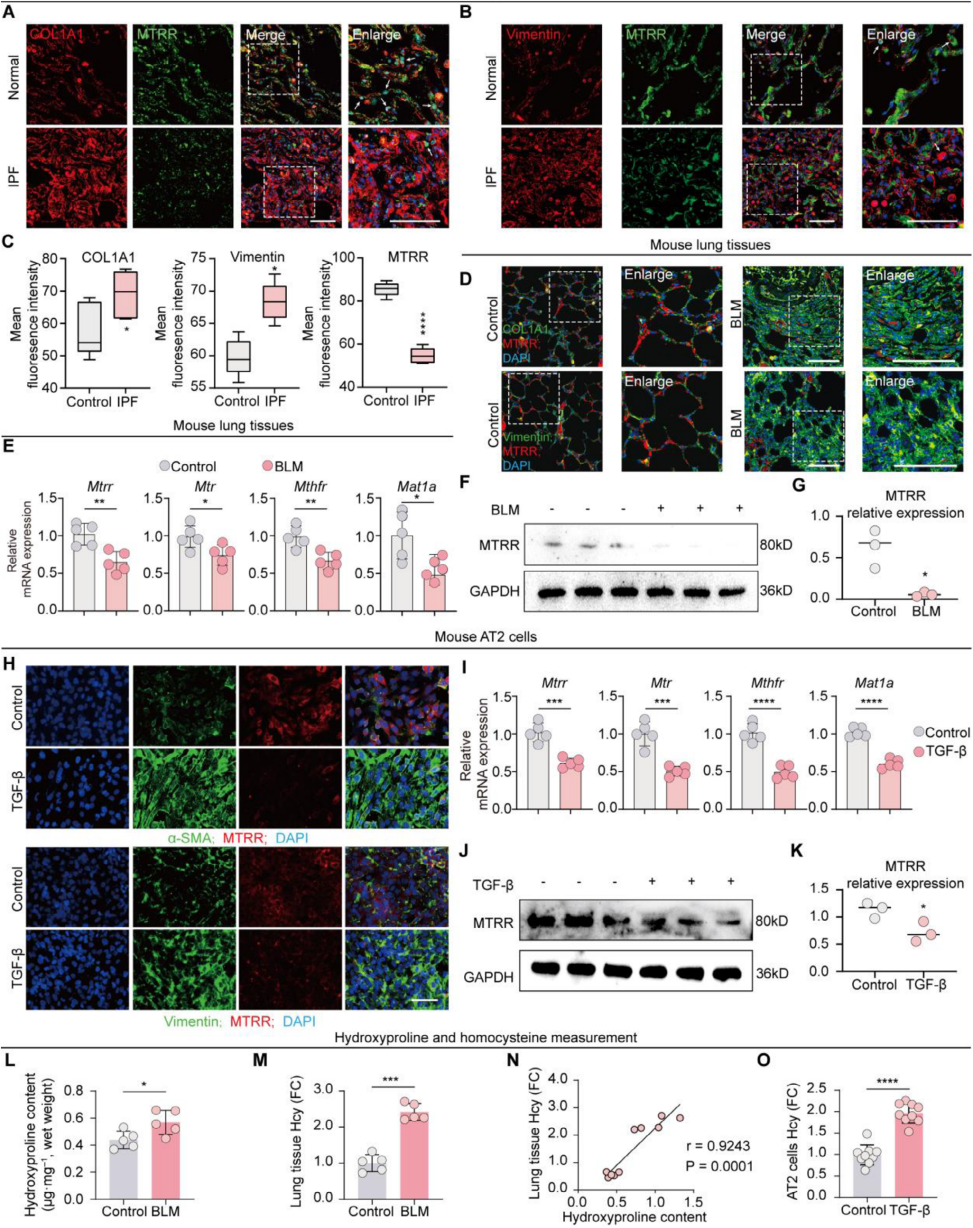
Hcy metabolism is disrupted in the IPF state. A,B) Immunofluorescence costaining of *COL1A1* (A) and vimentin (B) with *MTRR* in lung sample slices from IPF patients and normal control (*n* = 3 per group). Right‐hand panels display magnified areas from images indicated by dashed boxes. White arrows indicated AT2 cells with higher expression of *MTRR* stained into light blue. Scale bar = 100 µm. C) Quantitative analysis of mean fluorescence intensity of *COL1A1*, Vimentin, and *MTRR* (*n* = 5). D) Costaining of *COL1A1* and vimentin with *MTRR* in mouse lung tissues (*n* = 5). Scale bar = 100 µm. E) Main Hcy metabolic enzymes (*MTRR*, *MTR*, *MTHFR*, *MAT1*A mRNA levels were quantified by qRT‐PCR (*n* = 5 per group). F) Lung tissues from mice treated with BLM (5.0 mg kg^−1^ body weight) were subjected to western blotting, showing the expression level of *MTRR* (*n* = 3). G) Quantification of *MTRR* protein expression level (*n* = 3). H) *MTRR* in human AT2 cells treated with TGF‐β (10 ng mL^−1^) were costained with *COL1A1* and vimentin (*n* = 5 per group). Scale bar, 50 µm. I) Quantitative PCR analysis of *MTRR*, *MTR*, *MTHFR*, and *MAT1A* mRNA expression level in AT2 cells (*n* = 5). J) TGF‐β (10 ng mL^−1^)‐treated‐AT2 cells were lysed for western blotting (*n* = 3 per group). K) Quantification of *MTRR* protein expression detected by western blot (*n* = 3). L) Hydroxyproline level measured in lung homogenates from BLM (5.0 mg kg^−1^ body weight) challenged mice. M) Hcy level measured in lung homogenates from BLM (5.0 mg kg^−1^ body weight) challenged mice. N) Correlation analysis between Hcy concentration and hydroxyproline level in mouse lung tissues. O) Hcy level measured in AT2 cell lysis after TGF‐β treatment (10 ng mL^−1^). Data are presented as the mean ± standard error of the mean (SEM). *p < 0.05; **p < 0.01; ***p < 0.001; ****p < 0.0001 by Student's *t*‐tests.

To further validate the dysregulation of Hcy metabolism in IPF, we investigated the expression levels of Hcy metabolic genes in patient tissues and the bleomycin (BLM)‐induced mouse model. IPF samples exhibit substantial tissue remodeling, particularly through EMT. Costaining analysis with vimentin and *COL1A1* revealed significantly reduced *MTRR* expression in AT2 cell (showed by white arrow) in IPF patients (**Figure** **3**A–C). Similarly, key metabolic genes including *MTRR*, *MTR*, *MTHFR*, and *MAT1A* were significantly downregulated in BLM‐treated lungs (Figure 3D–G), accompanied by increased Hcy levels that positively correlated with hydroxyproline levels (Figure 3L–N). We also treated freshly isolated mouse AT2 cells with TGF‐β1, a central profibrotic cytokine. TGF‐β1 treatment markedly suppressed the expression of these metabolic genes and increased intracellular Hcy levels (Figure 3H–K,O). These results from human IPF samples, BLM‐challenged mice, and TGF‐β1‐treated AT2 cells consistently demonstrate disrupted Hcy metabolism and subsequent Hcy accumulation as hallmark features of pulmonary fibrosis.

### 2.3 Defective Hcy Catabolism Promoted the Occurrence and Development of IPF

**Figure 4:**
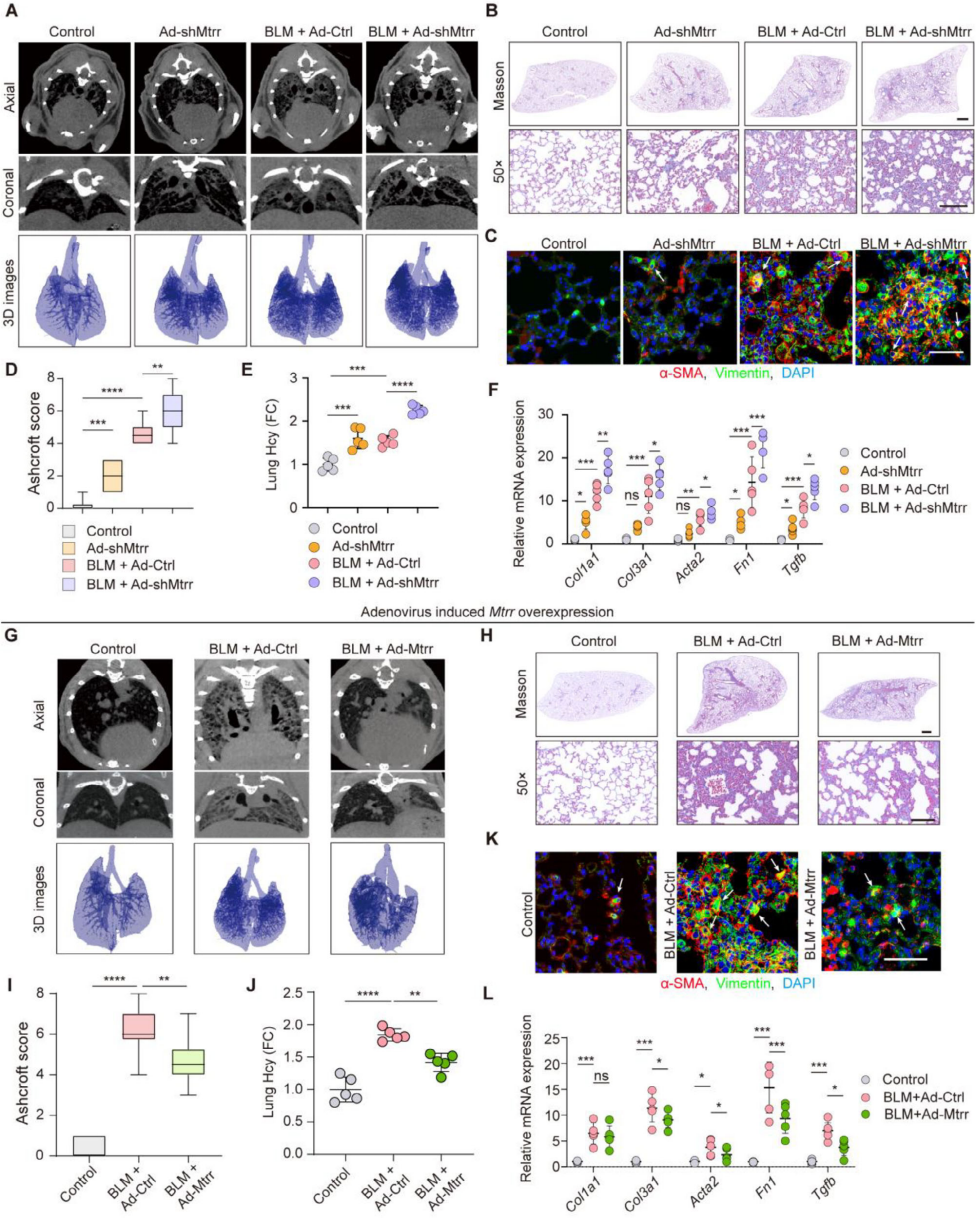
*MTRR* genetic editing impacted IPF progression. A) Representative axial (top row) and their corresponding coronal (bottom row) images from differently treated groups determined by micro‐computed tomography (micro‐CT) showing radiological features. Healthy lungs are black, and lungs with fibrosis were increasingly white (elevated density) (*n* = 5 per group). B) Masson staining of lung sections from different adenovirus treated mice. Images in the lower panels were magnified from the photomicroscopy images in the upper panels (*n* = 5). Upper scale bar = 1 mm; lower scale bar = 50 µm. C) Vimentin costaining with α‐SMA showing fibrosis severity. White arrows showed AT2 cells in EMT process which highly expressed vimentin and α‐SMA. Scale bar = 50 µm. D) Ashcroft score assessed the severity of pulmonary fibrosis (*n* = 10). E) Hcy levels (showed by fold changes) of lung homogenates in different groups were examined by Hcy test kit (*n* = 5 per group). F) Relative mRNA expression of fibrosis related genes (*COL1A1*, *COL3A1*, *ACTA2*, *FN1*, *TGFB*) by RT‐qPCR (*n* = 5 per group). G) Micro‐CT images showing BLM‐induced fibrosis phenotype. H) Masson trichrome staining of left lungs from mice at day 28 post‐BLM (5.0 mg kg^−1^ body weight) or ‐saline (50 µL) administration. I) Lung fibrosis in BLM and Ad‐m*MTRR*, BLM, or control group was assessed by Ashcroft scoring (*n* = 10). J) Fold changes of Hcy level in mouse lung homogenates (*n* = 5 per group). K) Vimentin costaining with α‐SMA showing fibrosis severity. White arrows showed AT2 cells in EMT process which highly expressed vimentin and α‐SMA. Scale bar = 50 µm. L) Relative mRNA expression of genes (*COL1A1*, *COL3A1*, *ACTA2*, *FN1*, *TGFB*) by RT‐qPCR (*n* = 5 per group). Data are presented as the mean ± SEM. *p < 0.05; **p < 0.01; ***p < 0.001; ****p < 0.0001 by one‐way ANOVA with Tukey's multiple comparison tests (D, E, I, and J) and two‐way ANOVA with Tukey's multiple comparison tests (F and L). ns, not significant.

To explore the functional importance of Hcy catabolism in AT2 cells, we focused on *MTRR*, which exhibits the most significant downregulation in IPF. Using adenovirus‐mediated RNA interference (Ad‐sh*MTRR*), we established *MTRR* knockdown models both in vitro and in vivo. In vitro experiments demonstrated that *MTRR* suppression enhanced Hcy accumulation and EMT progression in AT2 cells (Figure S4A–E, Supporting Information). Subsequently, intratracheal administration of Ad‐sh*MTRR* in mice resulted in significant reduction of pulmonary *MTRR* expression compared to Ad‐shCtrl‐treated controls, validating the efficacy of our knockdown approach (Figure S5A–C, Supporting Information). The knockdown resulted in elevated Hcy levels in lung tissue, which were further amplified in the context of BLM‐induced injury (**Figure** **4**E). Micro‐computed tomography (micro‐CT) imaging, 3D reconstruction, survival analysis, and pulmonary function tests revealed characteristic features of pulmonary fibrosis in *MTRR*‐knockdown mice, with these animals displaying enhanced susceptibility to BLM‐induced lung injury compared to controls (Figure 4A). Histological analyses, including hematoxylin and eosin (H&E), IF of fibrosis proteins, and Masson's trichrome staining, demonstrated markedly increased collagen deposition in Ad‐sh*MTRR* lungs (Figure 4B,C; Figure S5D, Supporting Information). The exacerbated fibrotic phenotype in Ad‐sh*MTRR* mice was quantitatively confirmed through Ashcroft scoring, survival analysis and pulmonary function tests (Figure 4D; Figure S5E,G, Supporting Information). Consistently, mRNA levels of fibrosis‐related genes (*COL1A1*, *COL3A1*, *FN1*, *ACTA2*, and *TGFB*) were significantly elevated (Figure 4F). Collectively, these in vivo findings establish a causal relationship between *MTRR* downregulation and Hcy accumulation in promoting pulmonary fibrogenesis.

To evaluate the therapeutic potential of *MTRR*, we investigated whether its overexpression could prevent pulmonary fibrosis in experimental models. In vitro, *MTRR* overexpression reduced Hcy level and EMT in AT2 cells (Figure S4F–J, Supporting Information). Following intratracheal administration of *MTRR*‐overexpressing adenovirus, we confirmed sustained pulmonary expression at 28 days postinjection (Figure S5H–J, Supporting Information). Four weeks post‐BLM administration, *MTRR* overexpression significantly reduced tissue Hcy levels and preserved lung architecture, as evidenced by micro‐CT analysis and 3D reconstruction (Figure 4G). Histological examination through H&E and Masson's trichrome staining demonstrated substantially diminished pulmonary fibrosis in Ad‐*MTRR*‐treated animals (Figure 4H; Figure S5K, Supporting Information), which was further supported by Ashcroft scores and reduced fibrotic markers (Figure 4I; Figure S5L–N, Supporting Information). Moreover, *MTRR* overexpression decreased Hcy level in lung homogenates (Figure 4J) and reduced expression of EMT markers compared to Ad‐Ctrl controls (Figure 4K,L). These results demonstrate that *MTRR* overexpression effectively prevents BLM‐induced lung fibrosis.

Since *CBS* catalyzes the conversion of Hcy to cysteine,^[^ ^20^ ^]^ we investigated whether its overexpression via adenovirus‐encoded *CBS* (Ad‐*CBS*) could reverse lung fibrosis in BLM‐treated mice. Ad‐*CBS* treatment resulted in broad amelioration of fibrosis, reflected by improved micro‐CT imaging, lung function, Masson's trichrome staining, and Ashcroft scores (Figure S6A–G, Supporting Information). Notably, Ad‐*CBS* administration significantly reduced pulmonary Hcy levels in BLM‐induced mice (Figure S6H, Supporting Information). Overexpression of *CBS* markedly suppressed BLM‐induced pulmonary fibrosis, underscoring the contributory role of Hcy in fibrogenesis (Figure S6I–K, Supporting Information). Taken together, these results demonstrate that enhancing Hcy catabolism alleviates pulmonary fibrosis in mice.

### 2.4 Hcy Accumulation Triggered the Onset and Accelerated the Progression of IPF

**Figure 5:**
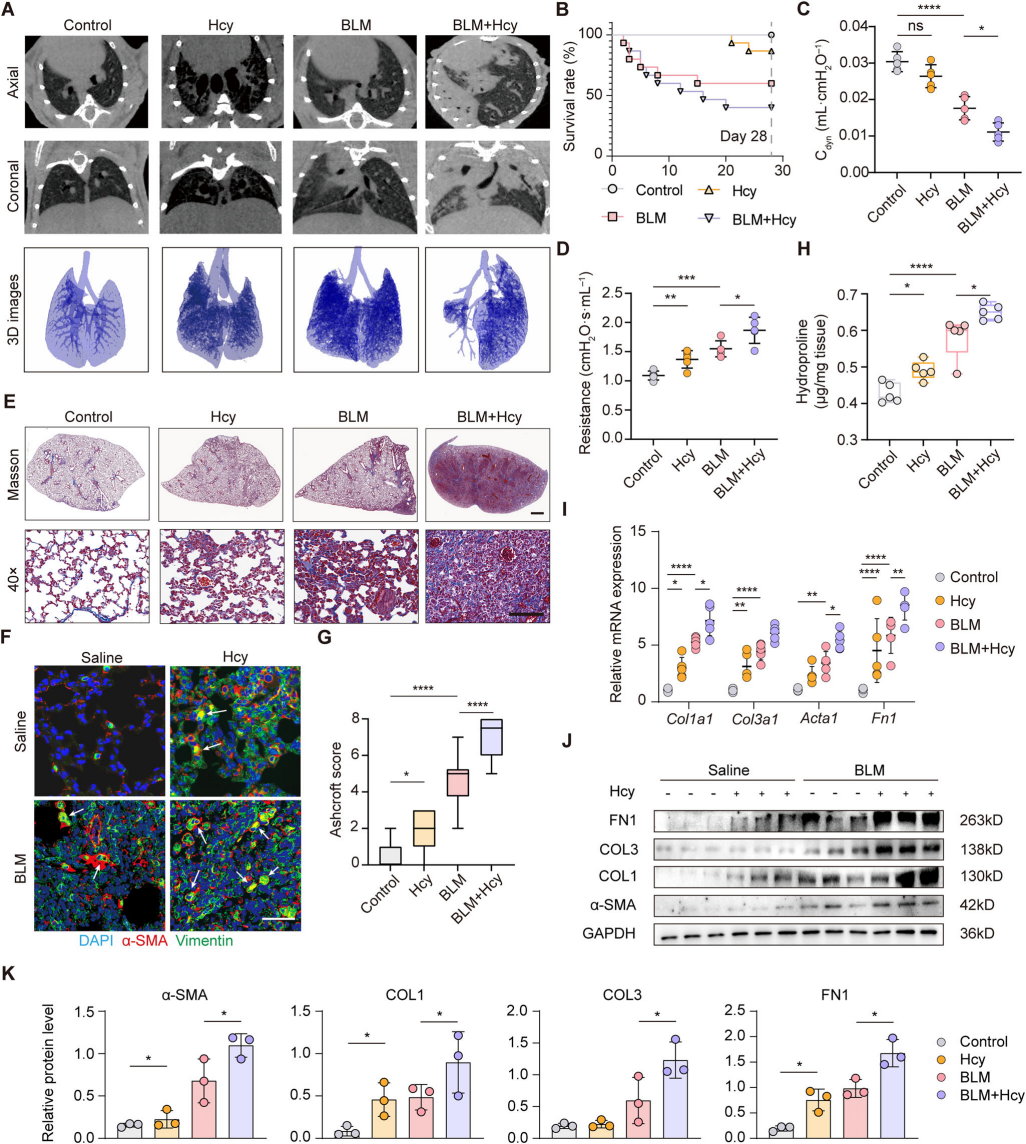
Hcy accelerates IPF progression. A) Axial and their corresponding coronal micro‐CT images were acquired after BLM administration. The lower panels show the representative 3D images drawn out from micro‐CT images, based on different tissues with varying density (*n* = 5). B) Survival curve showing percentage of differently treated mice over a 28 days period experiment (*n* = 15 per group). C,D) Lung function parameters including dynamic compliance (*C*_dyn_) (C) and respiratory resistance (D) among different groups were compared 28 days after BLM challenge (5.0 mg kg^−1^ body weight) (*n* = 5 per group). E) Masson's trichrome staining of lung sections from Hcy‐treated mice in the IPF mouse lung model. Scale bar = 1 mm. Images in the lower panels were enlarged from the photomicroscopy images in the upper panels. Scale bar = 50 µm. F) Immunofluorescence analysis of α‐SMA and vimentin expressions in lung sections. Scale bar = 100 µm. G) Ashcroft scores assessment of lung sections (*n* = 10). H) Hydroxyproline content of lungs from Hcy (100 mg kg^−1^) supplemented or not mice after BLM injury (*n* = 5 per group). I) Quantitative real‐time PCR analysis of *COL1A1*, *COL3A1*, *ACTA1*, and *FN1* mRNA levels in lung homogenates of BLM‐challenged (5.0 mg kg^−1^ body weight) Hcy‐ (100 mg kg^−1^) or saline‐treated mice (*n* = 5). J) Representative western blot results of fibrosis markers (*FN1*, *COL1*, *COL3*, α‐SM(A)). GAPDH was used as the loading control (*n* = 3 per group). K) Relative protein level quantified from western blot results (*n* = 3).Data are presented as the mean ± SEM. *p < 0.05; **p < 0.01; ***p < 0.001; ****p < 0.0001 by log‐rank (Mantel–Cox) test (B), one‐way ANOVA with Tukey's multiple comparison tests (C, D, G, H, and K) and two‐way ANOVA with Tukey's multiple comparison tests (I). ns, not significant.

Building on the linked IPF with dysregulated Hcy metabolism. we sought to determine whether elevated Hcy levels resulting from impaired Hcy metabolism directly contribute to pulmonary fibrosis. In vitro, Hcy treatment increased EMT gene expression in TGF‐β‐treated AT2 cells, although it did not affect cell migration (Figure S7A–G, Supporting Information). For in vivo evaluation, mice received intraperitoneal Hcy (100 mg kg^−1^ every other day for 28 days) significantly elevated serum Hcy levels (Figure S8A, Supporting Information). Histological and immunohistochemical analyses revealed enhanced collagen deposition and α‐SMA expression. These profibrotic effects were significantly exacerbated in mice subjected to BLM challenge, as evidenced by micro‐CT imaging, 3D reconstruction, survival analysis, and pulmonary function tests (**Figure** **5**A–D). Masson's trichrome staining demonstrated substantial collagen accumulation in the lungs of mice treated with both Hcy and BLM, with fibrosis severity confirmed by Ashcroft scoring (Figure 5E,G). Immunofluorescence staining for α‐SMA and vimentin antibodies indicated more pronounced fibrosis in Hcy‐treated mice following BLM administration (Figure 5F; Figure S8D,E, Supporting Information). These mice also showed increased total lung hydroxyproline content (Figure 5H), and a significant correlation (*p* < 0.0001) was observed between Hcy and hydroxyproline levels (Figure S8B, Supporting Information). H&E staining further demonstrated marked alveolar septal thickening and interstitial fibrosis (Figure S8C, Supporting Information). Consistent with these observations, mRNA and protein expression of key fibrotic markers were upregulated in the Hcy and BLM group compared to BLM alone (Figure 5I–K). Together, these results demonstrated that elevated homocysteine levels initiated and accelerated the development of pulmonary fibrosis.

### 2.5 Folate Treatment Alleviates Pulmonary Fibrosis

**Figure 6:**
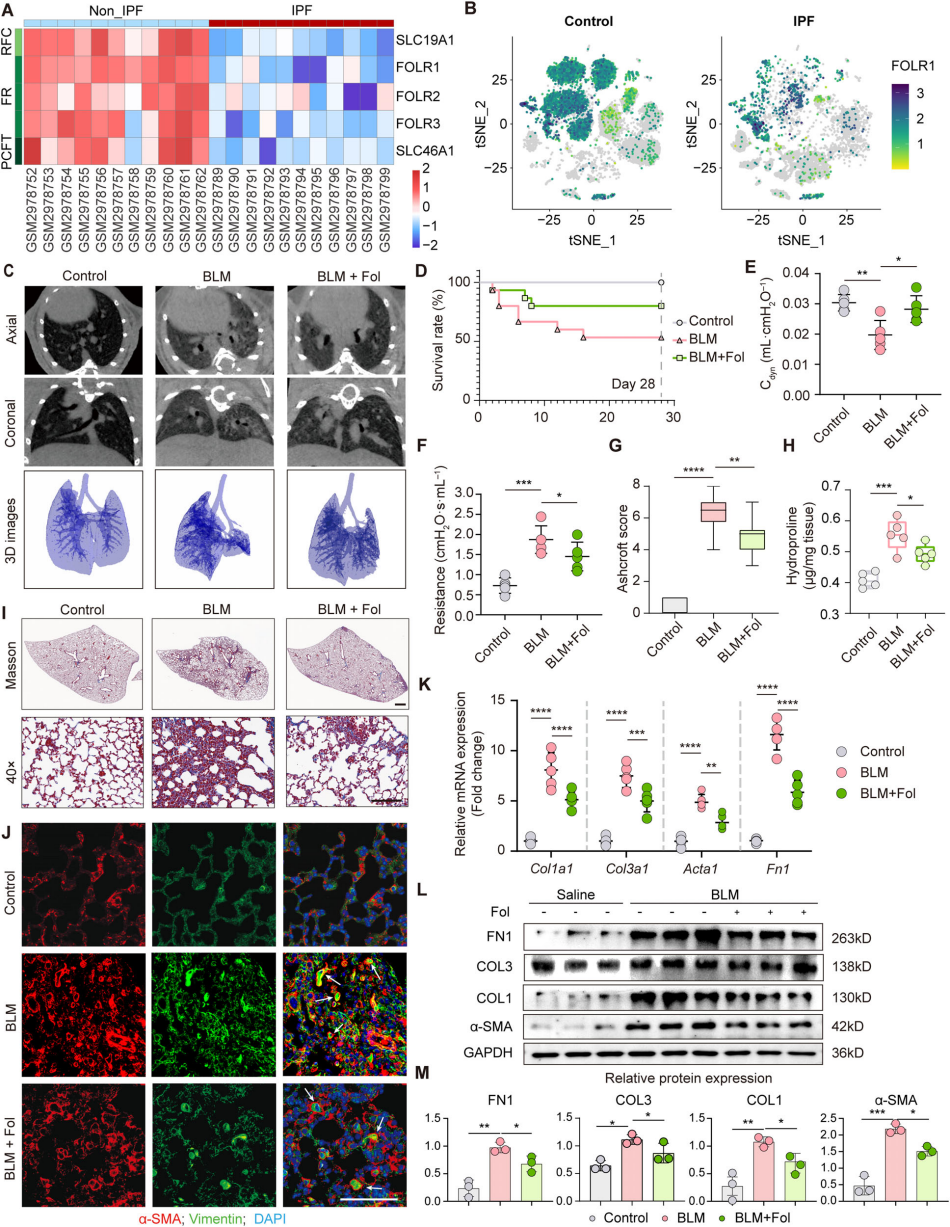
Folate treatment attenuates fibrosis induced by BLM. A) Heatmap of folate transporters showing a significant lower folate transport in IPF lung tissues (data source: GSE110147). B) Single‐cell analysis indicating AT2 cells played the main role in folate transport and exhibited an inhibited transport of folate in IPF samples. C) Representative axial, coronal, and 3D reconstruction images determined by micro‐CT showing interstitial pathological changes. D) Percentages of surviving mice were plotted over a 28 days period post‐BLM (5.0 mg kg^−1^ body weight) or Fol (0.5 mg kg^−1^) administration (*n* = 15 at start). E,F) Lung function parameters including dynamic compliance (*C*_dyn_) (E) and respiratory resistance (F) among different groups were compared 28 days after BLM (5.0 mg kg^−1^ body weight) challenge or Fol treatment (0.5 mg kg^−1^) (*n* = 5). G) Ashcroft score quantified fibrosis level of lung sections (*n* = 10). H) Hydroxyproline content of lungs from Fol‐ (0.5 mg kg^−1^) or saline‐treated mice after BLM injury (5.0 mg kg^−1^ body weight) (*n* = 5 per group). I) Masson trichrome staining of left lungs from Fol‐treated or not treated mice at day 28 post‐BLM or ‐saline administration. Scale bar = 1 mm. Images in the lower panels were magnified from the photomicroscopy images in the upper panels. Scale bar = 50 µm. J) Immunofluorescence analysis showing α‐SMA and vimentin positive AT2 cells in lung sections (indicated by white arrows). Scale bar = 100 µm. K) Fibrosis marker genes relative expression level was showed by RT‐PCR (*n* = 5). L,M) Representative western blots analyzing fibrosis proteins (*FN1*, *COL1*, *COL3*, α‐SMA) in lung samples and their densitometric values normalized to GAPDH (*n* = 3 per group). Data are presented as the mean ± SEM. *p < 0.05; **p < 0.01; ***p < 0.001; ****p < 0.0001 by log‐rank (Mantel–Cox) test (D), one‐way ANOVA with Tukey's multiple comparison tests (E–H, and M) and two‐way ANOVA with Tukey's multiple comparison tests (K). ns, not significant.

Previous studies have demonstrated that Hcy accumulation primarily results from insufficient dietary Fol intake.^[^ ^21^ ^]^ Using bulk‐RNA‐seq and scRNA‐seq, we detected reduced expression of folate‐transport‐related genes, specifically the folate receptor alpha (*FOLR1*), in IPF tissue (**Figure** **6**A,B). Analysis of clinical data further revealed significantly lower plasma folate levels in IPF patients compared to controls (6.2 ± 0.8 vs 11.5 ± 1.2 ng mL^−1^), as well as reduced folate concentrations in BALF (1.4 ± 0.3 vs 2.9 ± 0.4 ng mL^−1^). Critically, plasma Fol inversely correlated with plasma Hcy (*r* = −0.6051), consistent with the folate‐dependent remethylation of Hcy (Figure S9A–C, Supporting Information). To investigate whether Fol supplementation could promote Hcy catabolism and reverse pulmonary fibrosis, we conducted both in vitro and in vivo experiments. In TGF‐β1‐treated AT2 cells, we used tetrahydrofolate (THF) instead of folate as epithelial cells poorly utilize exogenous Fol directly. THF treatment effectively decreased TGF‐β1‐induced cell migration rates (Figure S10A,B, Supporting Information) and attenuated EMT, as indicated by reduced vimentin^+^/α‐SMA^+^ double‐positive cell in AT2 cells (Figure S10C,D, Supporting Information). Furthermore, quantitative polymerase chain reaction (qPCR) and western blot analyses revealed that THF significantly suppressed TGF‐β1‐induced expression of EMT‐related genes (Figure S10E,F, Supporting Information).

To evaluate the therapeutic potential of Fol in reversing pulmonary fibrosis in vivo, Fol was administered via oral gavage for 28 days following intratracheal BLM instillation. Although micro‐CT imaging showed no significant intergroup differences, 3D reconstruction revealed that Fol treatment substantially restored lung architecture compromised by BLM injury (Figure 6C). Fol supplementation also significantly improved survival rates among BLM‐challenged mice (Figure 6D) and ameliorated airway hyperresponsiveness and resistance (Figure 6E,F). Histological examination through H&E and Masson staining indicated reduced collagen deposition in folate‐treated BLM mice (Figure 6I; Figure S11A, Supporting Information). Fol administration notably lowered Ashcroft scores, hydroxyproline content, and Hcy accumulation in lung tissues (Figure 6G,H; Figure S11B,C, Supporting Information). Immunostaining for α‐SMA and vimentin indicated attenuated fibrotic pathology following Fol treatment (Figure 6J; Figure S11D,E, Supporting Information). Additionally, qPCR and western blot demonstrated that Fol treatment significantly attenuated BLM‐induced expression of fibrosis‐related genes (Figure 6K–M). Collectively, these results indicate that Fol effectively reduces Hcy levels and ameliorates pathological progression in pulmonary fibrosis.

### 2.6 Fol Ameliorates STX17 Hcy‐Lation and Ubiquitination in IPF

**Figure 7:**
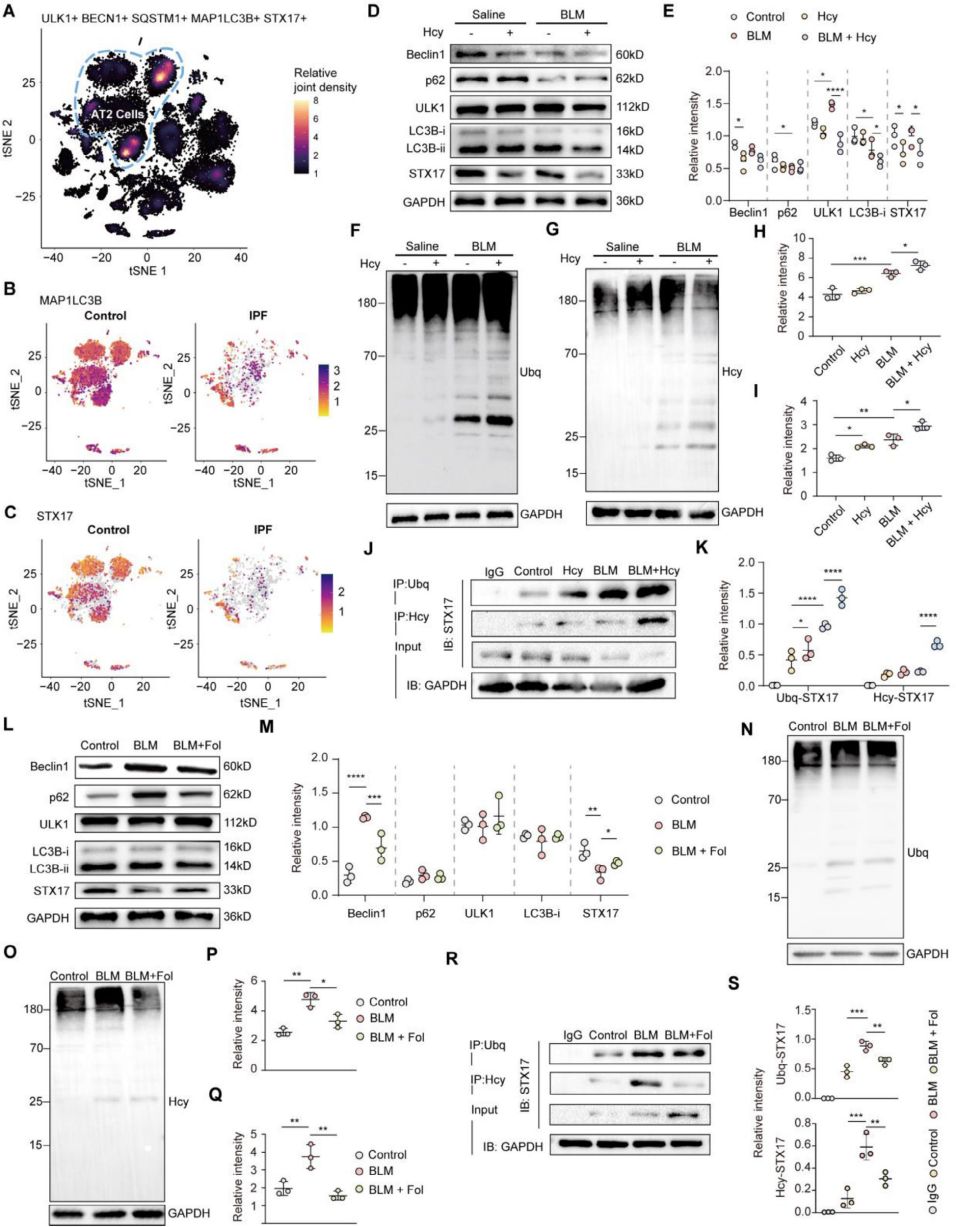
STX17 is downregulated via homocysteinylation and ubiquitination in IPF. A) Joint density plot of key autophagy genes created by scCustomize R package. B,C) Feature plots showing differences of MAP1LC3B (B) and STX17 (C) expression between control and IPF group. D,E) Representative Western blots analyzing autophagy proteins (Beclin1, p62, ULK1, LC3B) and SNARE protein component STX17 (D) and their densitometric values normalized to GAPDH (E) (*n* = 3). F,G) Representative western blot analyzing protein ubiquitination and homocysteinylation in lung tissues. H) Protein ubiquitination densitometric values normalized to GAPDH (*n* = 3). I) Protein homocysteinylation densitometric values normalized to GAPDH (*n* = 3). J) Immunoprecipitation of ubiquitinated and Hcy‐lated proteins and detection of STX17 protein levels by western blotting in the lung tissues. K) Ubiquitinated STX17 (STX17‐Ubq) and Hcy‐lated (STX17‐Hcy) densitometric values normalized to STX17 protein level in input samples. L,M) Representative Western blots analyzing autophagy proteins (Beclin1, p62, ULK1, LC3B) and SNARE protein component STX17 from Folate‐treated (0.5 mg kg^−1^) or not treated BLM (5.0 mg kg^−1^ body weight) challenged samples (L) and their densitometric values normalized to GAPDH (M) (*n* = 3). N,O) Representative western blot analyzing protein Ubiquitination and homocysteinylation in lung tissues. P) Protein ubiquitination densitometric values normalized to GAPDH (*n* = 3). Q) Protein homocysteinylation densitometric values normalized to GAPDH (*n* = 3). R) Immunoprecipitation of Ubq and Hcy and STX17 detection by Western blotting using lung tissue lysates as inputs. Representative Western blots are demonstrated. S) Ubiquitinated STX17 (STX17‐Ubq) and Hcy‐lated (STX17‐Hcy) densitometric values normalized to STX17 protein level in input (*n* = 3). Data are presented as the mean ± SEM. *p < 0.05; **p < 0.01; ***p < 0.001; ****p < 0.0001 by one‐way ANOVA with Tukey's multiple comparison tests (H, I, K, P, Q, and S) and two‐way ANOVA with Tukey's multiple comparison tests (E and M). ns, not significant.

Impaired autophagic pathways have previously been reported in lung epithelial cells of IPF patients.^[^ ^22^, ^23^ ^]^ Our scRNA‐seq analysis further revealed significant decreases in autophagy‐related proteins, including ULK1, Beclin1, LC3B, and STX17, specifically in AT2 cells of IPF patients (**Figure** **7**A–C; Figure S12A–D, Supporting Information). STX17, a crucial component of the SNARE complex mediating autophagosome–lysosome fusion, plays a vital role in autophagy regulation. To explore the connection between Hcy and autophagy impairment, we examined autophagy protein expression, including STX17, in pulmonary tissue from mice treated with Hcy and BLM, observing significant reductions in these proteins (Figure 7D,E; Figure S13A–F, Supporting Information). Consistent with observations in murine fibrotic lungs, immunofluorescence analysis revealed significantly reduced STX17 expression in AT2 cells of human IPF tissues compared to controls (Figure S14A,B, Supporting Information), underscoring the clinical relevance of STX17 depletion in IPF pathogenesis. Previous research by Tripathi et al. established that Hcy induces STX17 homocysteinylation and ubiquitination, leading to its degradation and subsequent autophagy blockade.^[^ ^13^ ^]^ To verify this mechanism, we assessed ubiquitination levels in pulmonary tissue from Hcy‐ and BLM‐treated mice via western blots. The results showed increased ubiquitination that correlated with both Hcy levels and fibrosis severity (Figure 7F,H). Since homocysteinylated proteins are known targets for ubiquitination and proteasomal degradation, we evaluated homocysteinylation levels and observed a notable increase in lung tissues from Hcy‐ and BLM‐treated mice, which coincided with elevated ubiquitination (Figure 7F–I). Western blot analysis revealed a modified protein band slightly above 25 kDa, corresponding to the molecular weight of STX17 (≈33 kDa). Co‐IP assays further confirmed enhanced ubiquitination and homocysteinylation of STX17 following Hcy and BLM exposure. These posttranslational modifications occurred concomitantly with a reduction in total STX17 protein (Figure 7J,K), supporting the notion that STX17 degradation in IPF is mediated through homocysteinylation‐driven ubiquitination.

We next evaluated whether Fol treatment could modulate autophagy in IPF. Expression analysis of autophagy‐related proteins in lung tissues from BLM‐induced mice revealed that Fol supplementation significantly restored STX17 levels (Figure 7L,M; Figure S13C,D, Supporting Information). Moreover, Fol administration reduced both global homocysteinylation and ubiquitination levels in the lungs of BLM‐injured mice (Figure 7N–Q). To further elucidate the specific effects of Fol therapy on STX17 modifications during IPF progression, we performed immunoprecipitation of STX17 from lung tissues of mice that received Fol treatment throughout the BLM induction. Notably, we observed that Fol treatment simultaneously decreased homocysteinylated and ubiquitinated STX17 levels (Figure 7R,S). These findings collectively suggest that Fol ameliorates IPF‐associated autophagy dysfunction by reducing STX17 homocysteinylation, thereby preventing STX17 degradation and restoring autophagy flux in fibrotic lungs.

## 3 Discussion

IPF is a devastating interstitial lung disease characterized by dysregulated inflammation and progressive lung scarring, ultimately leading to death from respiratory complications.^[^ ^24^ ^]^ While current therapeutic agents, nintedanib and pirfenidone, can significantly delay the decline in lung function, no existing treatments can completely halt disease progression, highlighting the urgent need for novel therapeutic interventions.^[^ ^25^ ^]^ Our investigation revealed Hcy levels was elevated in plasma and BALF of IPF patients. Through scRNA‐seq analysis, we identified a downregulation of Hcy catabolism genes specifically in AT2 cells. Further experimental evidence demonstrated that either Hcy supplementation or suppression of Hcy catabolism through *MTRR* knockdown promoted IPF onset and progression by enhancing STX17 homocysteinylation. Conversely, promoting Hcy catabolism, either through *MTRR* overexpression or folate administration, reduced STX17 homocysteinylation and ameliorated pulmonary fibrosis. Therefore, enhancement of Hcy metabolism may contribute to ameliorate pulmonary fibrotic effects resulted from elevated Hcy levels and Fol supplementation is a suitable choice.

Hcy is a well‐established risk factor for various pathological conditions, including cardiovascular diseases and neurodegenerative disorders, primarily due to its capacity to induce oxidative stress, trigger inflammation, impair vascular function, and disrupt cellular processes.^[^ ^26^ ^]^ It is also a potential reason for several other diseases. In this study, we identified elevated local Hcy levels as a significant risk factor for IPF. Through both Hcy supplementation and *MTRR* knockdown experiments, we demonstrated that HHcy promotes pulmonary fibrosis. Fol, a crucial substrate for *MTHFR*, plays a central role in the folate cycle and transmethylation processes that facilitate the conversion of Hcy to methionine (Met). The modulation of basal plasma Hcy concentration is Fol‐dependent, Fol supplementation has been established as a standard therapeutic approach for HHcy. Notably, folate administration effectively reduced Hcy levels in lung tissue and ameliorated pulmonary fibrosis. Our findings align with previous research documenting Hcy‐induced respiratory system damage. A research in microbe revealed chronic airway carriage of *Staphylococcus aureus* increased airway homocysteine, which activated host AKT1‐S100A8/A9 axis to start inflammation and tissue injury in lung.^[^ ^27^ ^]^ Additionally, another research reported exacerbated pulmonary injury after cigarette smoke exposure in HHcy mice.^[^ ^28^ ^]^ Accordingly, our findings showed that higher Hcy levels enhanced fibrosis and pulmonary injury.

Disorders in Hcy metabolism represent one of the primary causes of elevated Hcy level.^[^ ^29^ ^]^ In this metabolic pathway, Hcy is generated through Met during methyl group donation reactions. The reconversion of Hcy to Met, catalyzed by *MTRR*, requires an efficient folate cycle that provides necessary folate derivatives. Consequently, deficiencies in enzymes involved in either the folate cycle or Hcy metabolism pathways significantly contribute to HHcy development.^[^ ^30^ ^]^ Earlier researches have confirmed a common C677T mutation in the *MTHFR* gene results in lower enzyme activity, and contributes to increased Hcy levels and decreased Fol levels.^[^ ^31^, ^32^ ^]^ Other polymorphisms of Hcy metabolic enzymes are also under researches, such as *MTRR* A66G polymorphism and 844ins68 of *CBS*.^[^ ^33^, ^34^ ^]^ Through bulk RNA sequencing analysis, we observed decreased expression of key Hcy metabolism genes *MTRR* in IPF patients. To further elucidate the cell‐specific expression patterns of these Hcy metabolism genes during pulmonary fibrotic processes, we conducted single‐cell RNA sequencing analysis of IPF patient samples. Noticeably, Hcy or Fol metabolism disruption in IPF is not a global effect. A significant finding from our study is that AT2 cells function the most in Hcy metabolism, whose amount and metabolic ability strikingly drops in pathological environment of IPF. Our findings revealed that Hcy metabolism genes are predominantly expressed in AT2 cells, which exhibit markedly reduced numbers and metabolic capacity in the pathological environment of IPF. In addition, we also found folate‐transport‐related genes *SLC19A1* and *FOLR1* showed decreased expression in IPF patients, suggesting reduced folate uptake in these individuals. While fibroblasts are known to play a crucial role in fibrosis, emerging evidence suggests that epithelial cell dysfunction represents a central component in IPF pathophysiology.^[^ ^35^ ^]^ It has been proved that AT2 cells targeted injury or depletion firmly induces pulmonary fibrosis.^[^ ^36^, ^37^ ^]^ Even senescence of AT2 cells drives progressive pulmonary fibrosis.^[^ ^3^ ^]^ Moreover, recent transcriptomic analysis of IPF samples has revealed distinct metabolic reprogramming in AT2 cells.^[^ ^38^, ^39^ ^]^ Our results substantiate these findings by demonstrating impaired Hcy metabolism and folate‐transport‐related genes in AT2 cells, potentially resulting in local Hcy accumulation.

In our study, we observed that Hcy accumulation and *MTRR* downregulation specifically within AT2 cells correlate with increased expression of EMT markers and a more severe fibrotic phenotype. Folate treatment mitigated these EMT marker changes alongside fibrosis. Rock et al. demonstrated that collagen‐producing fibroblasts in bleomycin‐induced fibrosis did not originate from AT2.^[^ ^40^ ^]^ Our findings regarding increased expression of mesenchymal markers in AT2 cells under fibrotic conditions align with observations of a transitional or “EMT‐like” state rather than implying complete conversion into functional fibroblasts. This phenotypic shift in AT2 cells—increasingly recognized as a complex phenomenon in IPF pathogenesis—potentially contributes to fibrosis through mechanisms distinct from generating matrix‐producing fibroblasts. AT2 cells may undergo a partial EMT/activated state, acquiring mesenchymal features, including enhanced migratory/invasive potential, without full commitment to a fibroblast fate; this state promotes fibrosis by enhancing profibrotic signaling (e.g., TGF‐β secretion), impairing reepithelialization, and facilitating stromal interactions.^[^ ^3^, ^41^, ^42^ ^]^ Furthermore, EMT‐like changes frequently associate with senescence and SASP, wherein senescent AT2 cells secrete inflammatory mediators that activate fibroblasts and drive chronic inflammation.^[^ ^3^, ^43^ ^]^ Critically, the acquisition of mesenchymal markers signifies dysfunction and failed repair, marked by loss of alveolar epithelial identity and impaired regenerative capacity, thereby creating a permissive environment for fibroblast activation and persistence.^[^ ^35^, ^44^ ^]^

Previous research has demonstrated that progressive increases in hepatic protein homocysteinylation and ubiquitination of STX17 are associated with autophagy blockade and subsequent fibrosis in nonalcoholic fatty liver disease.^[^ ^13^ ^]^ Consistent with these findings, our results revealed that elevated Hcy levels enhanced homocysteinylation and ubiquitination of STX17. Notably, Fol administration reversed these excessive modifications, thereby restoring autophagy function and inhibiting IPF progression. The observed elevation in homocysteinylation and ubiquitination indicates disrupted proteostasis (a network of processes handling protein folding and degradation composed of a complicated set of cellular systems and subcellular compartments), leading to functional impairments including compromised autophagy. Autophagy serves not only as a cellular response to nutrient deprivation but also as a crucial mechanism for cellular quality control, particularly in alleviating stress on the ubiquitin–proteasome system.^[^ ^45^ ^]^ Multiple studies have demonstrated the effect of autophagy in IPF. On the one hand, impaired autophagy aggravates IPF progression.^[^ ^16^, ^46^ ^]^ On the other hand, activated autophagy attenuates pulmonary fibrosis and inflammation.^[^ ^47^, ^48^, ^49^ ^]^ Our single‐cell analysis identified AT2 cells as the primary autophagy executors among lung cells. In IPF conditions, AT2 cells exhibit increased homocysteinylation and ubiquitination of STX17, a SNARE protein essential for autophagosome–lysosome fusion.^[^ ^50^ ^]^ These posttranslational modifications result in decreased STX17 expression, consequently blocking downstream autophagy processes. This suggests a mechanistic pathway wherein elevated Hcy levels trigger STX17 modifications, leading to impaired autophagy flux. The resulting dysfunction creates a self‐perpetuating cycle, compromising both proteostasis and Hcy metabolism in AT2 cells. This vicious cycle ultimately diminishes the lung tissue's capacity to metabolize Hcy and accelerates IPF progression.

Our study provides novel insights into the role of Hcy in IPF by focusing on AT2 cells and the homocysteinylation of STX17. However, several limitations should be acknowledged. First, while we have concentrated on AT2 cells, the increase in plasma Hcy levels may also affect other cell types, such as fibroblasts, which are crucial in fibrosis development. Consequently, the potential impact of Hcy on fibroblasts and other cell types warrants further investigation. Second, although we identified elevated homocysteinylation of STX17 as a critical mechanism underlying autophagy impairment in IPF, it is plausible that other proteins or genes are similarly affected by Hcy modifications. The ubiquitin–proteasome system and other SNARE proteins, such as VAMP7/8 and SNAP29, involved in autophagosome–lysosome fusion, could also be potential targets of Hcy modifications. Future studies should explore the broader effects of Hcy on the autophagy pathway and identify additional targets of homocysteinylation in IPF.

Taken together, our study revealed the potential risk of higher Hcy level on IPF severity. Specifically, the pathological process of pulmonary fibrosis is primarily mediated through dysfunction of AT2 cells, characterized by compromised Hcy metabolism and impaired autophagy mechanisms. Considering that Fol has been designated as a dietary supplement by the FDA and maintains a well‐established safety profile, it presents a promising therapeutic approach for the prevention and treatment of IPF.

## 4 Experimental Section

### Study Design

In this study, it was aimed to investigate whether Hcy promoted IPF and the probable mechanism. To accomplish that, clinical data of IPF patients (lung function testing reports and laboratory biochemical sheets) and biopsy specimens were first retrospected. Inclusion criteria of IPF was followed by clinical diagnosis from experts in respiratory medicine according to the respective diagnostic criteria for this disease (ATS/ERS, 2018).^[^ ^51^ ^]^ Multiple mouse models were then conducted after BLM treatment. Experiments were conducted in a randomized and blinded manner. 8 weeks old male C57BL/6J mice involved in individual groups, consisting of at least five mice per group. Three biological replicates and three technical replicates were set as in vitro experiments standard. All animal experiments were approved by the Tongji University School of Medicine and the Animal Care and Ethics Committees of Tongji University School of Medicine. Human clinical data were collected under Kunming Medical University approval S2020‐173‐01.

### Human Subjects

Human IPF lung tissue was obtained from patients undergoing surgery for organ transplantation in First Affiliated Hospital of Kunming Medical University. Healthy control samples were obtained from non‐IPF histology lung samples from patients with lung cancer in the hospital. All the human bronchoalveolar lavage fluid samples from patients with IPF and control subjects were obtained from the Biobank at the First Affiliated Hospital of Kunming Medical University. IPF was diagnosed by chest high resolution CT images and other examinations according to the respective diagnostic criteria for this disease (ATS/ERS, 2018).^[^ ^51^ ^]^ Informed written consent was obtained from all participants. The research was approved by the Institutional Review Board at First Affiliated Hospital of Kunming Medical University. All human sample studies were performed in compliance with the official ethical regulations.

### General Mouse Care and Study Approval

Male C57BL/6J (wild type) mice were obtained from the Shanghai Animal Center and randomized into experimental groups. Mice were housed under ambient temperature of 24 ± 2 °C, circulating air, constant humidity of 50 ± 10%, and a 12:12 h light/dark cycle. The animal sample size for each experiment was selected based on previous, well‐characterized studies. The number of animals in each group was specified in figure legends. All experiments using mice were approved by the Tongji University School of Medicine and the Animal Care and Ethics Committees of Tongji University School of Medicine. All relevant ethical regulations were complied with and approved study protocols were used.

### Experimental IPF in Mice

Experimental IPF was induced by intratracheally instilled bleomycin in 8 weeks old male mice. 8 weeks old male C57BL/6J mice were intratracheally instilled with 50 µL saline or BLM at a dose of 5.0 mg kg^−1^ body weight diluted in 50 µL saline to set up the control or IPF model on day 0. For Fol treatment, daily irrigation of Fol (0.5 mg kg^−1^) was conducted. The dosage used was FDA approved human equivalent dose. At day 28, mice were sacrificed, the blood sample and lung tissues were collected for further analysis. Left lung was fixed in 10% buffered formalin for 48 h for histological analysis.

For HHcy mouse model, daily Hcy (H4628, Sigma‐Aldrich) intraperitoneal injection at a dosage of 100 mg kg^−1^ was used on 12 weeks old male C57Bl/6J mice. After 28 days of injection, mice were treated as the protocol in bleomycin‐induced IPF model. Lung specific *MTRR* knockdown mice were generated by injecting Ad‐sh*MTRR* (1.0 × 10^9^ U per mice) (Viraltherapy Technologies) intratracheally at days 0 and 10. To avoid the injury caused by repetitive invasive operation, BLM intraperitoneal injection (40 mg kg^−1^) was used to induce IPF model, which was conducted twice a week for weeks. *MTRR* overexpression was also completed by Ad‐m*MTRR* injection. At day 21, mice were sacrificed, the blood sample and lung tissues were collected for further analysis. *CBS* overexpression was as the same procedures, using adenovirus‐mediated gene delivery of Ad‐*CBS*.

### Cell Culture

Mice were anesthetized using isoflurane via intraperitoneal injection. Once anesthesia was confirmed, a thoracotomy was performed, and the lungs were carefully excised and washed with cold phosphate‐buffered saline (PBS) to remove any residual blood. The excised lungs were minced into 1–2 mm pieces and transferred to a 50 mL conical tube containing digestion buffer, which included PBS, 0.5 mg mL^−1^ collagenase D, 0.5 mg mL^−1^ dispase II, and 25 µg mL^−1^ DNase I. The tissue was incubated in a 37 °C water bath with gentle shaking for 30–45 min, with agitation every 10 min to ensure complete digestion. After digestion, the cell suspension was filtered through a 70 µm cell strainer to remove undigested tissue. The resulting suspension was centrifuged at 300–400 *g* for 10–15 min at 4 °C, and the pellet was resuspended in RPMI‐1640 medium with 10% FBS. A Percoll density gradient (30%, 40%, and 70% solutions) was prepared in a 50 mL conical tube, and the cell suspension was layered on top. After centrifugation at 800 *g* for 20–30 min at 4 °C without braking, the type II alveolar epithelial cells (AECII)‐enriched fraction, located between the 40% and 70% layers, was carefully collected. The cells were then resuspended in culture medium and counted, with cell viability assessed using a trypan blue exclusion assay, ensuring ≥90% viability for further experiments. To confirm the purity of AECII, flow cytometry was performed using primary antibodies specific for SP‐A, SP‐B, and SP‐C. For culture, AECII were maintained in RPMI‐1640 medium with 10% FBS at 37 °C in a 5% CO_2_ incubator, with media changes every 2–3 days.

To build cellular fibrosis model, cells were treated with 10 ng mL^−1^ TGF‐β. To investigate the effect of Hcy, cells were treated with 200 nm Hcy. 50 µm tetrahydrofolate (T3125, Sigma‐Aldrich), the main internal type of Fol, was used to investigate its cellular effect.

### Histopathology of Mice Lung Sections

Lung tissue sections were prepared for histological examination using both H&E and Masson's trichrome staining. For H&E staining, tissues were fixed in 10% neutral buffered formalin, paraffin‐embedded, sectioned at 4 µm, and stained with hematoxylin followed by eosin to visualize nuclei and cytoplasm, respectively. Masson's trichrome staining was employed to assess collagen fiber deposition, with tissues first stained with Wiegert's hematoxylin, then with Biebrich scarlet‐acid fuchsin to highlight collagen, and finally with aniline blue to differentiate muscle and connective tissues. Each staining step was followed by appropriate washing, differentiation, and dehydration processes before mounting with a synthetic medium. Slides were examined under a light microscope to evaluate tissue morphology and fibrosis.

### Immunofluorescence and Immunohistochemical Staining

The lungs from mice were insufflated with a mix of 1:1 water/OCT and quickly frozen at −80 °C. Slides from those lungs were fixed in methanol for 15 min before being saturated in 5% FBS. Primary antibodies for Vimentin, α‐SMA, *MTRR*, and STX17 were incubated overnight at 4 °C. Goat anti‐rabbit and goat anti‐mouse conjugated with Alexa Fluor 594 or Alexa Fluor 488 (Molecular Probe, InVitrogen, Cergy Pontoise, France) were used as secondary antibodies at a dilution of 1: 2000.

Lung Tissues were dehydrated and embedded in paraffin. For histological examination, 4 µm thick tissue sections on slides were treated with 1.4% H_2_O_2_–methanol for 30 min to block endogenous peroxidase. Then, nonspecific binding was blocked with 1.5% normal serum, and the slides were incubated with rabbit anti‐STX17 polyclonal antibodies (1:500; 81899‐1‐RR, Proteintech). The sections were incubated with SABC‐HRP Kit (P0603, Beyotime). The color reaction was developed by incubation with a 3,3′‐diaminobenzidine liquid substrate system (D3939, Sigma‐Aldrich). The slides were counterstained with Harris's hematoxylin for 1 min after immunohistochemical staining.

### Homocysteine Measurement

Homocysteine concentration was detected with Total HCY enzyme‐linked immunosorbent assay (ELISA) kit (EU20003, FineTest) following the manufacturer's protocol. Briefly, cell lysates were prepared by centrifuging suspension cells at 2500 rpm, 2–8 °C for 5 min, resuspending in cold PBS, then adding 0.5–1 mL lysis buffer with protease inhibitors and lysing on ice or sonicating. For adherent cells, medium was aspirated, cells washed with cold PBS, lysed with buffer after scraping. Lysates were centrifuged at 5000× *g* for 5 min and supernatant was collected. Homocysteine was measured with the ELISA kit. Reagents were brought to room temperature and diluted as needed. 50 µL supernatant was added to wells, incubated at 37 °C for 60 min. Plate was washed, 50 µL HRP–Streptavidin added, and incubated at 37 °C for 30 min. After washing, 50 µL TMB substrate was added, incubated in dark at 37 °C for 15–30 min, then stopped. Absorbance was measured at 450 nm. Homocysteine concentration was determined from a standard curve.

### Hydroxyproline Content Measurement in Lung Tissues

Tissue hydroxyproline content was measured using Hydroxyproline Assay kit (Colorimetric) (ab222941, Abcam). 10 n concentrated NaOH was added to the samples and hydrolyze at 120 °C for 1 h. Then, samples were neutralized with 10 n concentrated HCl after cooling to the room temperature, followed by centrifugation to collect the supernatant. Both the samples and standards were added to the wells and the wells were heated at 65 °C to evaporate them to dryness. After that, the oxidation reagent mix was added to dissolve any crystalline residue and incubated at room temperature for 20 min. The developer was added and incubated at 37 °C for 5 min. Then, the DMAB concentrate was added and incubated for 45 min at 65 °C. Results were measured using a microplate reader (Infinite F50, TECAN).

### Ashcroft Scoring

Ashcroft scoring system was used to estimate the severity of pulmonary fibrosis. Criteria for grading lung fibrosis were as follows. 0 point: normal lung; 1 point: minimal fibrous thickening of alveolar or bronchiolar walls; 3 points: moderate thickening of walls without obvious damage to lung architecture; 5 points: increased fibrosis with definite damage to lung structure and formation of fibrous bands or small fibrous masses; 7 points: severe distortion of structure and large fibrous areas; “honeycomb lung” is placed in this category; 8 points: total fibrous obliteration of the field. Scores of 2, 4, and 6 represented intermediate stages between the respective points.

### Micro‐CT Analysis

Mice were anesthetized and transferred into an apparatus to scan the area of the lung fields on day 28. High‐resolution scans were obtained using the LCT‐200 micro‐CT Imaging system (UNITED WELL, Shanghai, China) or the Super Nova CT (PINGSENG Healthcare Inc., SNC‐100, Kunshan, China) according to the manufacturer's instructions.

### Mice Lung Function Test

Lung function parameters such as dynamic compliance and respiratory resistance were detected and determined using the Pulmonary Function Test System (Buxco Research Systems, USA) according to the manufacturer's protocol. Briefly, mice were first anesthetized with Avertin solution (T48402, Sigma‐Aldrich), and transferred into the body chamber of the detecting system.

### Wound Healing Assay

Wound healing assays were performed to evaluate cell migration (to characterize epithelial–mesenchymal transformation process) using AT2 cells cultured to confluence in 24‐well plates. Prior to the assay, cells were subjected to serum starvation in medium containing 0.5% FBS for 24 h. Using sterile 200 µL pipette tips, linear wounds were created in the confluent cell monolayer. The wounded cell layers were then gently washed with PBS to remove cellular debris before being incubated in complete culture medium, either with or without TGF‐β (2 ng mL^−1^), at 37 °C in a 5% CO_2_ incubator. The wound healing process was monitored for 48 h postscratching. Wound images were captured using an Olympus CKX41 microscope equipped with an Olympus SC30 camera and analyzed using cellSens Entry software. Quantitative analysis of wound closure was performed using the wound healing tool in ImageJ, with results expressed as percentages relative to the initial wound area.

### RNA Isolation and Quantitative Real‐Time Polymerase Chain Reaction (qRT‐PCR)

Total RNA from cells or tissues was extracted using TRIzol reagent (R401, Vazyme) for qPCR, which was subsequently reverse‐transcribed into cDNA with HiScript II Q RT SuperMix (R222‐01, Vazyme). An Applied Biosystems 7500 device with ChamQ SYBR qPCR Master Mix (Q331‐02, Vazyme) was used to conduct qRT‐PCR consequently. Expression data were normalized to β‐actin mRNA expression and fold‐change was calculated using 2^−∆∆Ct^ approach.

### Western Blot Analysis

Cells were implanted into six‐well plates and cultured until attachment. RIPA lysis buffer (P0013B, Beyotime) mixed with protease and phosphatase inhibitor cocktail for mammalian cell and tissue extracts (P1050, Beyotime) was used in sample protein extraction. The protein concentration was then determined by the Pierce BCA protein assay kit (A55864, Thermo Fisher Scientific). The collected proteins were separated via sodium dodecyl sulfate–polyacrylamide gel electrophoresis procedure and subsequently electrotransferred to polyvinylidene fluoride membranes. After 1 h block at room temperature, membranes were reacted with the corresponding primary antibodies of which the details are Fibronectin (1:1000, T59537, Abmart), Collagen I (1:1000, EPR7785, Abmart), Collagen III (1:1000, EPR17673, Abmart), α‐SMA (1:1000, T55295, Abmart, Shanghai), Vimentin (1:2000, ab92547, Abcam), N‐cadherin (1:10 000, ab76011, Abcam), *CBS* (1:5000, 14787‐1‐AP, Proteintech), *MTRR* (1:1000, 26944‐1‐AP, Proteintech), LC3B (1:1000, 14600‐1‐AP, Proteintech), ULK1 (1:1000, 20986‐1‐AP, Proteintech), SQSTM1/p62 (1:2000, ab109012, Abcam), Beclin‐1 (1:2000, T55092, Abmart), STX17 (1:4000, 17815‐1‐AP, Proteintech), GAPDH (1:1000, T0004, Abmart), overnight at 4 °C. Incubation was conducted consequently with HRP‐linked rabbit/mouse IgG antibody (1:3000,7074S/ 7076S, Cell Signaling Technology) for 1 h.

### Protein Immunoprecipitation

For the protein interaction analysis, the Immunoprecipitation Kit with Protein A+G Magnetic Beads (Beyotime, P2179S) was employed, which enabled highly specific and sensitive capture of target proteins. The initial step involved tissue sample preparation using a nondenaturing lysis buffer (Abcam, ab156034) to maintain the native protein conformations and preserve crucial protein–protein interactions and posttranslational modifications. Following tissue homogenization into a uniform suspension, the immunoprecipitation protocol was proceeded with according to the manufacturer's instructions. The pull‐down assay was performed using two primary antibodies: 4 µg of anti‐Ubiquitin antibody (10201‐2‐AP, Proteintech) to identify ubiquitinated proteins, given ubiquitination's critical role in protein regulation and degradation, and an antihomocysteine antibody to investigate potential homocysteinylation modifications. To validate the specificity of the immunoprecipitation results, normal mouse IgG (P2179S‐6, Beyotime) served as a negative control, enabling to distinguish genuine protein interactions from nonspecific binding events. Subsequently, the immunoprecipitated proteins were analyzed through Western blot analysis, with particular emphasis on STX17 detection. The Western blot procedure was executed as previously described, utilizing a high‐sensitivity detection system for precise protein band visualization.

### Mendelian Randomization

Instrumental variables were extracted from the largest publicly available GWAS of serum homocysteine (GCST90013346). Variants were screened in three consecutive steps: i) genome‐wide significance (*p* < 5 × 10^−^⁸); ii) independence (pairwise *r* ^2^ < 0.01, distance ≥ 10 Mb); iii) absence of palindromic or ambiguous alleles. The harmonization routine implemented in TwoSampleMR (v0.6.20) was applied to align effect alleles between exposure and outcome datasets, yielding a final set of 13 robust SNPs. Instrument strength was verified by calculating *F*‐statistics (all >30). 13 verified SNPs about serum Hcy level were selected as instrumental variables for the MR analysis.^[^ ^52^ ^]^ IPF‐associated GWAS summary data were downloaded in the studies of the GWAS Catalog (Study accession: GCST90399722).^[^ ^53^ ^]^

Five MR approaches, including IVW, simple median, MR–Egger, SM, and WM, were executed using the TwoSampleMR package in R (version 4.3.1).^[^ ^54^ ^]^ Cochran's *Q* test was calculated to assess the degree of heterogeneity across the individual effect estimates derived from every genetic variant. The MR–Egger intercept test was conducted to assess the horizontal pleiotropy and a funnel plot was plotted to provide a visual inspection.^[^ ^55^ ^]^ Leave‐one‐out sensitivity analysis was performed to measure if the pooled estimate was being disproportionately influenced by each genetic variant.

### Transcriptome Datasets Acquisition and Analysis

“Pulmonary fibrosis” and “Lung fibrosis” were used as keywords to search for related microarray datasets in the GEO database (https://www.ncbi.nlm.nih.gov/geo/). Three datasets of samples of human lung tissues were selected (GSE231693, GSE199949, GSE52463). Both the GSE231693 and the GSE199949 data sets were based on the GPL20301 platform (Illumina HiSeq 4000[Homo sapiens]). The GSE52463 data set was based on the GPL11154 (Illumina HiSeq 2000[Homo sapiens]). All above included microarray samples from the lung tissues of IPF patients and healthy controls. Two samples, GSM7905760 and GSM8087031 were acquired from public dataset GSE248082 for spatial gene expression transcriptomics analysis. Public scRNA‐seq data were acquired from gene expression matrices of GSE122960, GSE190889, and GSE132771 on the GEO database.

GEO2R (http://www.ncbi.nlm.nih.gov/geo/geo2r) was used to identify DEGs between IPF and non‐IPF samples in dataset GSE23193. Genes that meet statistical significance threshold of adj. *p*‐value < 0.05 and |log2FoldChange| > 1.0 were defined as significant DEGs for subsequent analysis. The heatmap was made by R statistical software (R Core Team, version 4.3.1) and the package pheatmap. Volcano plots were made by the package ggplot2.

GO and KEGG pathway enrichment analyses were performed by the R package cluster profiler. Afterward, the org.Hs.eg.db (version 3.4.0) and enrichplot R packages were used to realize the visualization of enrichment analyzes results.

Search Tool for the Retrieval of Interacting Genes Database (https://cn.string‐db.org/) was used to construct PPI networks based on the identified DEGs in gene lists of hsa00270 (14 genes), hsa00670 (4 genes), and hsa00790 (6 genes). Results were visualized by Cytoscape 3.10.118.

Gene expression matrix from GSE199949 was used to analyze the relevance between genes of fibrosis and key Hcy metabolism genes. Visualization was based on the R package ggcorrplot.

### ScRNA‐Seq Analysis

All scRNA‐seq data were first processed using the Seurat R package version 2.3.4. Three datasets were treated in same steps as followed. All Seurat objects were combined into a merged dataset, and a percentage of mitochondrial genes was calculated for each sample in the merged object. For quality control, cells containing less than 500 identified genes or more than 10% of reads arising from mitochondrial genes were removed. SCTransform with default parameters was used to normalize and scale the data. Dimensionality reduction was performed on the top 2000 most variable genes using PCA. Clustree R package was used to determine the best resolution when clustering. Different groups were manually annotated with known cell type markers reported by Adams et al.^[^ ^56^ ^]^ Violin plots and UMAP plots overlaid with gene expression level were generated using Seurat. Differentially expressed genes of each cluster were identified using FindAllMarkers function of Seurat. R package ggplot2 was used to prettify default figures created using Seurat.

To generate cellular pseudotemporal trajectories, the monocle R package v2.6.4 was used. Cells were ordered in an unsupervised manner (using genes with mean expression >1 and empirical dispersion ≥2*dispersion model estimate) and the resulting pseudotime values were scaled between 0 and 1.

### Quantification and Statistical Analyses

All experiments were performed 3 times independently to improve the data reliability. The one‐way analysis of variance (ANOVA) for multiple group comparisons was utilized to assess the statistical significance of differences, and statistical differences between 2 groups were computed by an unpaired two‐tailed *t*‐test. Experimental statistical analyses were performed in Graphpad Prism 9.0.0 for Windows (GraphPad, San Diego, CA, USA). At least three biological replicates were performed for all experiments.

Clinical data were analyzed using SPSS software (ver. 20.0; SPSS, Inc., Chicago, IL, USA). The distribution of data was assessed using the Shapiro–Wilk test. Student's *t*‐test or the Mann–Whitney *U* test was used to analyze continuous data. Categorical data were compared using the χ2 test. Correlations between Hcy levels and other parameters were analyzed using Spearman's correlation coefficient. ROC curve analyses were used to identify Hcy levels in lung homogenates or BALF that could be used as values for predicting IPF severity. The specific cutoff value was identified by the largest calculated Youden index (Sp + Se − 1). Skewed data are expressed as medians with 25% and 75% quartiles; normally distributed data were expressed as the means ± standard errors of the means. *p* < 0.05 was considered to indicate significant differences.

## Conflict of Interest

The authors declare no conflict of interest.

## Author Contributions

J.H. and K.F. contributed equally and co‐first authors to this work. K.F. and S.L. conceived the project and designed experiments. R.W., Y.C., and X.F. processed the BALF and tissue samples and collected cases information. K.F. performed most of most of the experiments and bioinformatic analysis. K.F., W.L., L.L., and S.L. designed and performed cell culture experiments. K.F., Z.H., Z.C.,Y.J., and J.D. designed and performed in vivo experiments. K.F. conducted data analysis and wrote the original paper. J.H. acquired funding support. Z.L., L.C., and S.L. reviewed and edited the paper. All authors have read and approved the final paper.

## Supporting information



Supporting Information

Supporting Information

Supporting Information

Supporting Information

## Data Availability

The data that support the findings of this study are openly available in GEO at https://www.ncbi.nlm.nih.gov/geo/query/acc.cgi?acc=GSE231693, reference number 231693.
