# Supplementary material for: Homocysteine Exacerbates Pulmonary Fibrosis via Orchestrating Syntaxin 17 Homocysteinylation of Alveolar Type II Cells
Source: Adv Sci (Weinh). 2025 Sep 24;12(46):e07803. doi: 10.1002/advs.202507803 (PMC12697838; doi:10.1002/advs.202507803)
Supplement: Supplementary file 1 — Supporting Information [file ADVS-12-e07803-s004.pdf]

The pdf file includes:

Figs. S1 to S15

Tables S1 to S2

Legend for data file S1, S2 and S3

Other Supplementary Material for this manuscript includes the following:

Data file S1

Data file S2

Data file S3

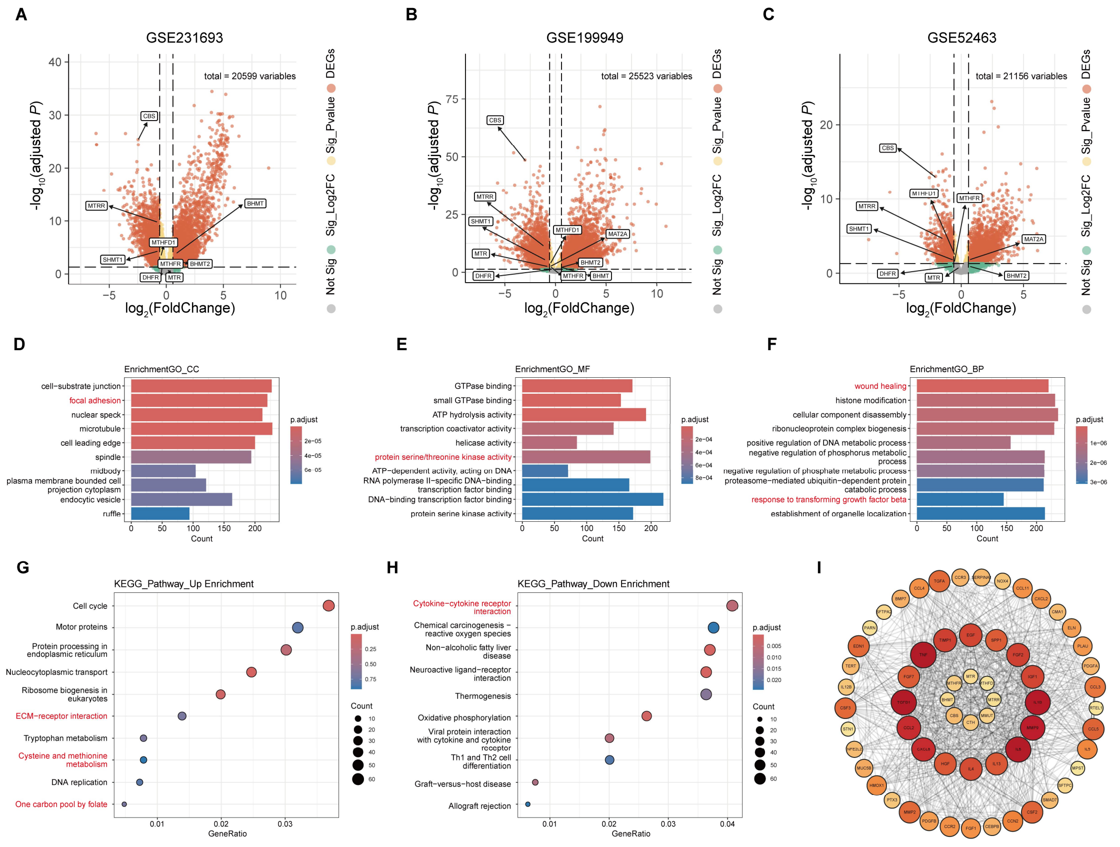

Fig. S1. Bulk-seq transcriptome analysis of IPF samples indicating a potential relationship between Hcy metabolism and IPF, related to Figure 2.

(A-C) Volcano plots showing Hcy metabolism related genes in differential expressed genes (DEGs). Data source: GSE231693 (A), GSE199949 (B), GSE52463 (C).

(D-F) Top 10 GO enrichment results of DEGs. CC, cellular component (D); MF, molecular function (E); BP, biological process (F).

(G-H) Top 10 KEGG pathways enriched from downregulated (G) and upregulated (H) DEGs.

(I) Protein-protein interaction (PPI) network analysis of DEGs related to Hcy metabolism, inflammation and fibrosis.

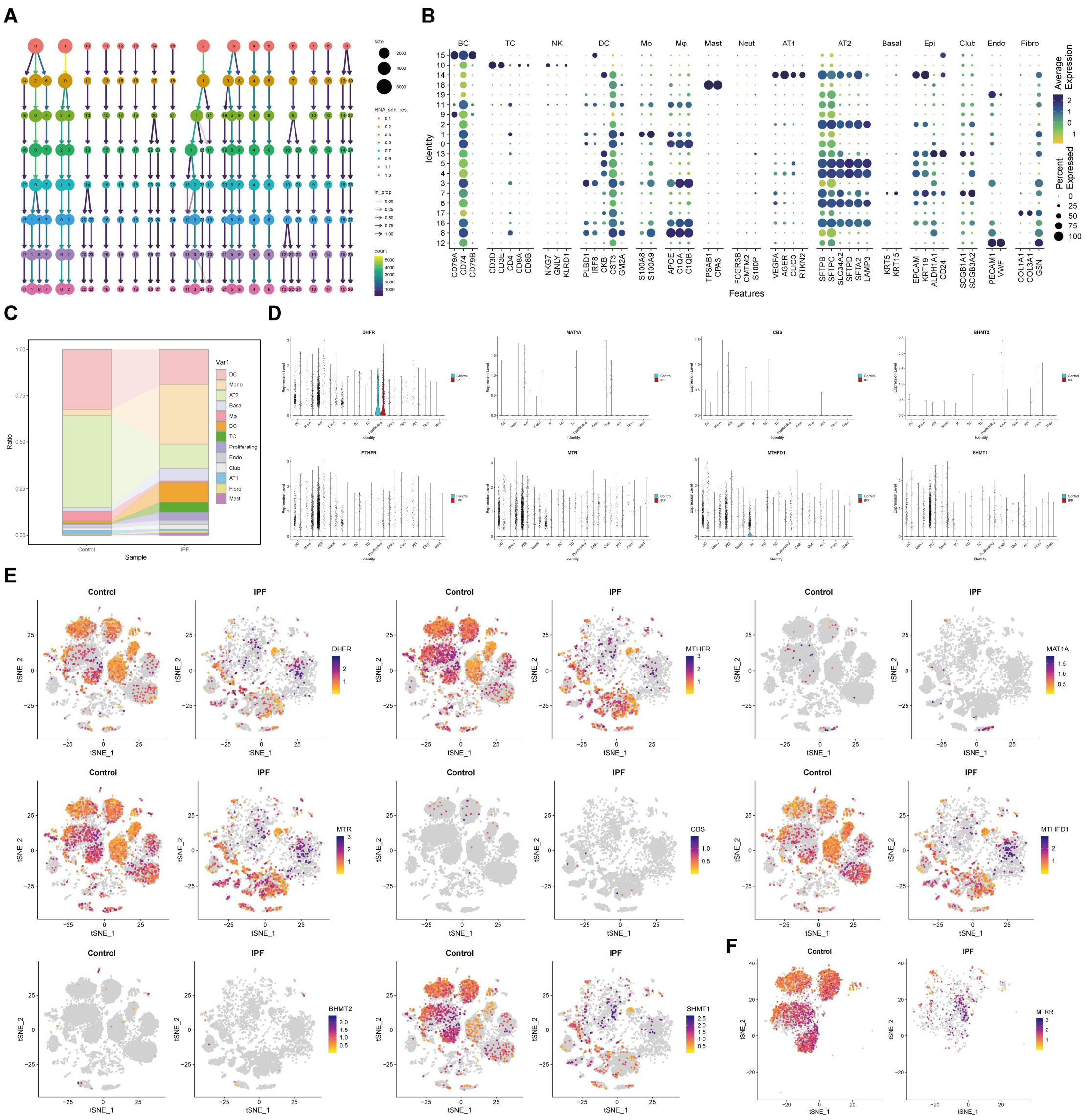

Fig. S2. Single-cell transcriptional profile analysis of different cell types between human IPF and healthy control samples, related to Figure 2.

(A) Tree diagram created by Clustree R package to define the best resolution of clustering.

(B) Dot plot of different cell type features annotating cell clusters.

(C) Comparison of percent of different cells in IPF and control group.

(D) Violin plots showing Hcy metabolism genes expression level among different cell clusters.

(E) Feature plot demonstrating the difference of Hcy metabolism genes expression level among cells from IPF and control samples.

(F) Feature plot visualizing *MTRR* expression level among all lung cells from the integrated single-cell RNA sequencing dataset (healthy and IPF samples combined).

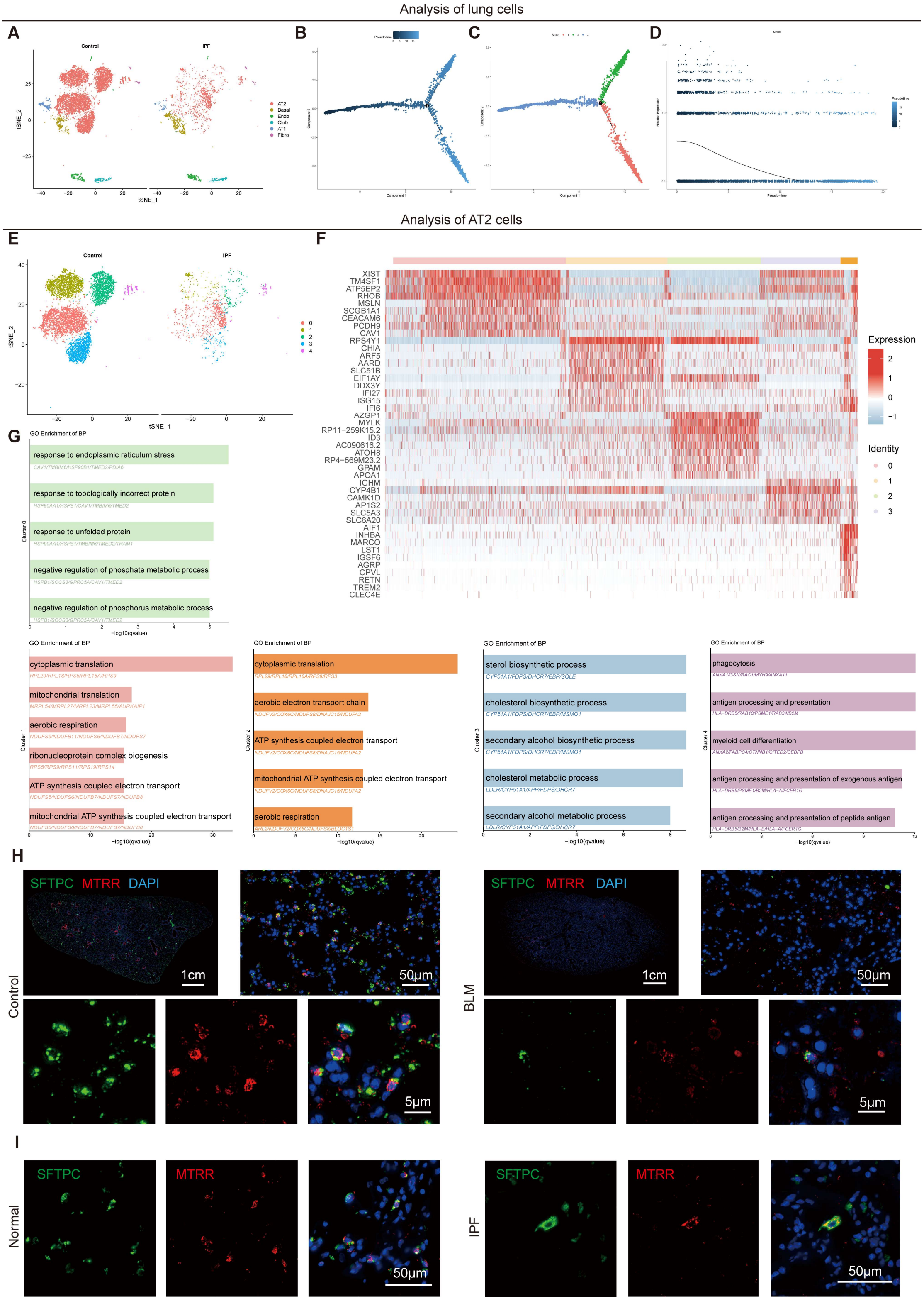

Fig. S3. Single cell analysis and colocalization of AT2 cells, related to Figure 2 and Figure 3.

(A) t-SNE plot showing 6 main cell types of lung cells.

(B-D) Pseudo-time analysis lung cells.

(E) t-SNE plot showing 5 clusters of AT2 cells.

(F) Heatmap showing DEGs between 5 AT2 cell clusters.

(G) Function identification of AT2 cell clusters using GO enrichment of biological process.
(H) IF staining against SFTPC and MTRR showing decreased MTRR expression level in AT2 cells of mouse tissues (n = 3).
(I) IF staining against SFTPC and MTRR showing decreased MTRR expression level in AT2 cells of human lung slices (n = 3).

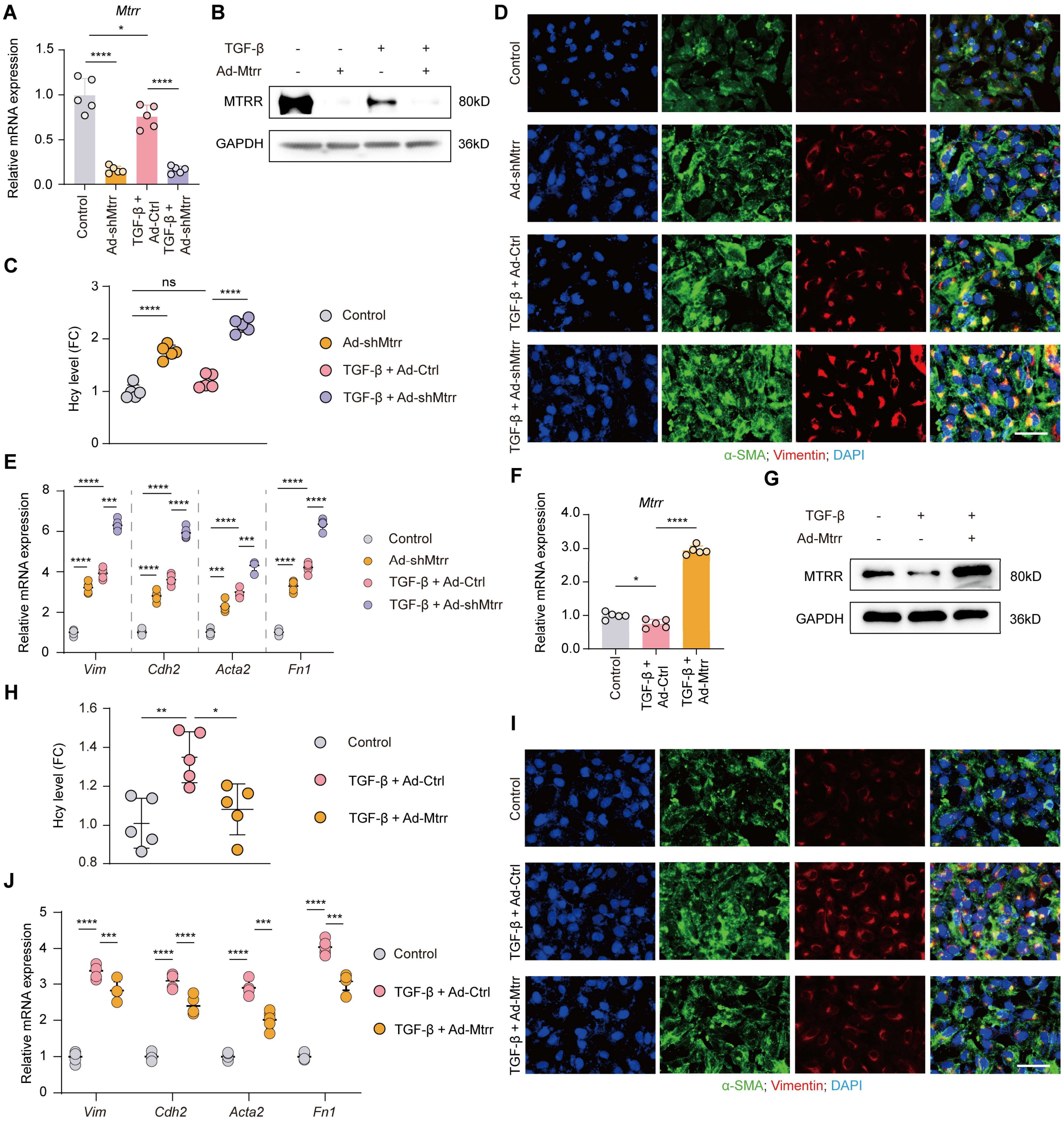

Fig. S4. MTRR expression influences epithelial-mesenchymal transition, related to Figure 4.

(A) Relative *Mtrr* mRNA expression in TGF-β treated AT2 cells which transfected with Ad-Ctrl or Ad-sh*Mtrr* (multiplicity of infection = 10) (n = 5 per group).

(B) Western blot showing knocked-down MTRR expression level after adenovirus transfection.

(C) Hcy level in differently treated AT2 cells, represented by fold changes (n = 5).

(D) IF staining against α-SMA and vimentin showing enhanced epithelial-mesenchymal transition (EMT) process in MTRR knocked down AT2 cells (n = 5).

(E) Relative mRNA expression level of key EMT genes (*Vim*, *Cdh2*, *Acta2*, *Fn1*) (n = 5).

(F) Relative *Mtrr* mRNA expression in TGF-β treated AT2 cells which transfected with Ad-GFP or Ad-sh*Mtrr* (multiplicity of infection = 10) (n = 5 per group).

(G) Western blot showing overexpressed MTRR expression level after adenovirus transfection.

(H) Overexpression of MTRR downregulated Hcy level in AT2 cells (n =5).
(I) IF staining against α-SMA and vimentin showing inhibited EMT process in AT2 cells with the recovery of MTRR expression.
(J) Relative *Mtrr* mRNA expression in TGF-β treated AT2 cells which transfected with Ad-GFP or Ad-*Mtrr* (multiplicity of infection = 10) (n = 5 per group).
Data are presented as the mean ± SEM. Significance was determined by one-way ANOVA with Tukey's multiple comparison tests (A, C, F and H) and two-way ANOVA with Tukey's multiple comparison tests (E and J). *p< 0.05; ***p < 0.001; ****p < 0.0001.

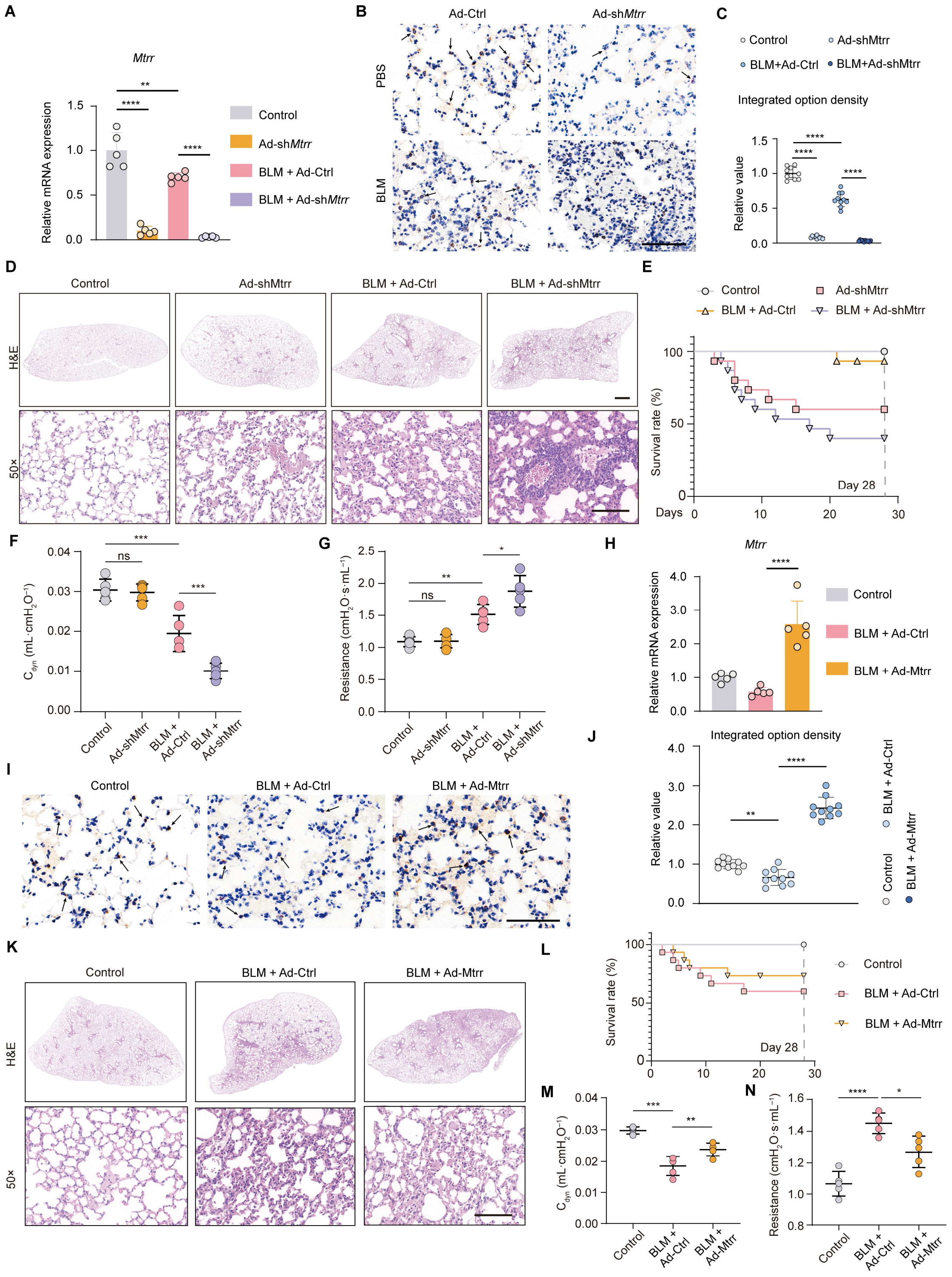

Fig. S5. Genetic editing of *Mtrr* impacts pulmonary fibrosis, related to Figure 4.

(A) RT-PCR results showing *Mtrr* expression level after gene knock down.

(B and C) IHC staining against MTRR exhibiting its distribution (B) and quantified amount (C) in different groups.

(D) H&E staining of mouse lung sections showing fibrosis features (n = 5 per group). Scale bars=1 mm. Images in the lower panels were magnified from the photomicrographs in the upper panels. Scale bars=100 μm.

(E) Survival curve showing a severe mortality after *Mtrr* knock down.

(F and G) Pulmonary function detection of dynamic lung compliance (F) and lung resistance (G) (n = 5 per group).
(H) RT-PCR results showing *Mtrr* expression level after gene overexpression.
(I and J) IHC staining against MTRR exhibiting the expression level after over-expression of *Mtrr* via adenovirus treatment (I) and the quantification of the expression level in three groups (J). Scale bars=100 μm.
(K) H&E staining of mouse lung sections showing fibrosis features (n = 5 per group). Scale bars=1 mm. Images in the lower panels were magnified from the photomicrographs in the upper panels. Scale bars=100 μm.
(L) Survival curve showing protective effect of *Mtrr* over-expression (n = 5 per group).
(M and N) Pulmonary function detection of dynamic lung compliance (M) and lung resistance (N) (n = 5 per group).
Data are presented as the mean ± SEM. Significance was determined by log-rank (Mantel-Cox) test (E, L), one-way ANOVA with Tukey's multiple comparison tests (C, F, G, H, J, M and N). ns: no significance; *$p < 0.05$; **$p < 0.01$; ***$p < 0.001$; ****$p < 0.0001$.

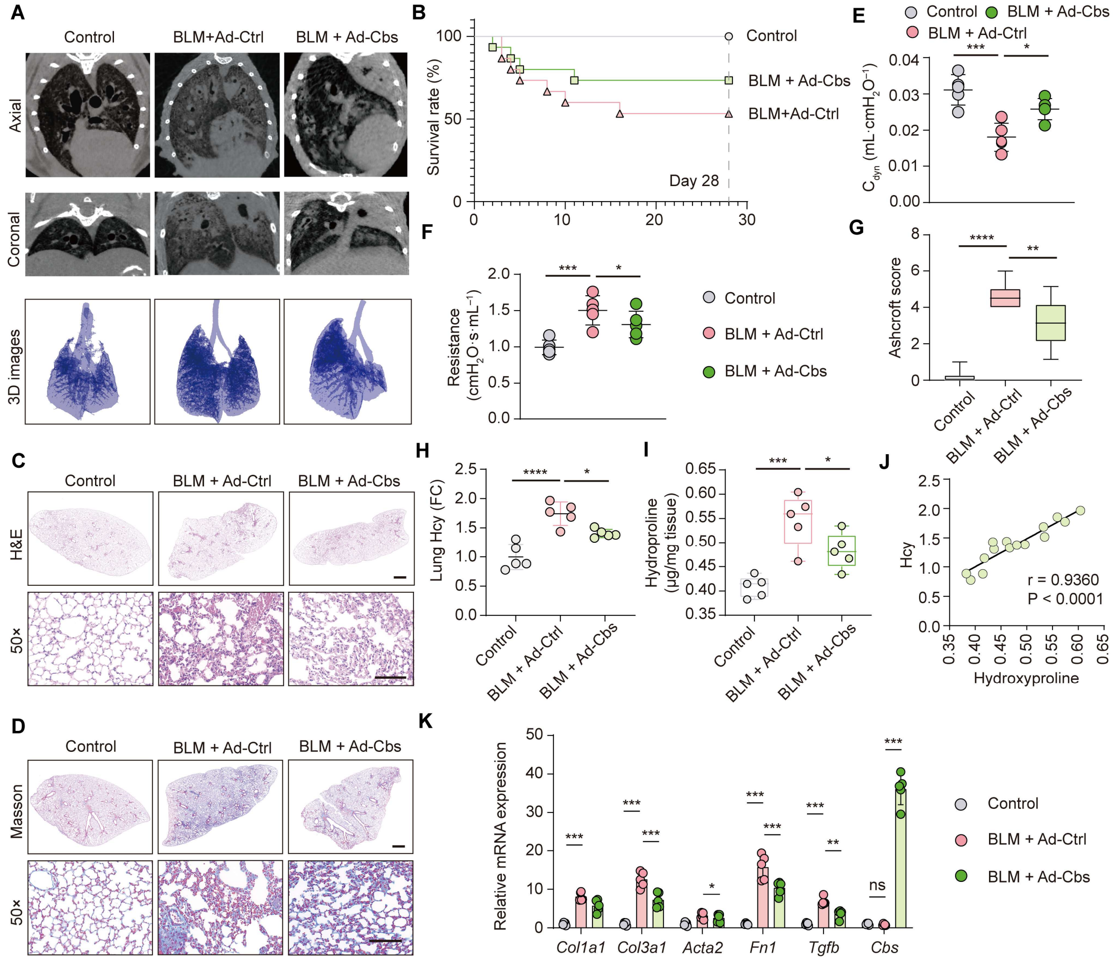

Fig. S6. *Cbs* overexpression ameliorates Hcy metabolism and improves fibrosis, related to Figure 4.

(A) Micro-CT and its 3D-recontruction images of modeled mice showing pathologic characteristics of fibrosis (n = 5 per group).

(B) Survival curve of the three groups (n = 5 per group).

(C) H&E staining demonstrating significant fibrosis in lung sections (n = 5 per group). Scale bars=1 mm. Images in the lower panels were magnified from the photomicrographs in the upper panels. Scale bars=100 μm.

(D) Masson trichome staining showing collagen distribution in modeled mice (n = 5 per group). Scale bars=1 mm. Images in the lower panels were magnified from the photomicrographs in the upper panels. Scale bars=100 μm.

(E and F) Lung function tests of dynamic lung compliance (E) and lung resistance (F) (n = 5 per group).

(G) Ashcroft score quantifying IPF severity (n = 5).

(H) Hcy concentration measurement of lung homogenates, showing with fold changes (FC).

(I) Hydroxyproline concentration measurement of lung homogenates (n = 5).

(J) Correlation analysis for hydroxyproline (x-axis) *vs*. Hcy level(y-axis).

(K) Relative mRNA expression of fibrotic genes measured by RT-PCR (n = 5).

Data are presented as the mean ± SEM. Significance was determined by log-rank

(Mantel-Cox) test (B), one-way ANOVA with Tukey's multiple comparison tests (E, F, G, H and J) and two-way ANOVA with Tukey's multiple comparison tests (K). $^{*}p < 0.05$; $^{**}p < 0.01$; $^{***}p < 0.001$; $^{****}p < 0.0001$.

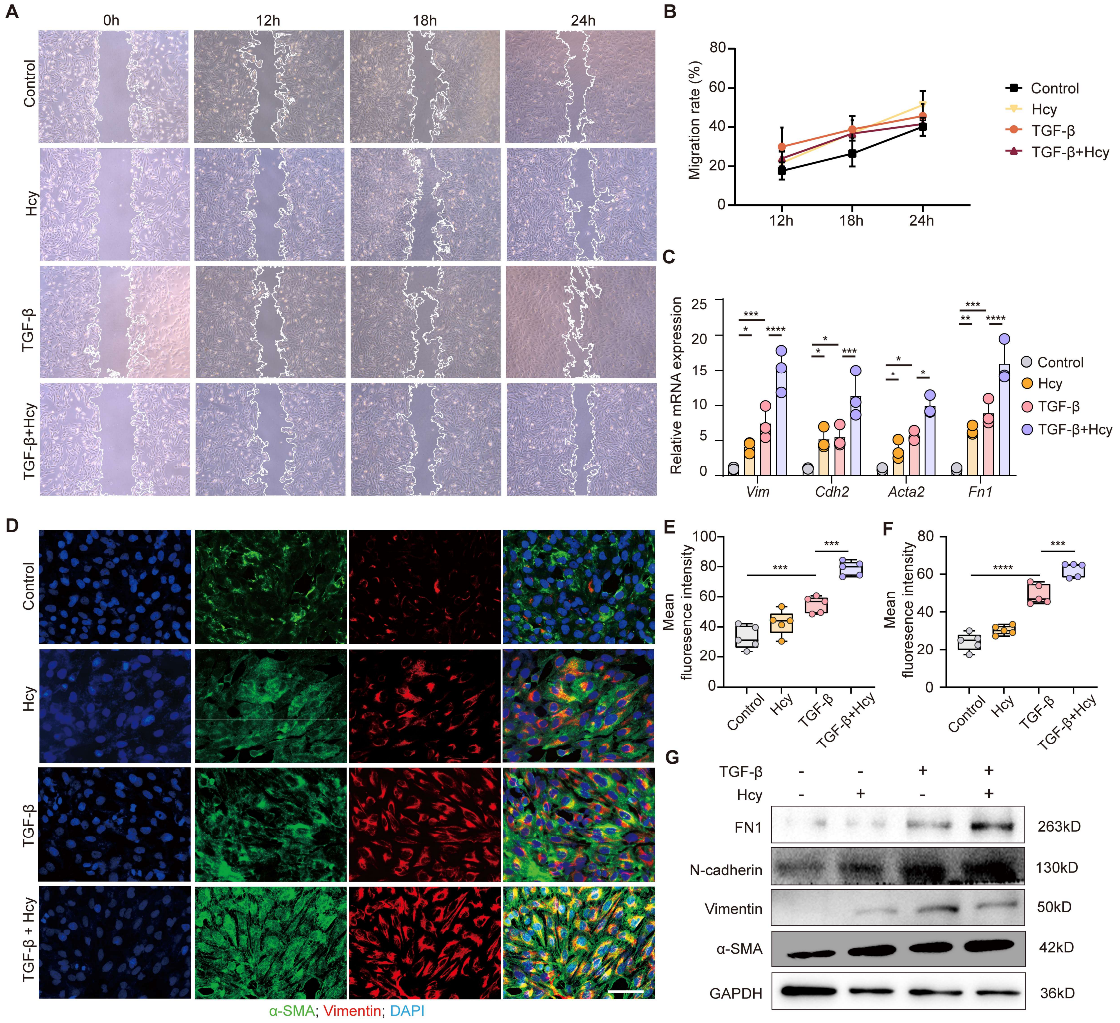

Fig. S7. Hcy accelerates epithelial-mesenchymal transition process in vitro, related to Figure 5.

(A and B) Wound healing experiment testing EMT speed of AT2 cells treated with Hcy (20 nM) or not in the presence of TGF-β (10 ng/ml) (A) symbolized by cell migration rate (B) (n = 3).

(C) Relative mRNA expression of EMT genes measured by RT-PCR (n = 3).

(D-F) IF staining against α-SMA and vimentin and their quantification (n = 5).

(G) Western blots detecting expression level of EMT markers (FN1, N-cadherin, Vimentin and α-SMA).

Data are presented as the mean ± SEM. Significance was determined by two-way ANOVA with Tukey's multiple comparison tests (C) and one-way ANOVA with Tukey's multiple comparison tests (E and F). *$p < 0.05$; **$p < 0.01$; ***$p < 0.001$; ****$p < 0.0001$. ns, no significance. Scale bar = 50 μm.

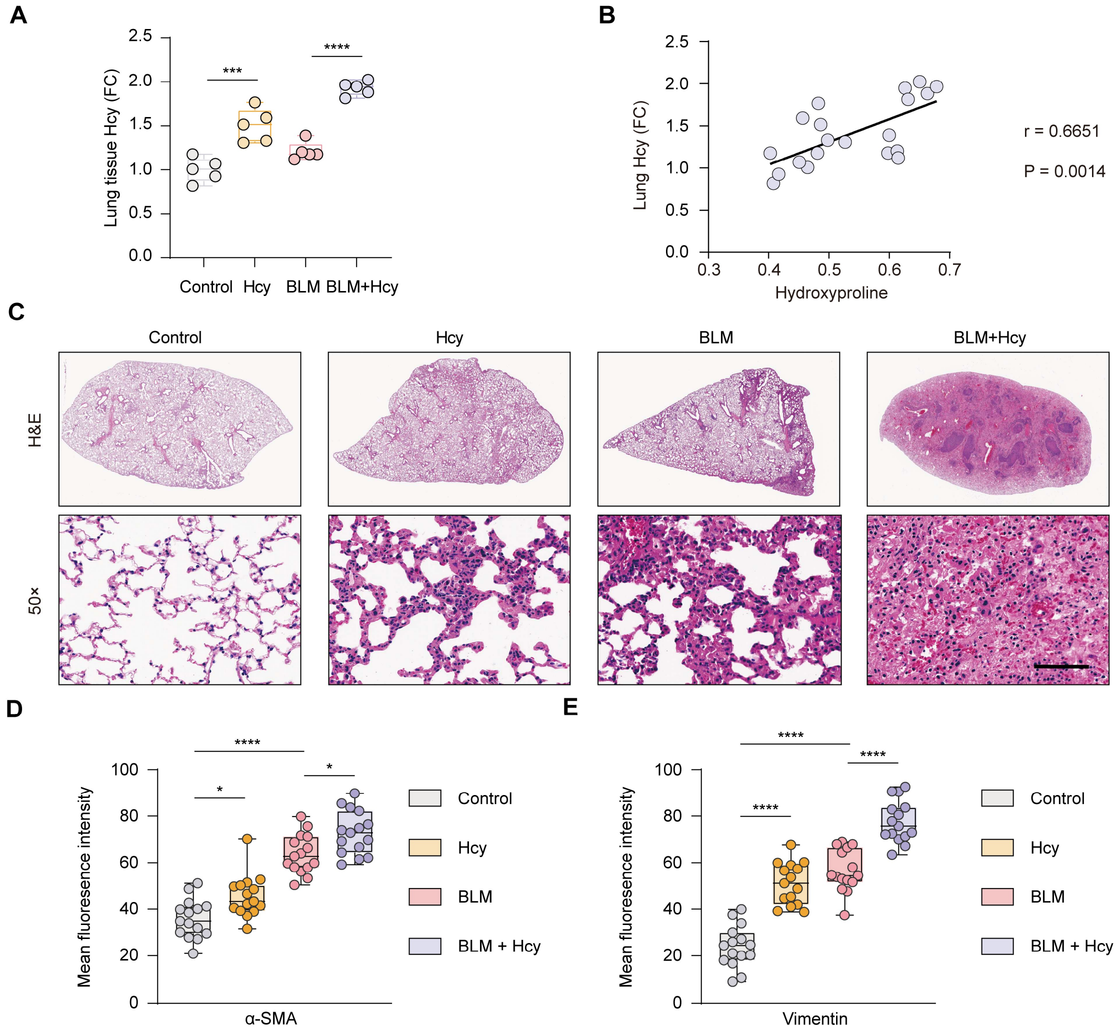

Fig. S8. Hcy supplement aggravates pulmonary fibrosis, related to Figure 5.

(A) Hcy concentration of lung homogenates measured by Hcy ELISA kit (n = 5 per group).

(B) Correlation line showing the positive relationship between hydroxyproline and Hcy.

(C) H&E staining of lung sections. Scale bars=1 mm. Images in the lower panels were magnified from the photomicrographs in the upper panels. Scale bars=100 µm (n = 5).

(D and E) Quantification of fluorescence intensity showing the relative expression level of α-SMA (D) and vimentin (E) (n = 15).

Data are presented as the mean ± SEM. Significance was determined by one-way ANOVA with Tukey's multiple comparison tests. *$p < 0.05$; ***$p < 0.001$; ****$p < 0.0001$.

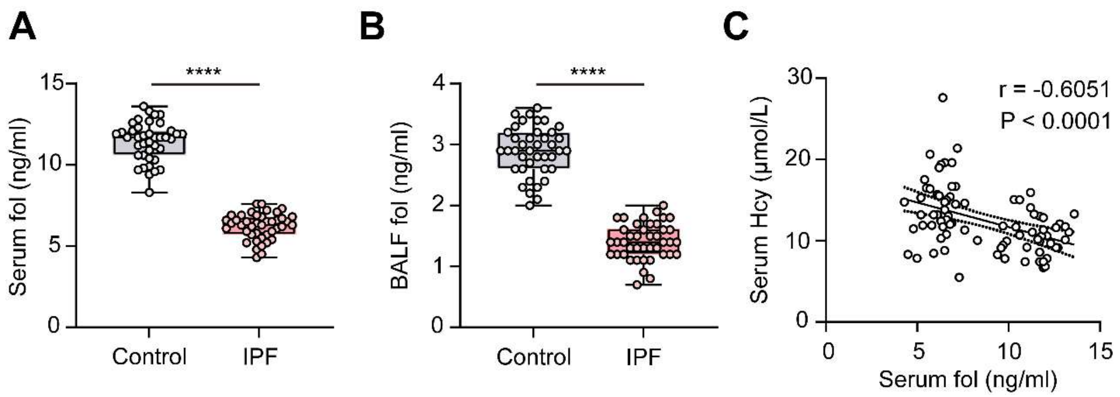

Fig. S9. Comparison of Fol level in normal control (n = 40) and IPF patients (n = 42), related to Figure.

(A) Comparison of Fol level in serum.

(B) Comparison of Fol level in serum.

(C) Relativity analysis between serum Fol and Hcy.

Data are presented as the mean ± SEM. ****p < 0.0001 by Student's *t* tests.

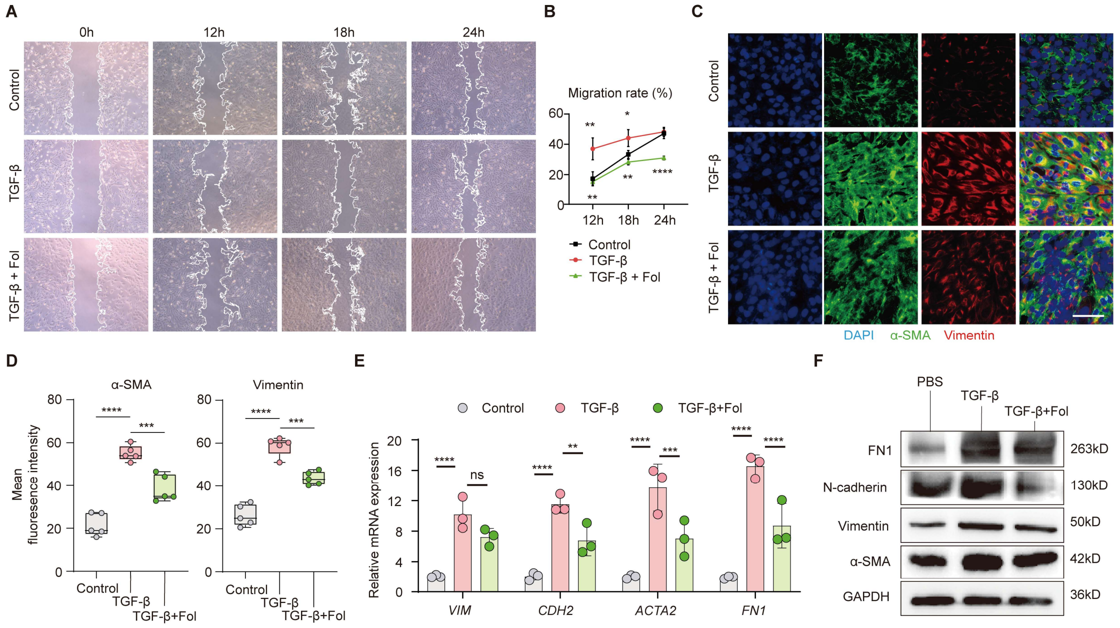

Fig. S10. Folate treatment ameliorates EMT process in TGF-β treated AT2 cells, related to Figure 6.

(A and B) Scratching test (A) and its quantification (B) showing decreased migration rate after folate treatment of AT2 cells.

(C and D) IF staining against α-SMA and vimentin (C) and fluorescence intensity quantification (D) demonstrating inhibited EMT process in AT2 cells co-treated with TGF-β and folate.

(E) RT-PCR showing EMT genes expression level in AT2 cells.

(F) Western blot analysis of EMT and fibrosis proteins in AT2 cell lysis.

Data are presented as the mean ± SEM. Significance was determined by one-way ANOVA with Tukey's multiple comparison tests (B and D) and one-way ANOVA with Tukey's multiple comparison tests (E). $*p < 0.05$; $**p < 0.01$; $***p < 0.001$; $****p < 0.0001$. Scale bar = 50 μm.

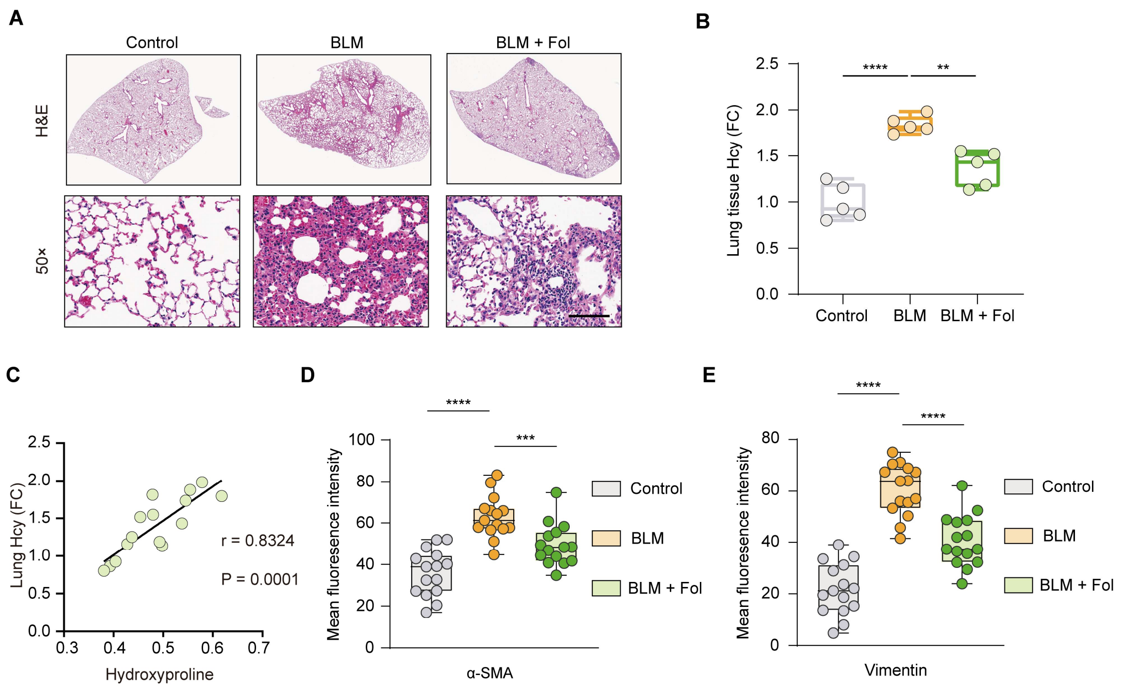

Fig. S11. Folate treatment improves histological and biochemical manifestations in BLM challenged mice, related to Figure 6.

(A) H&E staining showing Folate treatment effect on BLM induced mouse IPF model (n = 5 per group). Scale bars=1 mm. Images in the lower panels were magnified from the photomicrographs in the upper panels. Scale bars=100 μm.

(B) Hcy level in lung homogenates, demonstrated as fold change (FC) (n = 4 per group).

(C) Correlation analysis between hydroproline and lung Hcy level.

(D and E) Mean fluorescence intensity showing α-SMA (D) and vimentin (E) expression level (n = 15).

Data are presented as the mean ± SEM. Significance was determined by one-way ANOVA with Tukey's multiple comparison tests (B, D and E). $^{**}p < 0.01$; $^{***}p < 0.001$; $^{****}p < 0.0001$.

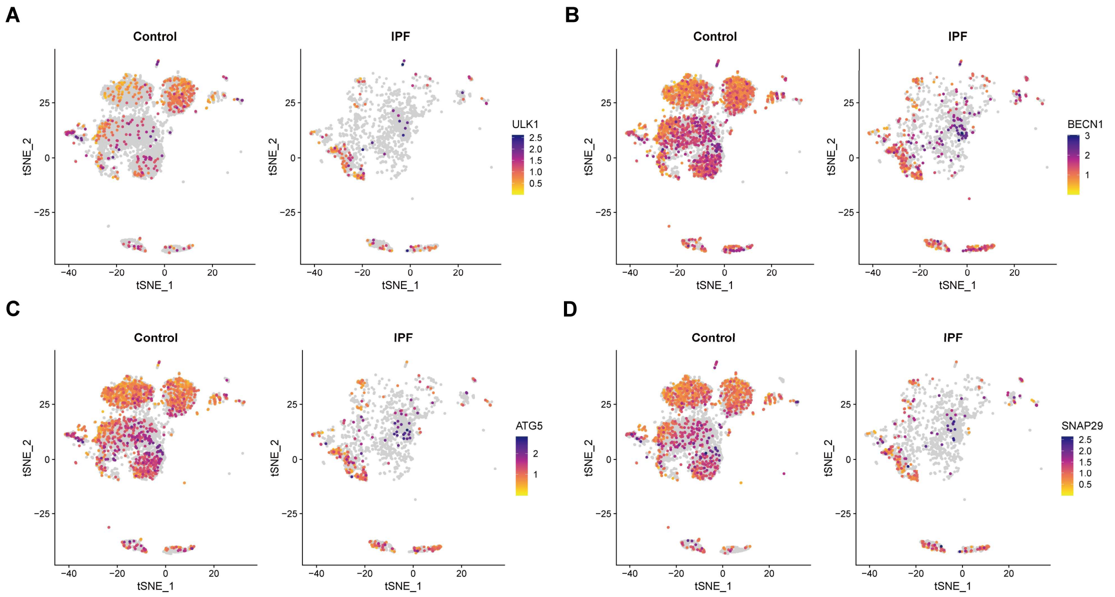

Fig. S12. Single cell analysis of lung cells indicates differential expression of four key autophagy genes between control and IPF group, related to Figure 7.

(A) Expression level of *ULK1* under the reduction of t-SNE.

(B) Expression level of *BECN1* under the reduction of t-SNE.

(C) Expression level of *ATG5* under the reduction of t-SNE.

(D) Expression level of *SNAP29* under the reduction of t-SNE.

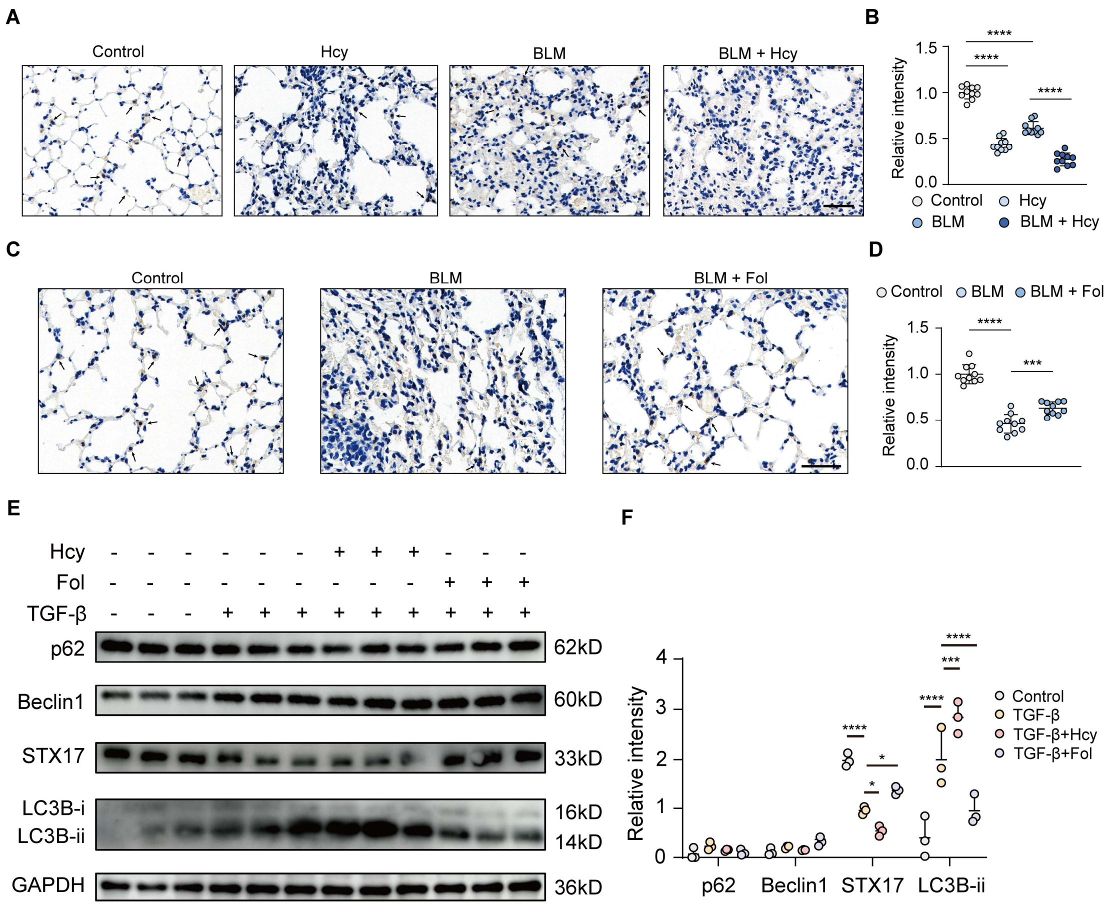

Fig. S13. Immunohistochemical staining and western blot of STX17 protein showing inverse effect on autophagy of Hcy and folate treatment, related to Figure 7.

(A and B) Extra Hcy supplement showing the inhibition of STX17 expression (n = 10 per group).

(C and D) Folate treatment reversed BLM induced STX17 downregulation in IPF model (n = 10 per group). Scale bars = 100 μm.

(E) Representative Western blots analyzing autophagy proteins (Beclin1, p62, LC3B) and SNARE protein component STX17 after Hcy or Fol treatment.

(F) Quantification of the blot intensity (compared to GAPDH expression level).

Data are presented as the mean ± SEM. Significance was determined by one-way ANOVA with Tukey's multiple comparison tests. ***$p < 0.001$; ****$p < 0.0001$.

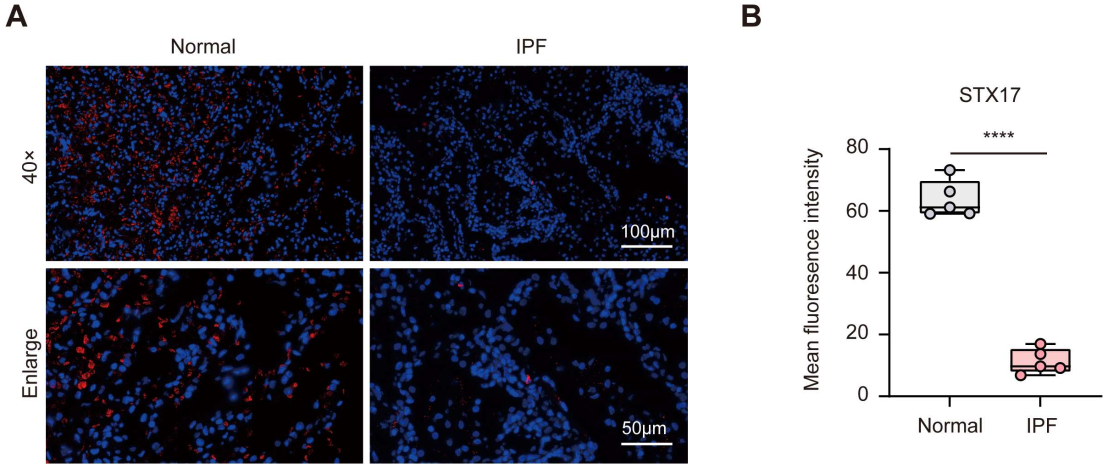

Fig. S14. STX17 expression in human lung tissues from normal control and IPF patients, related to Figure 7.

(A and B) IF staining against STX17 showing inhibition of STX17 expression in IPF samples (A) and the quantification of florescence signal (B).

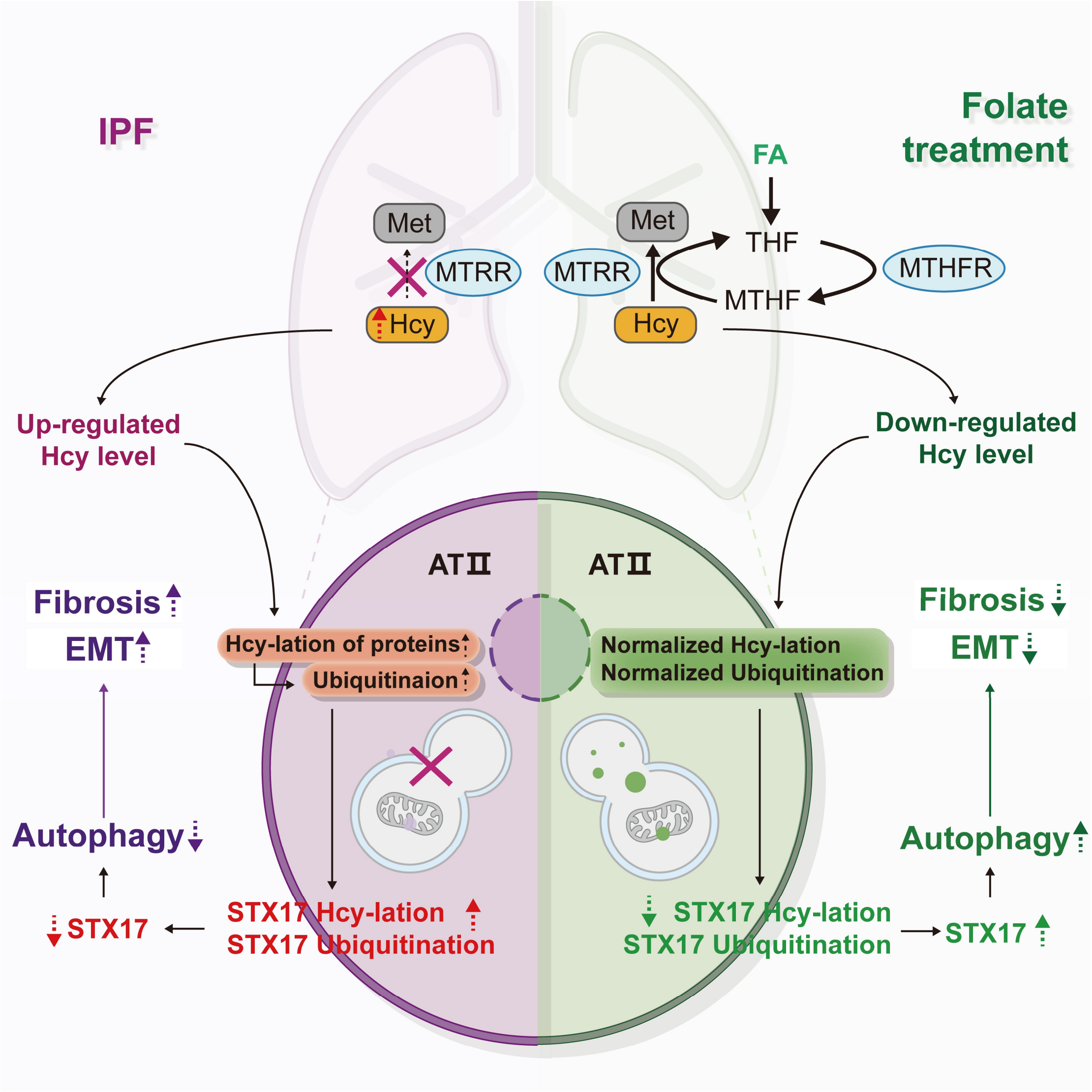

Fig. S15. Graphic abstract demonstrating changes of Hcy metabolism and autophagy process in the IPF environment.

Table S1. Primers used for the analysis of mRNA expression

Type	Gene	Forward Primer	Reverse Primer
Mouse	*β-Actin*	ATATCGCTGCGCTGGTCGTC	AGGATGGCGTGAGGGAGAGC
	Acta2	GTCCCAGACATCAGGGAGTAA	TCGGATACTTCAGCGTCAGGA
	Col1a1	GCTCCTCTTAGGGGCCACT	CCACGTCTCACCATTGGGG
	Col3a1	CTGTAACATGGAAACTGGGGAAA	CCATAGCTGAACTGAAAACCACC
	Fn1	ATGTGGACCCCTCCTGATAGT	GCCCAGTGATTTCAGCAAAGG
	Tgfb	CCACCTGCAAGACCATCGAC	CTGGCGAGCCTTAGTITGGAC
	Mtrr	GGACAGGCAAAGGCCATAG	ACCCGTGGTAGATACAACCAT
	Mtr	ATGATCCAGCGGTACAAACTAAG	CATCCGGTAGGCCAAGTGTTC
	Mthfr	CTGGGCACTGTTATCCATCCC	TCCTGCTGATAGAGGGTGGC
	Mat1a	GTGCTGGATGCTCACCTCAAG	CCACCCGCTGGTAATCAACC
	Cbs	CCAGGCACCTGTGGTCAAC	GGTCTCGTGATTGGATCTGCT

Table S2. Key resources

REAGENT or RESOURCE	SOURCE	IDENTIFIER
Antibodies		
Monoclonal rabbit anti-FN1	Abmart	Cat.#T59537; RRID: AB_2936783
Monoclonal rabbit anti-Collagen I	Abcam	Cat.#ab138492; RRID: AB_2861258
Monoclonal rabbit anti-Collagen III	Abcam	Cat.#ab184993; RRID: AB_2861260
Monoclonal rabbit anti-alpha smooth muscle Actin	Abmart	Cat.#T55295; RRID: AB_2892572
Monoclonal rabbit anti-Vimentin	Abcam	Cat.#ab92547; RRID: AB_10562134
Monoclonal rabbit anti-N Cadherin	Abcam	Cat.#ab76011; RRID: AB_2581062
Polyclonal rabbit anti-CBS	Proteintech	Cat.#14787-1-AP; RRID: AB_2070970
Polyclonal rabbit anti-MTRR	Proteintech	Cat.#26944-1-AP; RRID: AB_2880694
Polyclonal rabbit anti-LC3	Proteintech	Cat.#14600-1-AP; RRID: AB_2137737
Polyclonal rabbit anti-ULK1	Proteintech	Cat.#20986-1-AP; RRID: AB_2878783
Polyclonal rabbit anti-STX17	Proteintech	Cat.#17815-1-AP; RRID: AB_2255542
Monoclonal rabbit anti-SQSTM1/p62	Abcam	Cat.#ab109012; RRID: AB_2241816
Monoclonal rabbit anti-Beclin 1	Abmart	Cat.#T55092; RRID: AB_2934186
Polyclonal rabbit anti-	Abcam	Cat.#ab15154; RRID: AB_301699

homocysteine		
Polyclonal rabbit anti-ubiquitin	Proteintech	Cat.#10201-2-AP; RRID: AB_671515
Monoclonal mouse anti-GAPDH	Abmart	Cat.#M20006; RRID: AB_2737054
Goat anti-mouse IgG-HRP	Abmart	Cat.#M21001; RRID: AB_2713950
Goat anti-rabbit IgG-HRP	Abmart	Cat.#M21002; RRID: AB_2713951
Bacterial and virus strains		
Ad-ZsGreen-mCbs-Flag overexpressio adenovirus	Viraltherapy Technologies	N/A
Ad-ZsGreen-mMtrr-Flag overexpression adenovirus	Viraltherapy Technologies	N/A
Ad-ZsGreen-shRNA-mMtrr knock-down adenovirus	Viraltherapy Technologies	N/A
Ad-ZsGreen overexpression control adenovirus	Viraltherapy Technologies	N/A
Ad-ZsGreen knock-down control adenovirus	Viraltherapy Technologies	N/A
Chemicals, peptides, and recombinant proteins		
TGF beta 1/TGFB1 Protein	MedChemExpress	Cat.#HY-P70648
Folate	Aladdin	Cat.#F413155
Homocysteine	Sigma-Aldrich	Cat.#H4628
Tetrahydrofolate	Sigma-Aldrich	Cat.#T3125
Trizol	Vazyme	Cat.#R401
HiScript II Q RT SuperMix for qPCR	Vazyme	Cat.#R222-01

ChamQ SYBR qPCR Master Mix	Vazyme	Cat.#Q331-02
RIPA Lysis Buffer	Beyotime	Cat.#P0013B
Protease and phosphatase inhibitor cocktail	Beyotime	Cat.#P1050
Methanol	Aladdin	Cat.#M116118
Ethanol	Aladdin	Cat.#E111964
Avertin	MeilunBio	Cat.#MA0478
Bleomycin	Beyotime	Cat.#ST1450
Collagenase IV	ThermoFisher Scientific	Cat.#17104019
DNase I	Roche	Cat.#11284932001
Dispase II solution	Beyotime	Cat.#ST2339
DAPI	Sigma	Cat.#D9542
4% Paraformaldehyde fixative	Biosharp	Cat.#BL539A
Xylene	Aladdin	Cat.#X112050
5% BSA blocking buffer	Solarbio	Cat.#SW3015
Fetal bovine serum	Sigma-Aldrich	Cat.#F8318
DMEM	ThermoFisher Scientific	Cat.#12491015
Critical commercial assays		
SABC-HRP Kit	Beyotime	Cat.#P0603
Total HCY ELISA kit	Finetest	Cat.#EU20003
Hydroxyproline Assay kit (Colorimetric)	Abcam	Cat.#ab222941
Immunoprecipitation kit with Protein A+G Magnetic Beads	Beyotime	Cat.#P2179S
Pierce BCA Protein Assay Kit	Thermo Fisher Scientific	Cat.#A55864

Masson's Trichrome Staining Kit	Beyotime	Cat.#C0189
Hematoxylin and Eosin Staining Kit	Beyotime	Cat.#C0105
Deposited data		
Bulk RNA-seq data	Nance et al., 2014	GEO: GSE52463
Bulk RNA-seq data	Huang et al., 2023	GEO: GSE199949
Bulk RNA-seq data	Jia et al., 2023	GEO: GSE231693
Single cell RNA-seq data	Reyfman et al., 2019	GEO: GSE122960
Single cell RNA-seq data	Heinzelmann et al., 2022	GEO: GSE190889
Single cell RNA-seq data	Tsukui et al., 2020	GEO: GSE132771
Experimental models: Organisms/strains		
Mouse: C57BL/6J	Shanghai Model Organisms Center, Inc.	Cat. NO. SM-001
Oligonucleotides		
Primers for qPCR, see Table S1	This paper	N/A
Software and algorithms		
ImageJ	Schneider et al., 2012	imagej.net/ij/index.html
Graphpad Prism 9	GraphPad	www.graphpad.com/
IBM SPSS Statistics 29.0.1.0	IBM Corp.	www.ibm.com/spss
Megellan	TECAN	lifesciences.tecan.com/software-magellan
cellSens	Olympus	lifescience.evidentscientific.com.cn/zh/software/cellsens/
Leica LAS X	Leica	N/A
STRING	Szklarczyk D et al., 2023	cn.string-db.org/
Cytoscape	Shannon et al.,	cytoscape.org/

3.10.118	2003	
R	The R Project for Statistical Computing	www.r-project.org/
DESeq2	Love et al., 2014	bioconductor.org/packages/release/bioc/html/DESeq2.html
TwoSampleMR	Hemani G et al., 2018	github.com/MRCIEU/TwoSampleMR
Seurat	Hao et al., 2021	satijalab.org/seurat/
Clustree	Zappia et al., 2018	github.com/lazappi/clustree
ggplot2	Wickham et al., 2016	ggplot2.tidyverse.org/
Monocle2	Qiu et al., 2017	cole-trapnell-lab.github.io/monocle-release/

Data file S1. Raw data for all laboratory experiments.
Data file S2. Raw data for Mendelian Randomization results.
Data file S3. Unedited blots images.
