# Supplementary material for: Homocysteine Exacerbates Pulmonary Fibrosis via Orchestrating Syntaxin 17 Homocysteinylation of Alveolar Type II Cells
Source: Adv Sci (Weinh). 2025 Sep 24;12(46):e07803. doi: 10.1002/advs.202507803 (PMC12697838; doi:10.1002/advs.202507803)

Figure 3

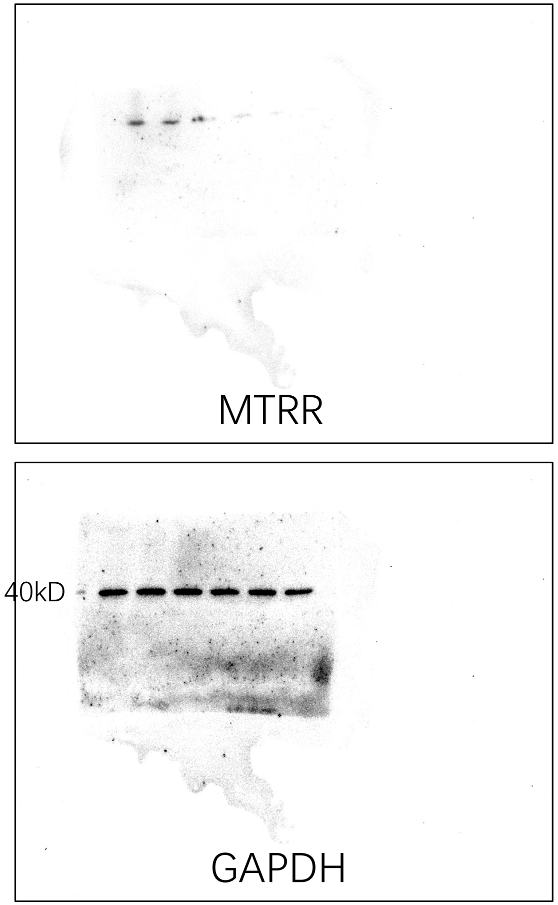

Figure 3F

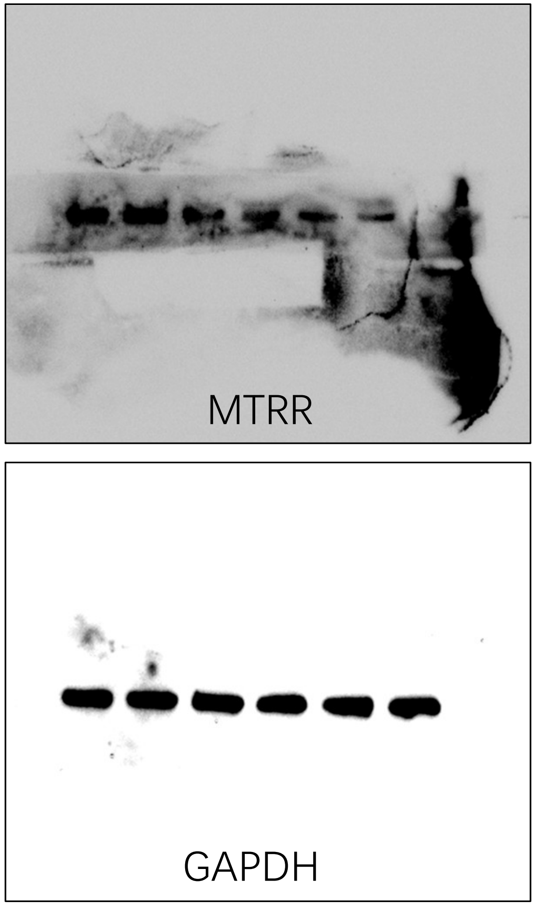

Figure 3J

Figure 5J

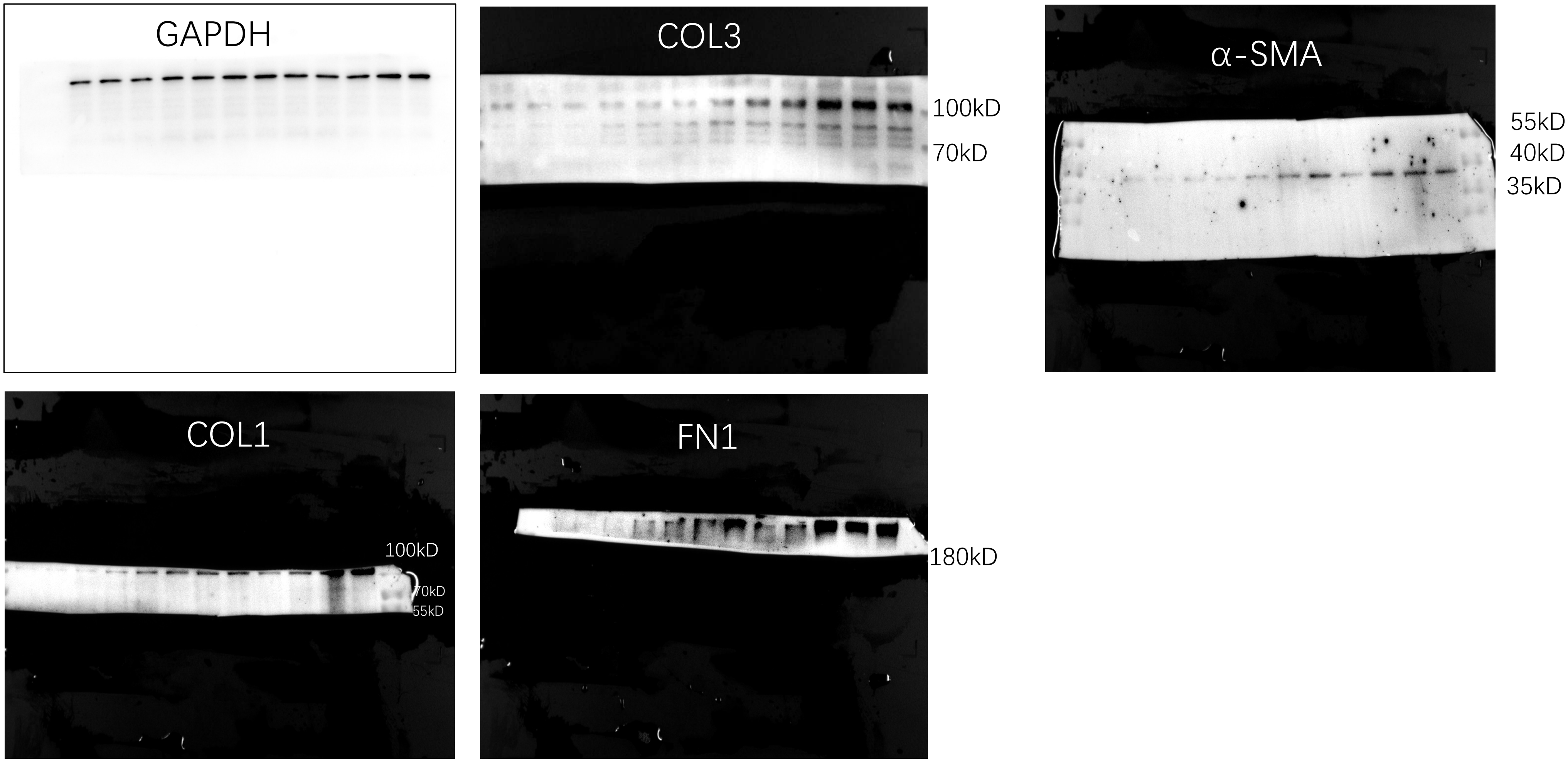
GAPDH
COL3
100kD
70kD
α-SMA
55kD
40kD
35kD
COL1
100kD
70kD
55kD
FN1
180kD

Figure 6L

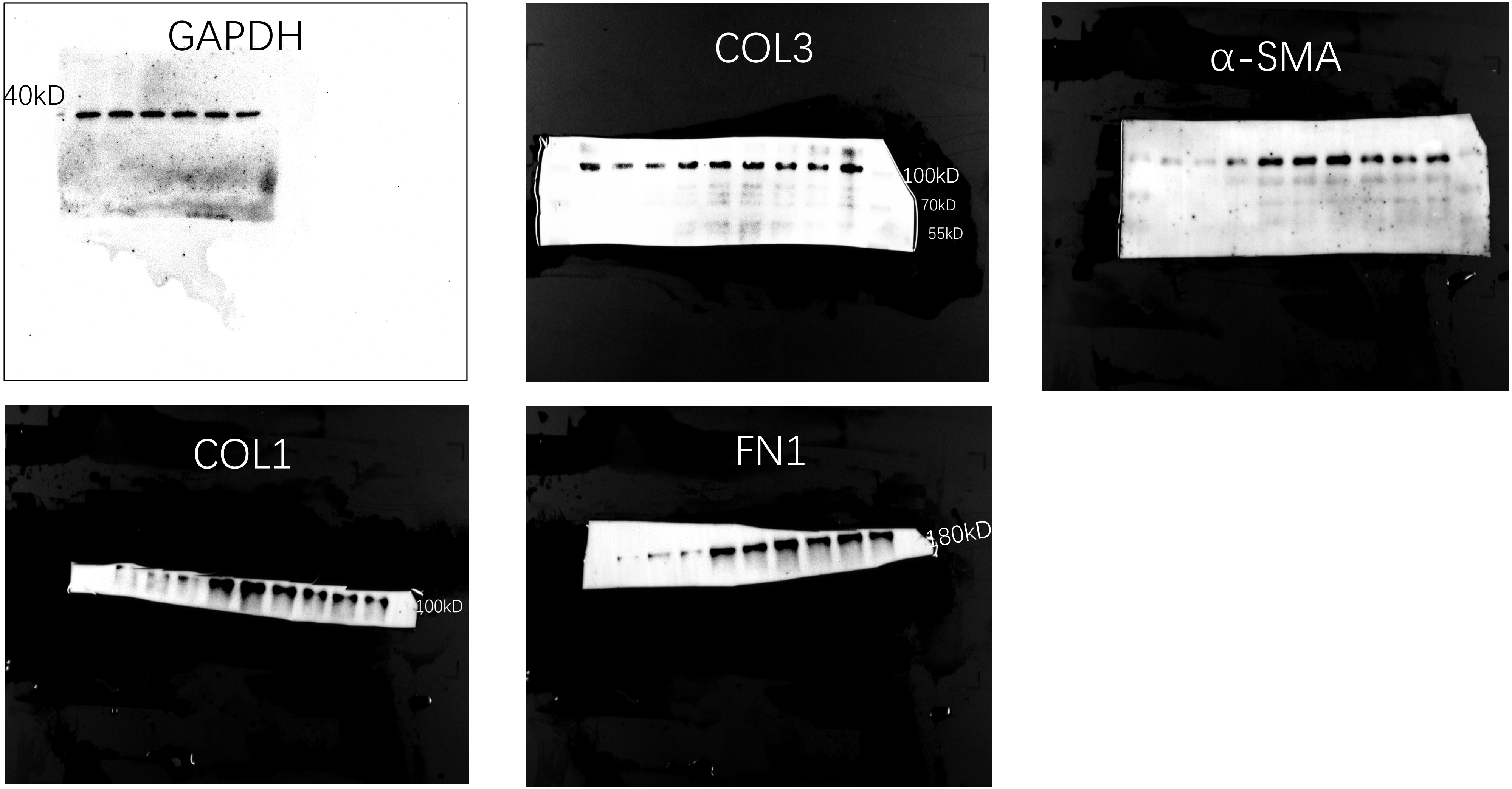
GAPDH
40kD
COL3
100kD
70kD
55kD
α-SMA
40kD
35kD
COL1
100kD
FN1
180kD

Figure 7A

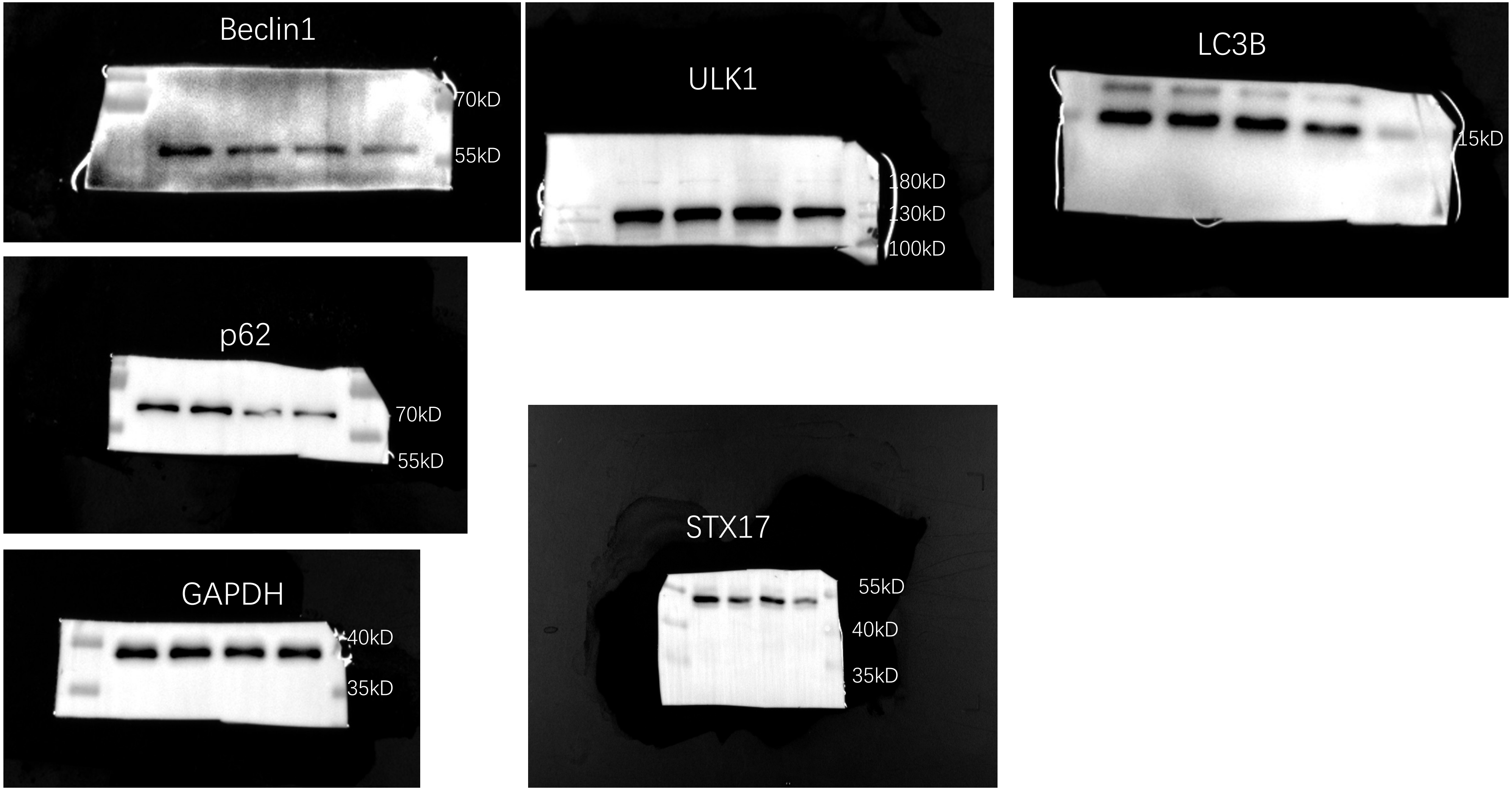
Beclin1
70kD
55kD
ULK1
180kD
130kD
100kD
LC3B
15kD
p62
70kD
55kD
GAPDH
40kD
35kD
STX17
55kD
40kD
35kD

Figure 7E

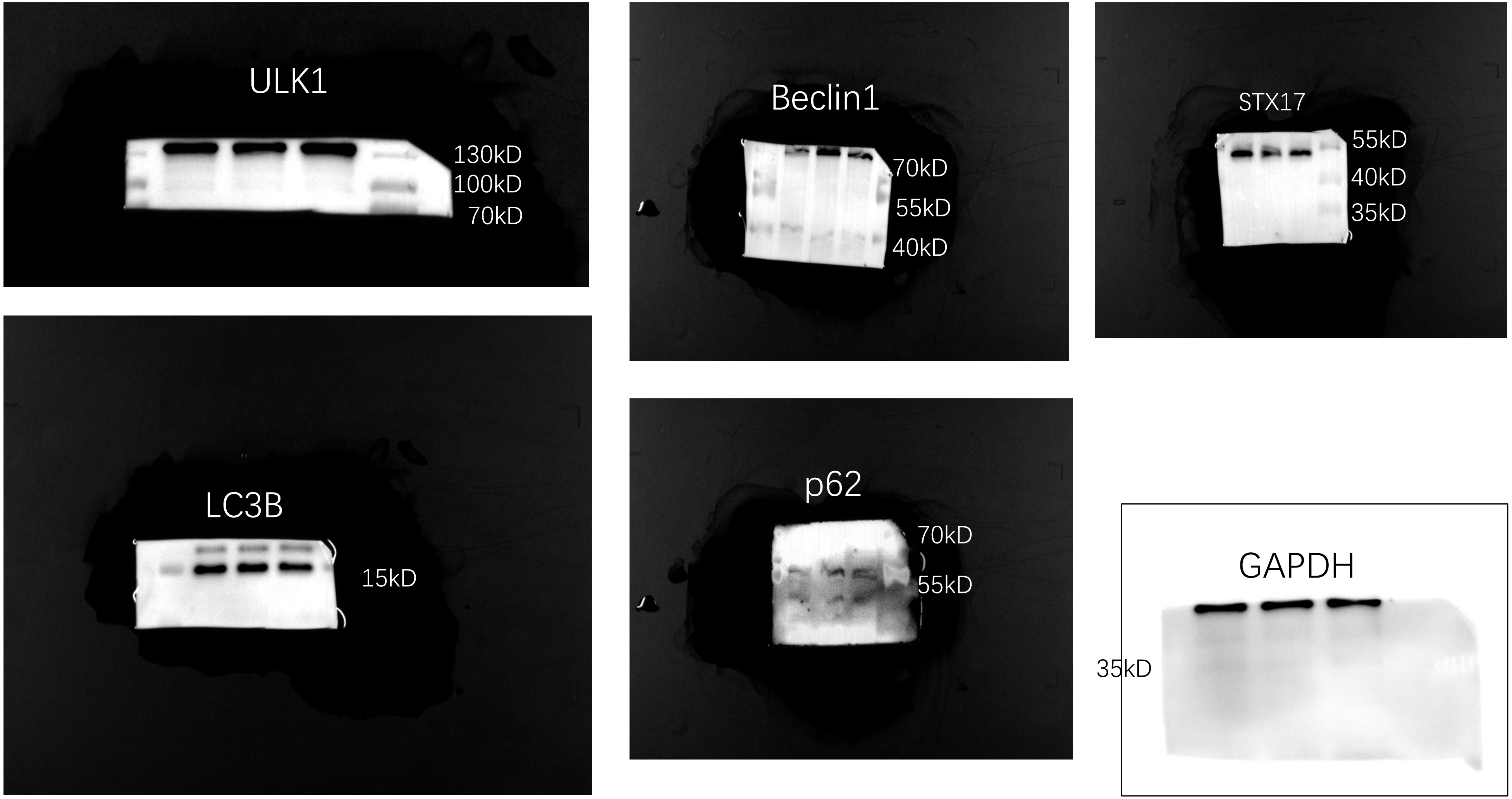
ULK1
130kD
100kD
70kD
Beclin1
70kD
55kD
40kD
STX17
55kD
40kD
35kD
LC3B
15kD
p62
70kD
55kD
GAPDH
35kD

Figure 7F

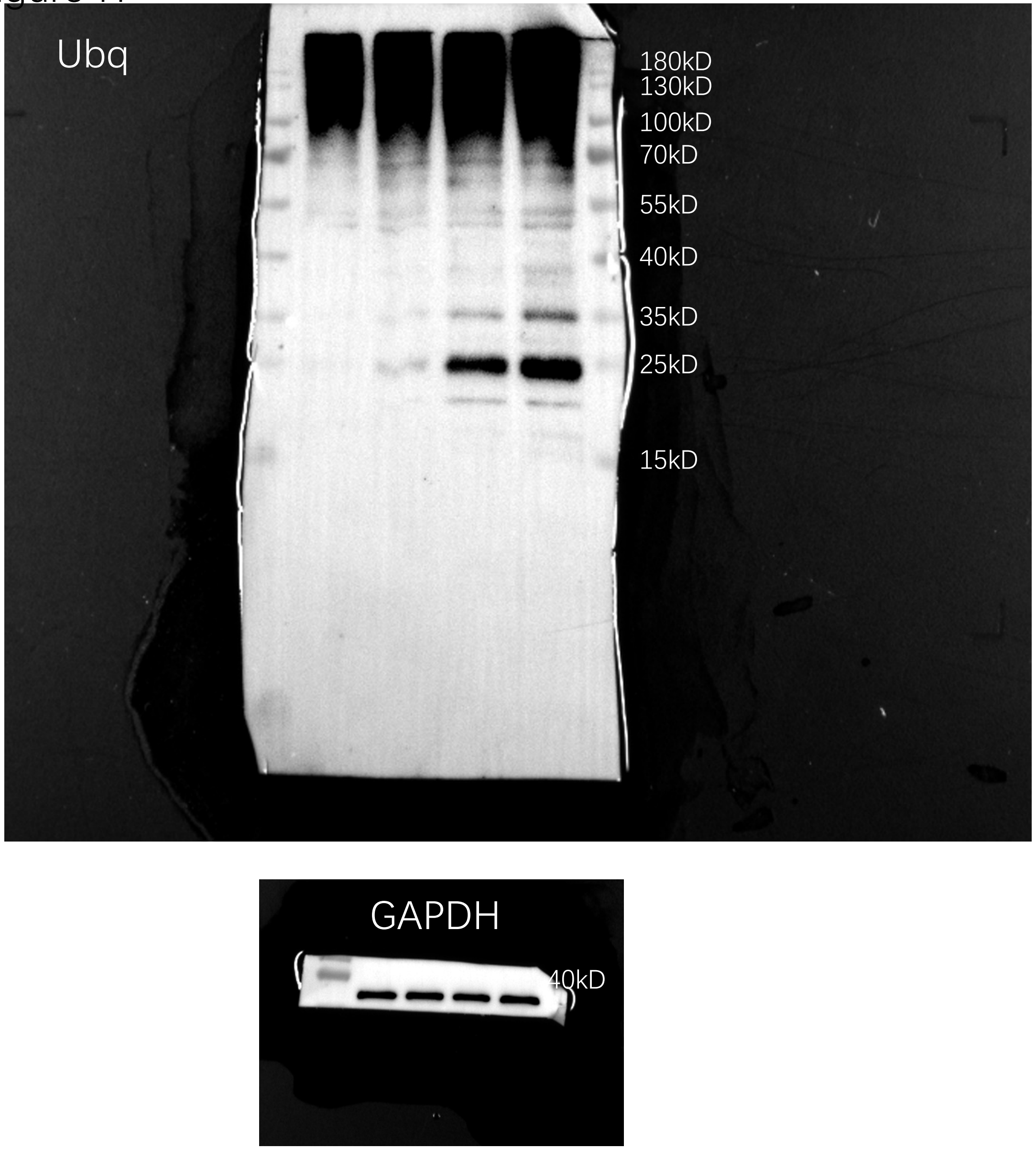

Figure 7N

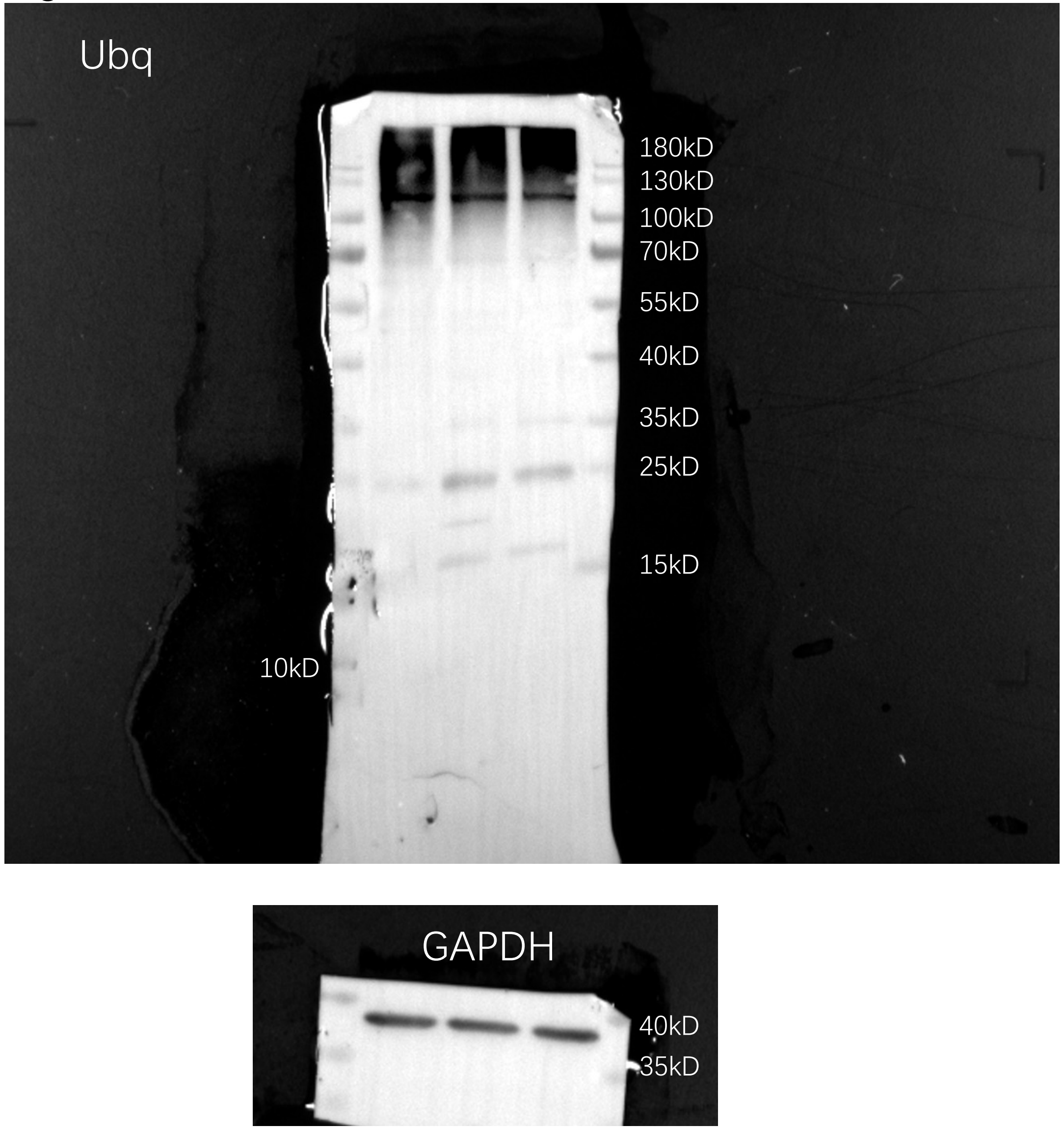

Figure 7G

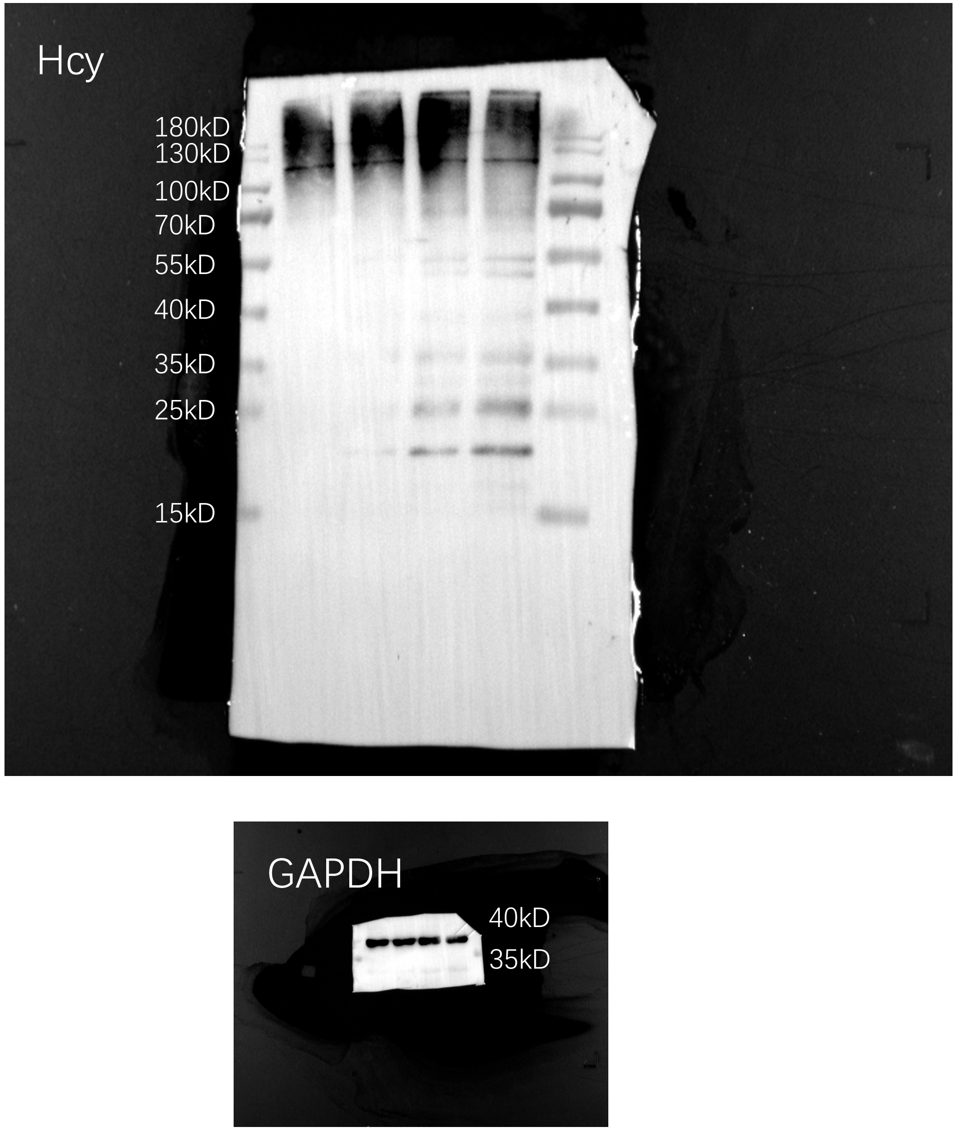

Figure 7O

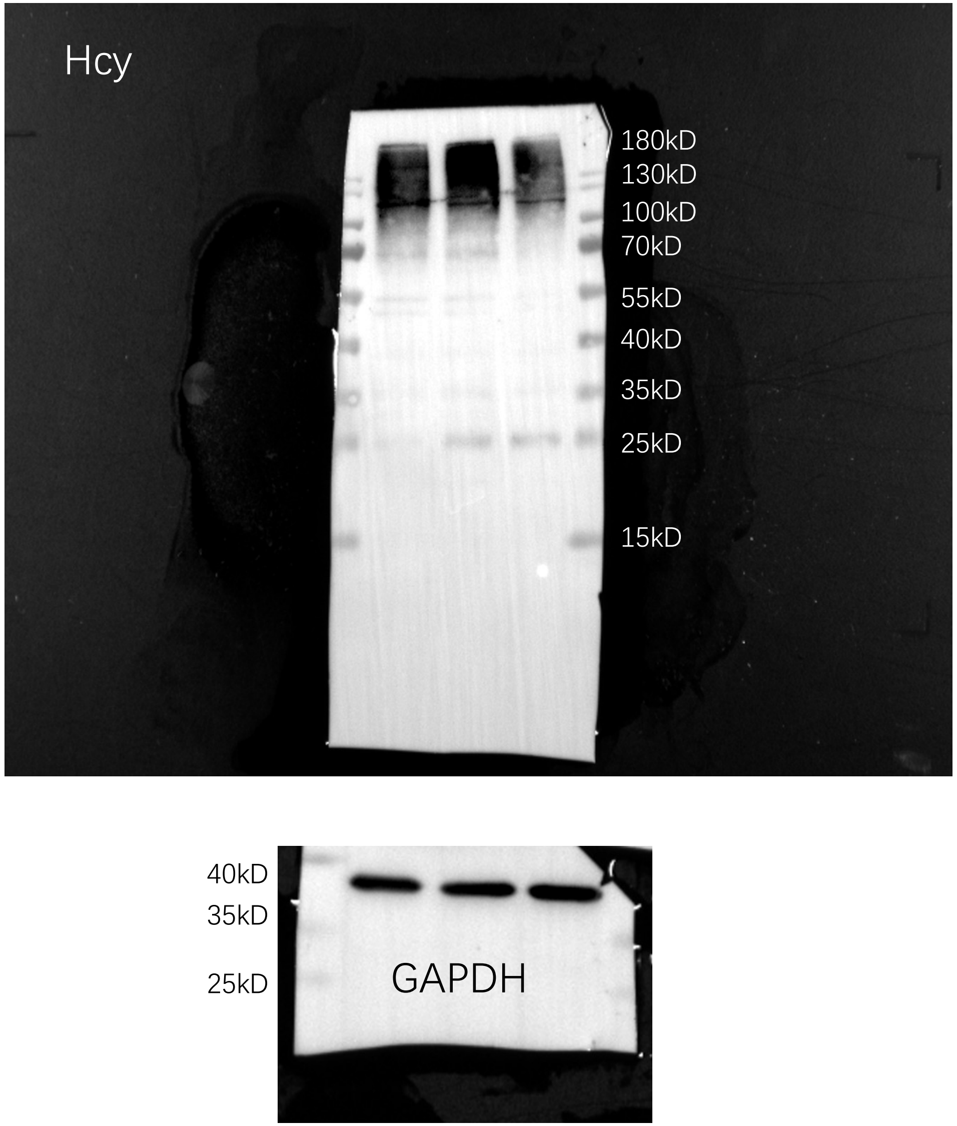

Figure 7J

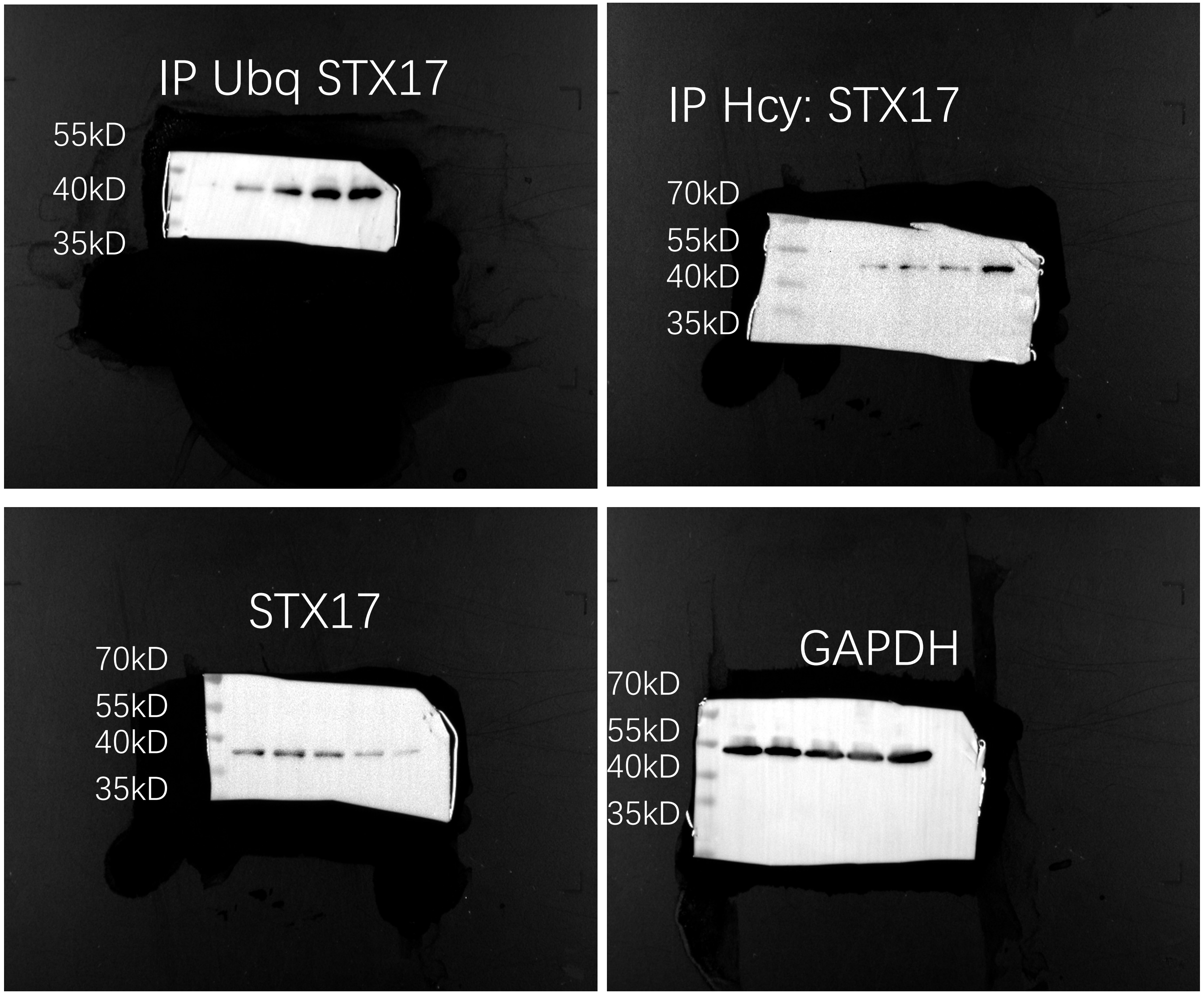
IP Ubq STX17
55kD
40kD
35kD
IP Hcy: STX17
70kD
55kD
40kD
35kD
STX17
70kD
55kD
40kD
35kD
GAPDH
70kD
55kD
40kD
35kD

Figure 7R

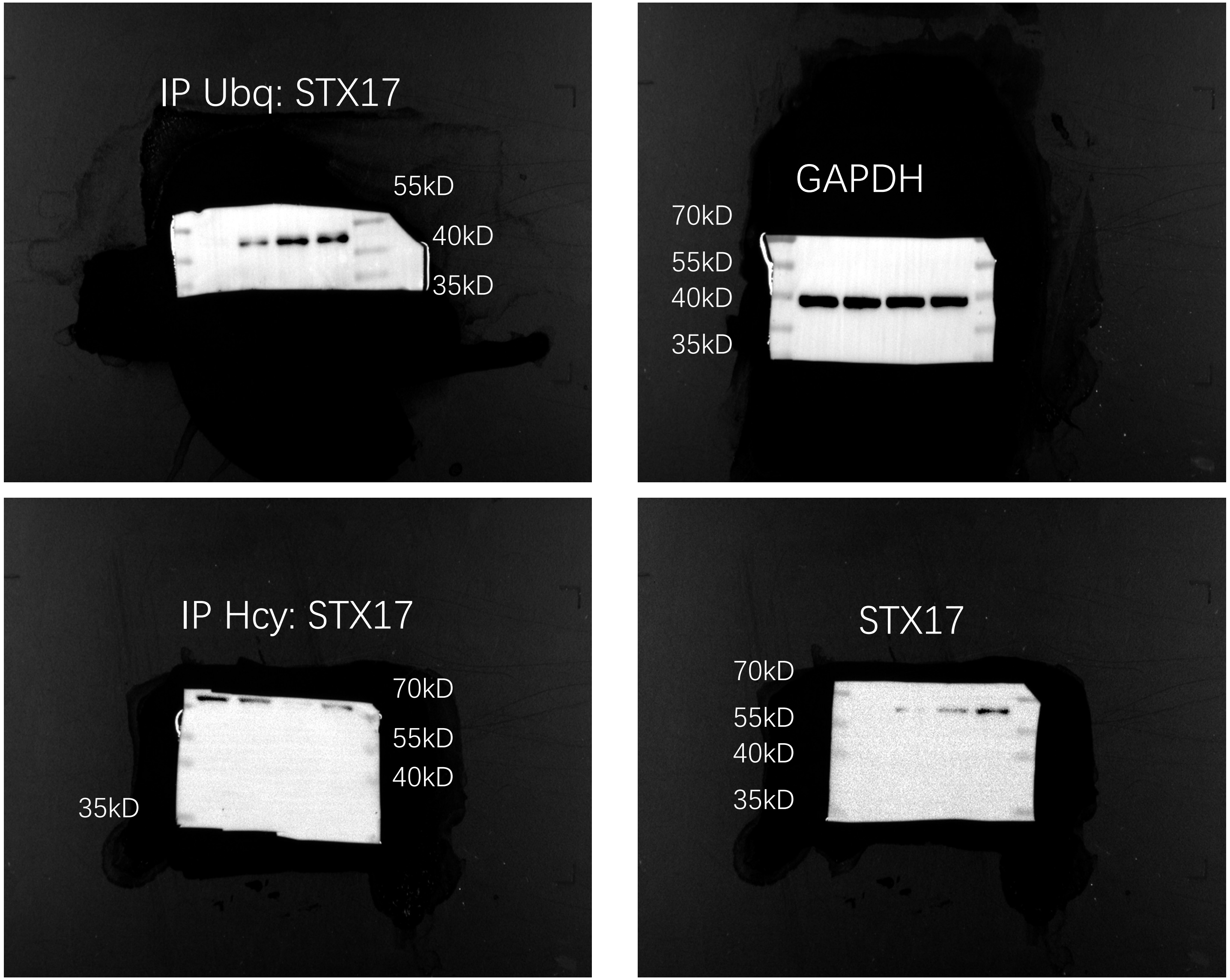
IP Ubq: STX17
55kD
40kD
35kD
GAPDH
70kD
55kD
40kD
35kD
IP Hcy: STX17
70kD
55kD
40kD
35kD
STX17
70kD
55kD
40kD
35kD

Fig S4

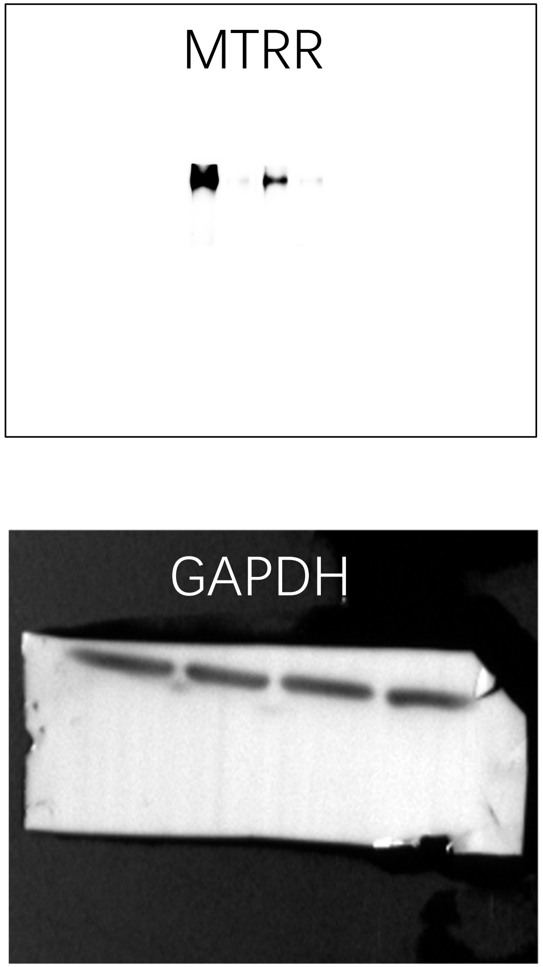

Fig S4B

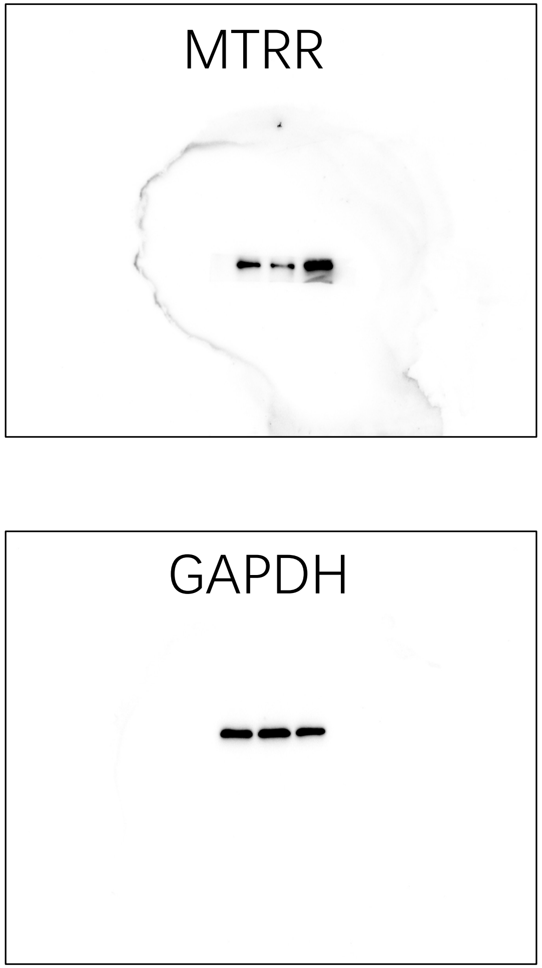

Fig S4G

Fig S7G

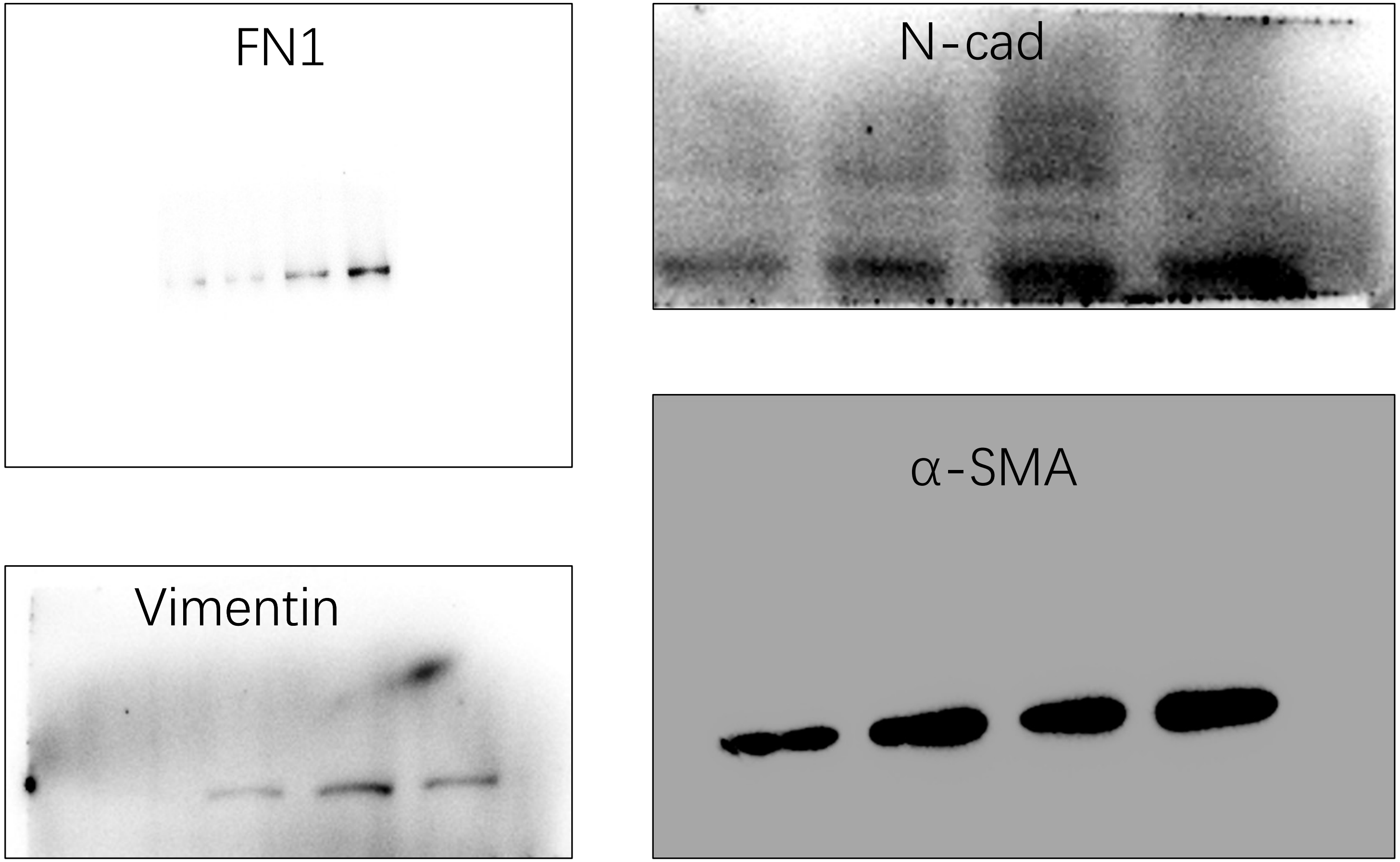

Fig S10F

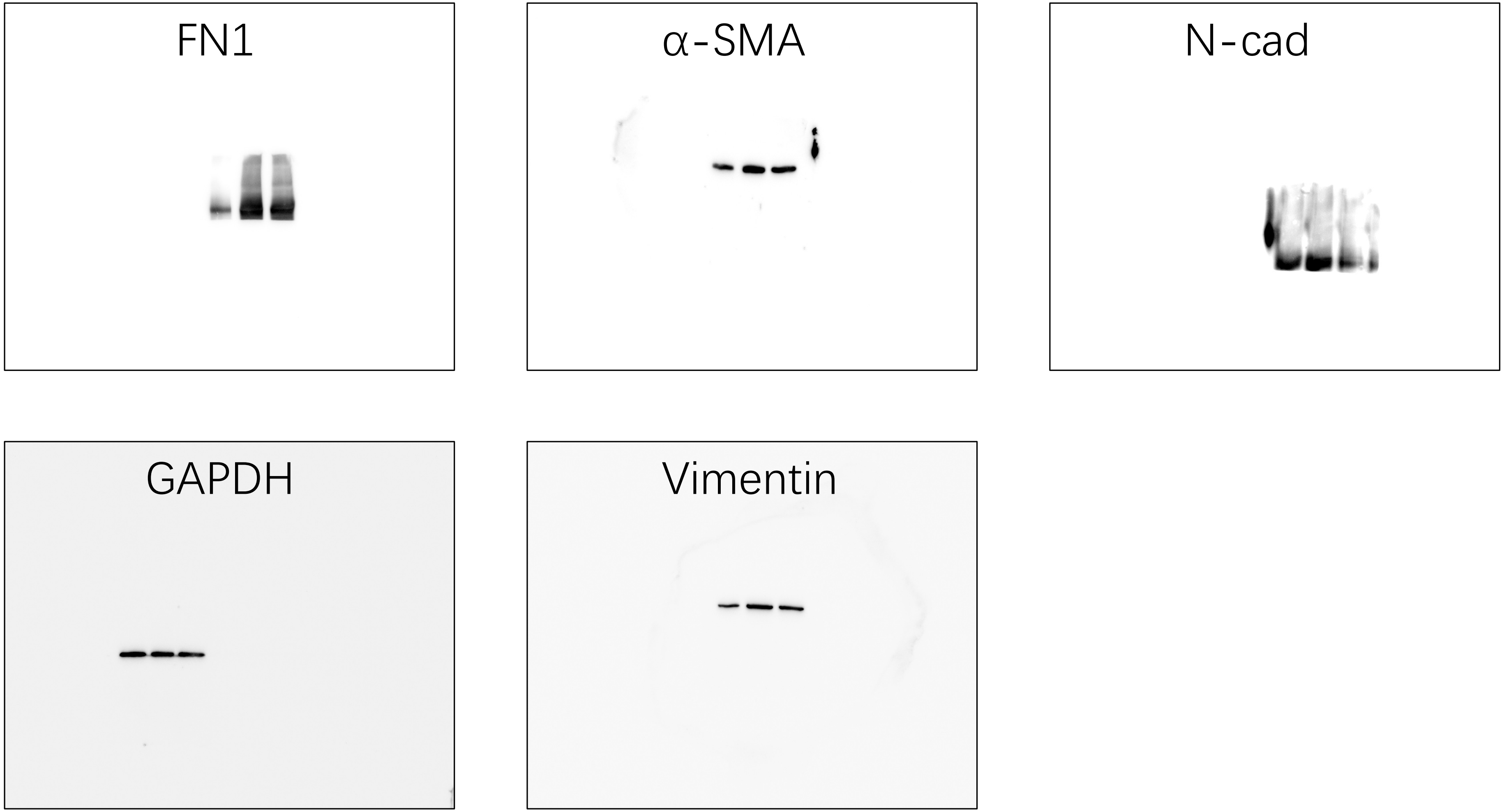

Fig S13E

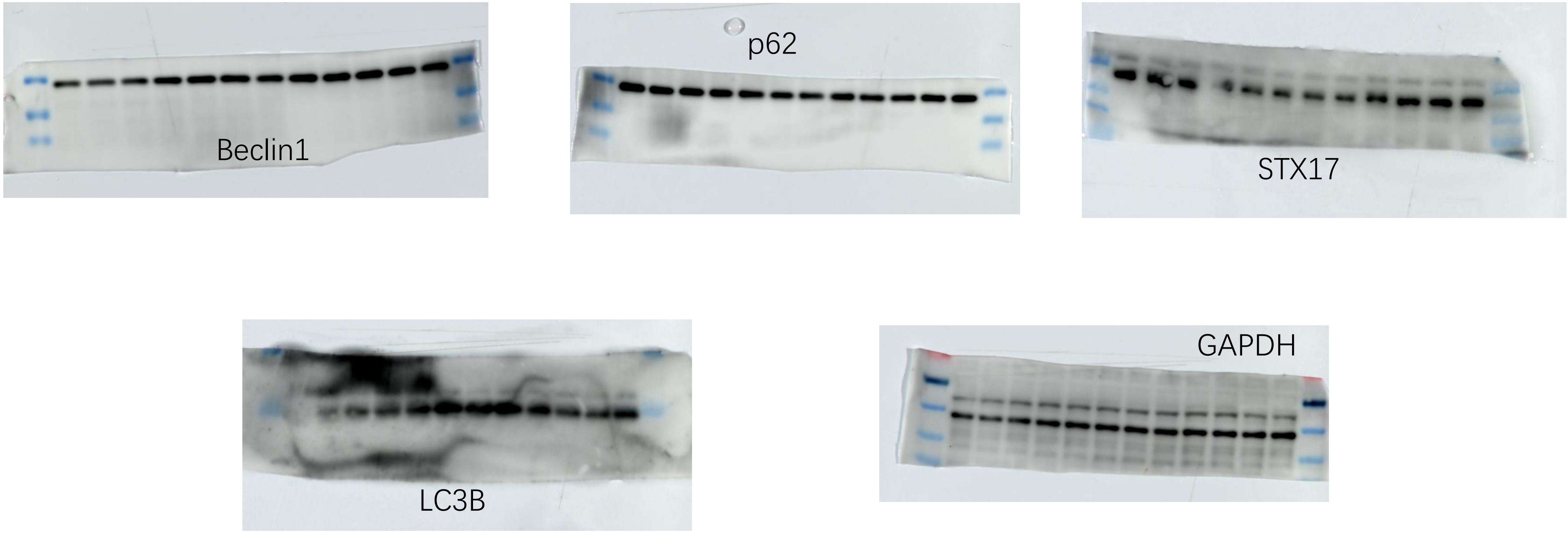

Supplement: Supplementary file 4 — Supporting Information [file ADVS-12-e07803-s003.pdf]
